# Intestinal Barrier and Permeability in Health, Obesity and NAFLD

**DOI:** 10.3390/biomedicines10010083

**Published:** 2021-12-31

**Authors:** Piero Portincasa, Leonilde Bonfrate, Mohamad Khalil, Maria De Angelis, Francesco Maria Calabrese, Mauro D’Amato, David Q.-H. Wang, Agostino Di Ciaula

**Affiliations:** 1 Clinica Medica “A. Murri”, Department of Biomedical Sciences & Human Oncology, University of Bari Medical School, 70124 Bari, Italy; leonilde.bonfrate@uniba.it (L.B.); agodiciaula@gmail.com (M.K.); agostinodiciaula@tiscali.it (A.D.C.); 2 Department of Soil, Plant and Food Sciences, University of Bari Aldo Moro, Via Amendola 165/a, 70126 Bari, Italy; maria.deangelis@uniba.it (M.D.A.); mdamato@cicbiogune.es (F.M.C.); 3 Gastrointestinal Genetics Lab, CIC bioGUNE-BRTA, 48160 Derio, Spain; mauro.damato@ki.se; 4 Ikerbasque, Basque Foundation for Science, 48009 Bilbao, Spain; 5 Department of Medicine and Genetics, Division of Gastroenterology and Liver Diseases, Marion Bessin Liver Research Center, Einstein-Mount Sinai Diabetes Research Center, Albert Einstein College of Medicine, New York, NY 10461, USA; david.wang@einsteinmed.org

**Keywords:** intestine, microbiota, metabolome, metabolic syndrome

## Abstract

The largest surface of the human body exposed to the external environment is the gut. At this level, the intestinal barrier includes luminal microbes, the mucin layer, gastrointestinal motility and secretion, enterocytes, immune cells, gut vascular barrier, and liver barrier. A healthy intestinal barrier is characterized by the selective permeability of nutrients, metabolites, water, and bacterial products, and processes are governed by cellular, neural, immune, and hormonal factors. Disrupted gut permeability (leaky gut syndrome) can represent a predisposing or aggravating condition in obesity and the metabolically associated liver steatosis (nonalcoholic fatty liver disease, NAFLD). In what follows, we describe the morphological-functional features of the intestinal barrier, the role of major modifiers of the intestinal barrier, and discuss the recent evidence pointing to the key role of intestinal permeability in obesity/NAFLD.

## 1. Introduction

The gut contains the largest surface of the human body exposed to the external environment, extending for roughly 200–300 m^2^ [1]. The “intestinal barrier” is a complex morphological-functional mechanism which involves the gut microbial barrier and mucus, gastrointestinal motility and secretion, epithelial barrier, and the immune (innate and adaptive), gut vascular and liver barrier. A healthy gut is characterized by the selective permeability of nutrients, water, and bacterial products, and processes are governed by several neural, cellular, immune, and hormonal factors. A close interaction exists between nutrients, such as dietary fiber, protein, and fat, gut microbiota producing several metabolites, such as short-chain fatty acids, lipopolysaccharides, etc., and the intestinal barrier under health or disease conditions. Obesity and nonalcoholic fatty liver disease (NAFLD) are highly prevalent, inter-related conditions at increased risk of causing advanced liver diseases and mortality. Mechanisms governing gut permeability are disrupted in both obesity and NAFLD, and this situation represents an aggravating factor in both diseases. In what follows, we discuss recent evidence pointing to the role of the intestinal barrier and permeability in health and in two pathophysiologically relevant metabolic disorders, obesity and liver steatosis.

## 2. The Intestinal Barrier as an Integrated System of Multiple Elements

The mucosal surface of the gastrointestinal tract is a major ecological niche for many microbes and an important point of entry for bacteria and bacterial products. Apart from its important role as the immune barrier, the gut is involved in the absorption of nutrients, while it also prevents invasion by several organisms. The intestinal barrier is part of the gut–liver axis, a complex mechanism characterized by the bidirectional crosstalk occurring among the microbiome, the gut, and portal vein, and the liver, biliary tract, systemic circulation, and many systemic mediators [2,3].

**Figure 1:**
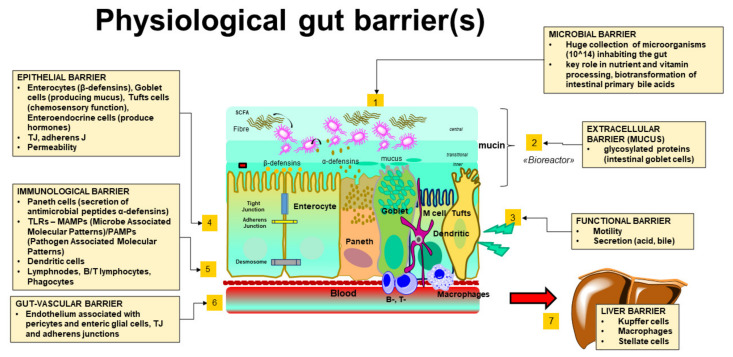
The integrated components of the intestinal barrier in physiological conditions: (1) the gut microbiota (i.e., microbial barrier); (2) the gut mucus, accumulating at the interface between the intestinal lumen and the brush border of enterocytes; (3) the interplay between gastrointestinal motility and secretions (i.e., the functional barrier); (4) the epithelial barrier and the tight junctions; (5) the immune-competent cells and their products (i.e., the immunological barrier); (6) the gut–vascular interface; (7) the hepatic filter (i.e., the liver barrier). Adapted from Di Ciaula et al. [11].

On one side, gut-derived products are transported across the intestine to the portal vein and to the liver. On the other side, liver secretes bile, especially bile acids (BA), and antibodies originate from the liver and flow across the intestine. This is a very dynamic and resilient functional system, and the mechanisms maintaining the function of the intestinal barrier do have major effects on metabolic balance in both health and disease conditions. For example, the gut microbiota undergoes continuous adaptations to lifestyle and foods and is responsible for the biotransformation of primary (hepatic) BA into secondary and tertiary (intestinal) BA, which contributes to the enrichment of the total BA pool in the body. The first physiological function of BA contributes to fat digestion and absorption and the stimulation of gut nuclear- and membrane-associated receptors [4,5,6,7]. In addition, BA controls the gut microbiome. Thus, the intestinal barrier function with the maintenance of gut homeostasis relies on the anatomical and functional integrity of the microbiome, mucus, enterocytes, immune system, and gut vascular barrier [8,9,10,11] (Figure 1).

### 2.1. The Gut Microbiota

The first level of the intestinal barrier can be identified in the gut microbiota (i.e., the microbial barrier). This first-line barrier is composed of hundreds of trillions of commensal microorganisms involved in a complex polymicrobial ecology network placed at the edge between external and internal environment [12]. This includes bacteria, viruses, fungi, bacteriophages, and protists. The universe of the gut microbiota is mainly composed of four phyla: *Firmicutes, Bacteroidetes, Actinobacteria, and Proteobacteria* [13]. In the human gut, the two major phyla include Firmicutes, which are mainly enriched in Gram-positive bacteria with facultative, anaerobic, bacilli, and cocci, and Bacteroidetes encompassing Gram-negative bacteria and, in the human gut, *Bacteroides*, *Alistipes*, *Parabacteroides*, and *Prevotella* genus primarily [14]). Microbiota density is around 10^3^–10^4^ per gram in the stomach, 10^5^–10^6^ in the jejunum, 10^8^–10^9^ in the terminal ileum, and about 10^12^–10^14^ bacteria per gram of gut contents in the colon, and it comprises over 1000 bacterial species [15]. According to recent estimates, there are three bacterial cells for every single human body cell [16]. Each bacterium has tens of thousands of ribosomes, each of which includes a copy of the bacterial RNA. There are more than ten times the genes of the human genome and the weight of the whole bacterial mass is roughly 1–2 kg [17]. Because different factors (including environment, diet, drugs, and host genetics) may impact on this “superorganism”, distinct signatures can result in terms of profound modifications in health and disease.

The gut microbiota contributes to the digestion of nutrients and their relative metabolism, such as carbohydrates and proteins, vitamins, and the biotransformation of gut primary BA to secondary BA that play a key role in gut fat digestion and also act as potent signaling hormone-like agonists in both health and disease, including obesity and NAFLD [4,5,18,19]. The host develops a natural immunity and tolerance towards the microbiota. This interaction influences the development and maturation of several cells within the lymphoid tissues of the intestinal immune system [20,21].

The polymicrobial community populates the outer mucus layer in the gut lumen since the adherence of microorganisms to enterocytes points to infection. Thus, the healthy bowel segments are normally free of bacteria, and there are mechanisms controlling the growth, composition, and organization of the microbiota in each gut segment. Secretion of gastric acid and BA, as well as luminal pH and oxygen availability, and gut motility can either suppress bacterial growth or can create a physical barrier, playing a role similar to that of mucin.

The gut bacteria produce a considerable number of volatile metabolites, and these include H_2_, methane, short-chain fatty acids (SCFAs), and alkanes, which are partly exhaled in breath. About 900 volatile compounds appear in human breath [22].

Physiologically active molecules from bacteria include SCFA, p-cresol, p-cresyl-glucuronide, indoxyl sulphate, indole-3 acetic acid, H_2_S, and trimethylamine N-oxide (TMAO) producing local effects (intestinal barrier) or acting in distant organs which include the brain, heart, kidneys, and liver [23]. Metabolites produced by gut microbiota can also influence lipid metabolism, thermogenesis, and the function of both white and brown adipose tissues [24].

Some vitamins undergo gut biotransformation by bacteria. Vitamin B_3_ (niacin) is composed of nicotinic acid and nicotinamide, and nicotinamide is converted to nicotinic acid by the microbiota. Vitamin B_5_ (pantothenic acid) is found in foods but is also produced by colonic bacteria [25]. Enteric bacteria synthesize vitamin B_12_ (cobalamin) and vitamin B_9_ (folic acid). In case of bacterial overgrowth in the small intestine, vitamin B_12_ malabsorption can develop, since the microbiota compete with the host for the absorption of this vitamin. The vitamin K group consists of vitamin K_1_ (phylloquinone), derived from food, and vitamin K_2_ (menaquinone). Most of the gut microbiota species such as *Enterobacter* sp.*, Eubacterium lentum, Veillonella* sp*.,* and *Bacteroides* sp. can metabolize K_1_ to K_2_ [26].

The microbiota can modulate nutritionally derived metabolites such as tryptophan from milk, eggs, vegetables, and red milk, with *Lactobacillus*-mediated production of indole-3-aldehyde binding the aryl hydrocarbon receptor (AHR) that mediates its transport through the epithelial cell layer by a transporter containing angiotensin I-converting enzyme 2. This step prevents gut inflammation and bacterial overgrowth [26].

Another important function of the gut microbiota includes the bacterial capacity to transform primary BA to secondary BA in the colon during the process of enterohepatic circulation [4,5,7].

The distinction between pathogenic and non-pathogenic bacteria in the colon is no longer specific, since many indigenous bacteria can act as pathogens (*E. coli, Bacteroides* sp.*, Enterococci* sp.*, Clostridium histolyticum*) if the mechanisms of protection fail and the invasion of the mucosa occurs. Notably, dysbiosis is associated with an increased population of pathogenic bacteria which likely dismiss higher levels of lipopolysaccharides (LPS) damaging the enterocytes, disrupting mechanisms of gut permeability, and predisposing the passage of LPS into the bloodstream with effects on other organs including the liver [27,28]. LPS can be taken up into chylomicrons derived from dietary saturated fats. The LPS-enriched chylomicrons promote inflammatory changes in the host for inducing insulin resistance [29].

### 2.2. The Extracellular Barrier: The Gut Mucus 

This is the second component of the intestinal barrier [30]. The mucus confers protection to the host [31,32] and consists of heavily glycosylated proteins released by the gut goblet cells, which also belong to the epithelial barrier [33]. The mucus accumulates at the interface between the intestinal lumen and the brush border of enterocytes, and its thickness increases from the stomach towards the colon, in parallel with the increase in the density of resident bacteria [32,34]. The mucus layer undergoes continuous re-arrangement, creating an onion-like stratification on the border between stool and the mucus layer. Secretory IgA, lysozyme, and defensins produced by Paneth cells interact with the inner mucus stratification and form complexes which help to keep bacteria away from the brush border of enterocytes [35]. In addition, Paneth cells dismiss antibacterial lectins such as the regenerating islet-derived protein III (REG3G) that inhibits bacterial adhesion to the mucosa. Additional antimicrobial peptides and proteins excluding bacteria include lypd8 and zymogen granule protein 16 (ZG16) [36]. In the small intestine the mucus layer is relatively thinner and less dense. The most sophisticated structure of mucus occurs in the colon as a “bioreactor” consisting of three layers: (i) A transparent outer mucus layer above the mucosa, constantly dehydrated by the epithelium. This layer is highly viscous, enriched in Muc2, is not penetrated by bacteria, and contributes to the absorption of water and nutrients [32]. (ii) The second layer is a transitional one between the mucus inner layer and the central layer. Here, the mucus is increasingly diluted by luminal fluid and can be penetrated by bacteria that can re-populate the central zone after events such as diarrhea, antibiotic therapy, and fasting. (iii) The third layer is the central zone of the bioreactor which consists of both fibers and bacteria which are stirred to contribute to fermentation. The structure of mucus glycoproteins consists of a central protein core, which is abundant in proline, serine, and threonine, O-glycosylated amines, and hexosamines perpendicularly oriented to the protein core. The result is a gel-like sieve overlying the intestinal epithelium [37].

Microorganisms use mucin as nutrients to colonize the gut lumen, and this interaction prevents the permeation of harmful and toxic bacterial products across the intestine [38]. The interaction between microbiota, mucin, epithelial barrier, and host is a complex one since the process involves the production and metabolism of several substances [39,40,41,42,43].

The microbiota contributes to the shape of mucin in many ways [44]. The degree of mucin glycosylation is partly controlled by the ratio of Bacteroides and Firmicutes [45]. The absence of dietary fiber is associated with an increase in mucin-degrading bacteria and a decrease in mucus thickness [46]. The high-fat diet disrupts the intrinsic structure of colonic mucin, as evident in mice developing liver steatosis [47,48]. Bacteria can stimulate goblet cells (the mucin-producing cells), by activating the inflammasome NLRP6 pathway [49]. Another pathway includes the activation of cell-mediated immunity via toll-like receptor (TLR)-mediated signaling [50]. Some bacteria use mucus for nutrition and contribute to modulating inflammatory changes. *Akkermansia muciniphila* is an anaerobic, Gram-negative, mucus-degrading microorganism, and its abundance is highly close to the mucus layer in the intestine [51,52,53]. If the abundance of *A. muciniphila* is decreased, some changes are associated with inflammation, impaired barrier integrity, and liver steatosis (NAFLD) [54,55].

Several abnormalities can disrupt the function of mucus, contributing to the attachment of bacteria to the enterocytes, the inflammatory changes, and the absorption of toxic substances. This is the case in ulcerative colitis, where microorganisms approach the epithelium and can contribute to the local inflammation [56], or in cystic fibrosis with the absorption of toxic substances [57]. Increased MUC2 mucin production results in the susceptibility of goblet cells to apoptosis and endoplasmic reticulum stress. Alcohol intake and cirrhosis are associated with increased mucus thickness. In mice, abnormal MUC2 in the epithelial cells results in inflammatory changes, a picture resembling ulcerative colitis.

### 2.3. The Interplay between Gastrointestinal Motility and Secretions

The interaction between gastrointestinal motility and secretions contributes to the maintenance of a healthy intestinal barrier. The oro-aboral intestinal peristalsis leads to the clearance of luminal debris, such as dead cells and alimentary residuals, and prevents the proliferation of microbiota. The gastric acidic juice, alkaline bile, and pancreatic juice contribute to gut health [4] and have antimicrobial properties [8,58]. Nevertheless, both *Helicobacter pylori* and members of the *Lactobacillus* genus can survive in the acidic environment of the stomach and small intestine [59]. A change of these conditions might lead to both qualitative and quantitative modifications of the gut microbial composition, abnormal gut homeostasis, and disease [8]. In liver cirrhosis, the decreased secretion of bile is associated with reduced normal Gram-positive microbiota, such as *Blautia* and *Ruminococcaceae,* and by an increased proinflammatory taxa, *Enterobacteriaceae* [60]. There is a close and bidirectional contact between bile, in particular BA, and the gut microbiota [61,62,63,64,65]. The ultimate composition of bile in humans depends on BA synthesis and biliary secretion in the liver, bile concentration in the gallbladder, bile release into the duodenum following the neuro-hormonal stimulation of the gallbladder, and the gut transit of bile, as well as the microbial biotransformation, gut re-absorption, and faecal excretion of BA. These steps are integral parts of the enterohepatic circulation of BA in the gut–liver axis [4,5,7].

### 2.4. The Epithelial Barrier and the Tight Junctions

This level includes the monocellular epithelial layer [66] of enterocytes which produce antimicrobial peptides -AMPs- α-defensins, goblet cells which produce mucus, tuft cells with chemosensory functions and secreting IL-25 involved in innate immunity and IL-13 with control on IgE responses and acts on goblet cells [67], enteroendocrine cells producing hormones such as CCK, VIP, GLP1, GLP2, PYY etc., and Paneth cells in crypt regions which produce antimicrobial peptides -AMPs- β-defensins and also belong to the immune barrier [68,69,70]. The “M” cells occur in the small intestine and are involved in antigen uptake and antigen-specific mucosal immune responses, such as the induction of secretory immunoglobulin A (sIgA). The monocellular layer provides defense as a physical barrier and site where innate immunity operates. The barrier is characterized by physical, electrical, and chemical properties, and is impermeable to most solutes requiring specific transporters to cross the barrier (involving the transcellular pathway). The negative charge of brush border microvilli depends on polar carbohydrates and charged transmembrane proteins, opposes the negative charge of the bacterial cell wall and inhibits bacterial translocation [71].

Cells are sealed between each other since the intercellular spaces are closed by the apical junctional complex [72]. There are three main structures of intercellular junction [72,73,74,75]: (i) tight junctions (TJs) require over 40 proteins, which include claudin, occludin, intracellular plaque zonula occludens (ZO) 1 and 2 and cingulin, and junctional adhesion protein molecule-A (JAM-A). ZO is then connected to cellular myosin and actin, and myosin light-chain kinase (MLCK); (ii) zonula adherens, which comprises β-catenin, α-catenin, and E-caderin; and (iii) desmosome, made of desmocolin, desmoglein, and desmoplakin connected to keratin.

Molecules and water pass through the epithelial barrier by transcellular and paracellular routes [76,77]. The transcellular route is designed to allow the passage of soluble lipids, small hydrophilic compounds, ions, and water. The paracellular route is controlled by the TJs controlling both active and passive transport [78]. The pore size of the barrier along the gut ranges from 4–5 Å at the villus tip to >20 Å at the base of the crypt in the small bowel. This variability is consistent with the increased gradient of enterocyte paracellular permeability from villus to crypt. In addition, the colonic epithelium is less permeable than the small intestinal epithelium. TJs regulate the passive flow of both water and solutes across the paracellular pathway, and work as a size- and charge-selective filter [79]. At this level, there are two different routes which involve: (i) the claudin-mediated pathway limiting the flow to small molecules of less than 4 Å and (ii) the leak pathway with transport of larger substances (proteins and bacterial components) up to 600 Da in in vivo and 10 kDa in in vitro. Via the TJs the human body prevents the uncontrolled translocation of substances across the intestinal barrier [80]. TJs are also regulated by factors such as cytokines (e.g., IL-13), tumour necrosis factor-alfa (TNFα), interferon gamma (IFNγ) signaling kinases, and cytoskeleton MLCK [81,82,83,84]. The IL-13 receptor activates casein kinase 2 and phosphorylates occludin, which then interact with zonula occluden-1 (ZO-1). TNFα activates the leak pathway via the MLCK and endocytosis of occludin leading to the collapse of the tight junction. Under conditions of damage of the epithelial barrier and gut inflammation, the leak pathway develops with passage of macromolecules (larger than 600 Da), bacterial products, and food antigens. Of note, impaired intestinal barrier function has been shown in animal models of obesity, diabetes mellitus [85,86], and inflammatory bowel diseases [87].

### 2.5. The Immune-Competent Cells and Their Products (i.e., the Immune Barrier)

The immune barrier is composed of immune-competent cells, which include the Paneth cells, dendritic cells, B and T lymphocytes, phagocytes, and peptides with antimicrobial effects such as defensins, cathelicidines, resistin-like molecules, bactericidal-permeability-inducing proteins and lectins, IgA. The molecules, together with MAMPs/PAMPs, accumulate within the intestinal lumen in the inner layer of the host mucin [1,10,88].

Resident intraepithelial lymphocytes are involved in host defense against pathogens, wound repair, and homeostatic interactions with the microbiota and nutrients, and bidirectionally with the epithelium [89]. Immune cells migrate from mucosal inductive tissues to effector tissues via the lymphatic system. Inductive tissues consist of the gut-associated lymphoid tissues (GALT) which include Peyer’s patches, mesenteric lymph nodes, and isolated lymphoid follicles, as part of the mucosa-associated lymphoid tissue (MALT). This migration is the cellular basis for the immune response in the gastrointestinal tract. Both cells of the adaptive immune system and innate immune system play a key role in the intestine.

Beside the classical B and T lymphocytes, there are several distinct subsets of IELs. αβ T cell receptor (TCRαβ+) subsets, unconventional TCRαβ+ and TCRγδ+ subsets, group 1 innate lymphoid cells (ILC1s), and ILC1-like cells. All cells produce interferon-γ (IFNγ) and granzyme B in response to IL-12 and IL-18 and in response to the stimulation of their natural killer (NK) cell receptors by stress-induced ligands (the latter response is absent in conventional TCRαβ+ IELs) [90]. Additional cells are interleukin (IL)-10-producing regulatory T (Treg) cells, IL-22-producing group 3 innate lymphoid cells (ILC3s), and IL-17-secreting protective T helper (Th) 17 cells and CD8αα+ T cells [91]. The lymphocytes act as the first line of cytolytic defense by producing type I cytokines and releasing antimicrobial peptides in response to cytokines released by specific gut epithelial cells [90]. In this context the M (microfold) cells take up antigens from the gut lumen and present the antigens to the dendritic cells.

For the innate immune system, CD103+ and CD11b+ dendritic cells transport the antigens to the Peyer’s patch or via draining lymphatics into the MLNs for initiation of mucosal T and B cell response. Mononuclear phagocytes have protrusions which act as direct sensors of the gut lumen [92,93] and develop oral tolerance after delivering food antigenic peptides to dendritic cells in the lamina propria [94]. CX3CR1+ MHCII+ macrophages are also present, as well as IgA+ antibody-secreting cells (ASC) in the lamina propria with release of secretory IgA (SIgA), which contact the bacteria.

When the lamina propria is disrupted, immune-competent cells such as CD4+ T lymphocytes, innate-like cells (iNKT), and mucosa-associated invariant T cells in gut lumen are involved in tissue regeneration and efficiently recognize metabolites and antigens of microbial origin [95,96,97].

Th17 cells, a subset of CD4 T cells, are characterized by the production of IL17-A, IL17-F, and IL-22. These molecules contribute to the efficiency of tight junctions and stimulate epithelial cell regeneration [98]. Production of IgA and the polymeric immunoglobulin receptor (pIgR) allows their translocation towards the gut lumen [99,100]. The accumulation of Th17 cells is promoted by the presence, in the gut epithelium, of segmented filamentous bacteria [100,101,102].

CD4+Foxp3+ regulatory T-cells (Tregs) is a subset of helper T cells of thymic and peripheral origin and regulate peripheral tolerance, recognizing self-antigens and controlling the function of autoreactive T cells. [103]. The peripheral-derived Tregs are able to recognize antigens of food origin in the small intestine and antigens of microbial origin in the colon [103].

Innate lymphoid cells interact with innate and adaptive immune cells and receive combined signals from the gut epithelium and microbiota. These cells are involved in tissue repair and metabolic homeostasis, and release type 1, 2, and 3 cytokines following an infection, preceding the response of adaptive T cells [104,105].

Pattern-recognition receptors (including the toll-like receptors, TLRs, and the nucleotide binding oligomerization domain-like receptors) are proteins able of recognizing MAMPs, PAMPs, or molecules released by damaged cells (Damage-Associated Molecular Patterns—DAMPs). These proteins allow gut/microbiota interactions and the recruitment of dendritic cells in case of damaged intestinal barrier. Dendritic cells, in turn, transport antigens to the MLNs for antigen presentation and priming and maturation of B and T lymphocytes, as part of the adaptive intestinal immune response of the lymphoid tissue [106,107]. Thus, as a result of these complex interplays, gut microbiota plays a critical role in the maturation of immune cells [105,108].

### 2.6. The Gut-Vascular Interface

This is a further level of protection and shows similarities with the blood–brain barrier [109,110]. The main task of this barrier is to prevent the translocation of bacteria and/or microbial products in the zone between the epithelial barrier and the extracellular space, therefore avoiding the dissemination of bacteria to the liver and spleen, where additional barriers operate [109]. At this level, the “gut vascular unit” consists of the gut endothelium enriched with active and passive transporters, the associated pericytes and enteric glial cells which contribute to gut homeostasis gut permeability, TJs, and adherens junctions. This area is permeable to most of the small nutrients [109,110,111]. The intact endothelium is designed to allow the free diffusion of small molecules such as 4 kD dextran, while 70 kD dextran is blocked. Infections such as *Salmonella enterica* serovar *Typhimurium* initiate the disruption of the gut vascular barrier and promote an increased permeability. This condition allows the transit of larger molecules/microorganisms, even in the absence of inflammation and vasodilation. Dissemination involves the portal circulation, more than lymphatic vessels. The vesicle-associated protein-1 (PV1) is a marker of endothelial permeability and can increase during such events with bacteria found systemically [109]. Conditions associated with the disruption of the gut vascular barrier include anchylosing spondylitis [112], celiac disease [109], and non-alcoholic steatohepatitis (NASH) [113].

### 2.7. The Hepatic Filter (i.e., the Liver Barrier)

The liver represents the last barrier for microorganisms entering the bloodstream due to gut mucosal damage and impaired surveillance activity from mesenteric lymphnodes (MLNs) [2,114,115]. In healthy subjects, the translocation of small amounts of bacteria and bacterial products from the gut to MLNs contributes to the stimulation of the immune system and to the development of immune tolerance [116,117]. As a consequence, microbes are killed without significant systemic inflammatory modifications [118,119]. Small amounts of bacterial mRNAs and LPS [2,120,121] can reach the liver and contribute to detoxification of bacterial products [114,115]. A major function in the maintenance of the liver barrier depends on liver macrophages (Kupffer cells). These cells at the hepatic sinusoids represent roughly 80% of all tissue macrophages and are essential to phagocytize and kill bacteria from the bloodstream [2,114,122,123,124], to clear MAMPs and PAMPs, and to process *E. coli* endotoxin [125]. The activation of Kupffer cells by lipopolysaccharide is mediated by the lipopolysaccharide binding protein (LBP) and is dependent on a functional toll-like receptor 4 (Tlr 4) [126,127]. The LPS-LBP complex, in turn, stimulates liver-resident myeloid cells via mCD14 and TLR4 [128,129,130].

## 3. Assessment of Intestinal Barrier In Vivo

Several nondefinitive techniques are being used to study the modification of the paracellular pathway in vivo. Indirect methods range from orally ingested probe molecules (sugars) to circulating endotoxins, which include LPS and LPS-binding, serum zonulin, and intestinal fatty acid-binding protein levels (I-FABP) [37]. More direct methods include endoscopic assessment using confocal microscopy, and mucosal impedance. By using orally administered sugar probes and distinct urinary secretion and collection, several studies have compared the feasibility of this methodology in healthy subjects, as well as in patients with irritable bowel syndrome and in obese and NAFLD patients with evidence suggesting the presence of altered colon permeability in patients, but not in controls [131,132,133,134,135,136].

## 4. Modifiers of the Intestinal Barrier

A general overview of this scenario must consider that dietary fibers, fats, and BA can induce profound effects on intestinal permeability and microbiota. Dietary habits account for about 20% of microbial diversity in humans. Events are deeply correlated and do have reflections on metabolic aspects including obesity and liver steatosis. The protective effects of dietary fiber on the intestinal barrier are associated with homeostasis of gut microbiota. Additional agents involved in this homeostatic control of the intestinal barrier include SCFAs and epithelial IL-18. Fats appear to promote an increase in epithelial permeability, and fatty acids directly impair the epithelial barrier. Fats might further act via the release of LPS and permeation across the disrupted intestinal barrier with increased levels in serum. The role of a westernized fat-enriched diet lacking in fibers needs to be investigated with attention on intestinal barrier function. The role of *A. muciniphila* with a protective effect on intestinal barrier integrity needs to be investigated in the complex scenario connecting diet, microbiota, intestinal barrier, and metabolic disorders such as obesity and NAFLD. Research is trying to dissect the precise role of other aspects involving tight junctions, immune system, and environment. The concept of “leaky gut syndrome” requires further evaluations to establish the ultimate translational and clinical role of this condition.

### 4.1. Fibers

A huge number of engaged bacteria (more than 100 trillion) [137] can digest many fibers due to the high number of glycoside hydrolases (more than 250) which break down various types of carbohydrates [138]. Fibers are broadly classified as dietary fibers (from whole food) and synthesized functional fibers [139]. Dietary fibers are either insoluble (cellulose, some hemicellulose, and lignin [140], which act mainly as mass-forming agents in gut transit [141]) or soluble fibers which include wheat dextrin, pectin, gums, β-glucan, psyllium, and fructans, as well as some hemicellulose [140] from grains, fruits, vegetables, and legumes [142]) (Table 1). The dietary (soluble) fibers resistant to digestion by the host become microbiota-accessible carbohydrates (MACs) [143].

Dietary fibers and the MACs mostly highly fermentable by the gut microbiota, contribute to the health of individuals by increasing satiety, improving metabolic disorders, increasing the availability of SCFA with effects on gut-related immunity, endocrine function, and intestinal barrier integrity. In the mice model a diet deprived of MACs aggravated the DSS-induced colitis and increased gut permeability while reducing serum IL-18 levels [144]. The secretion of the ileal entero-hormones GLP-1 and GLP-2 ameliorates gut injury and improves gut healing [145,146]. Notably, removal of MACs in mice decreases GLP-1/2 and increases gut permeability [147].

In the rat cecum, the fructo-oligosaccharides obtained by degradation of inulin, a water-soluble dietary fiber, promote IgA production and counteract the decrease of gut permeability induced by a MACs-deficient diet [148]. The translational value of such observations requires further attention as some, but not all, clinical studies show the beneficial effects of MACs. Fructo-oligosaccharides and polydextrose protect intestinal barrier function in healthy subjects or pancreatitis patients [149,150], while administration of oat β-glucan, arabinoxylan (soluble hemicellulose) has no effects on acute indomethacin-induced gut hyperpermeability [151,152]. Similarly, inulin (enriched in oligofructose) is ineffective in celiac patients [151,152].

#### 4.1.1. Fibers as Source of Microbiota-Derived Short-Chain Fatty Acids (SCFA)

Soluble fibers undergo bacterial digestion into SCFAs as metabolites [29,153,154,155,156,157] mainly acetate, propionate, and butyrate in a molar ratio of 60:20:20 [158]. This process does not include psyllium and gums. Acetate is converted to butyrate and used by colonocytes [159,160]. SCFAs are then absorbed by colonocytes via simple diffusion or transported by the solute carrier family 16 member 1 (SLC16a1) and SLC5a8 [161]. At this level, SCFAs play a key role in cellular metabolism and immunity, and therefore contribute to maintaining the healthy function of the intestinal barrier. SCFAs also play a role at a more systemic level, when considering the gut–liver axis and energy production. At the intestinal level, acetate, butyrate, and propionate are converted to acetyl-CoA or propynyl-CoA via acetyl-CoA carboxylases (ACSSs) or β-oxidation to produce ATP. This product maintains cell homeostasis including the function of TJ [162].

In vitro, the three SCFAs promote intracellular permeability and the mechanism involves the modification of TJ expression or distribution and ZO-1 [163].

Acetate activates nucleotide-binding oligomerization domain 3 (NLRP3) and promotes the secretion of IL-18 from epithelial cells, which contribute to maintaining TJ function. 

SCFA potently stimulate ANGPTL4 (fasting-induced adipose factor, Fiaf) in human colon cell lines via PPARγ [164].

Butyrate provides energy and keeps the integrity of colonocytes [165], suppresses cytokine-induced barrier dysfunction acting on in vitro levels of claudin-2 [166], and increases mucin expression [167]. In addition, butyrate regulates hypoxia-inducible factor-1, which modulates the efficiency of epithelial TJ *CLDN1*, the gene coding for claudin-1 [168,169].

Administration of butyrate to patients with ulcerative colitis is associated with decreased faecal calprotectin, a marker of gut inflammation [170]. Studies in the animal model show that acetate in gut epithelial cells can directly activate nucleotide-binding oligomerization domain 3 (NLRP3) inflammasome with increased release of IL-18 [144], engaging the epithelial IL-18 receptor and promoting intestinal barrier integrity [171]. Propionate, in the mice colon, can counteract the increase of dextran sulfate sodium (DSS)-induced leaky barrier via downregulation of ZO-1, occludin, and E-cadherin expressions in the colonic tissue in mice [172]. SCFA become a substrate for gluconeogenesis, resulting in the modulation of central metabolism, and signal to the host by inhibiting histone deacetylase (HDAC) or by activating G-protein-coupled receptors (GPR41, GPR43, and GPR109A) [173], which triggers the release of the hormone glucagon-like peptide-1 and PYY (independently of the BA-GPBAR-1 pathway) [29]. GLP-1 slows gastric emptying and gut transit, helps energy absorption [174], and enhances glucose-dependent insulin release [175]. SCFA can activate the nuclear factor-κB signaling pathway via toll-like receptors (TLRs), which regulates the integrity of gut epithelial cells [144]. In addition, intestinal T cells are influenced by SCFA via the signaling pathway which includes HDAC and GPR43 [176,177]. The protective role of SCFA at the level of the intestinal barrier stems from additional evidence in vitro and in vivo. T84 and Caco-2 cell cultures exhibited prompt enhanced barrier function in response to physiological concentrations of SCFAs by a mechanism involving the plasma membrane cholesterol-rich microdomain [178]. When colonic organoids from human colonic mucosal biopsies were challenged with the fermentable substrate 2’-O-fucosyllactose, the increase of bifidobacteria increased SCFAs (especially butyrate). This step resulted in the upregulation of claudin-5, a marker of enhanced barrier function [179]. Shift workers prone to circadian oscillation showed a decrease in gut-derived plasma SCFAs. This finding correlated with increased colonic permeability [180].

At a systemic level, SCFA have a key metabolic activity, since from the intestine they travel to the liver via the portal circulation to provide the energy source used in lipogenesis and gluconeogenesis [181,182,183]. Acetate bypasses the splanchnic circulation and is converted into acetyl-CoA in peripheral muscles for lipogenesis or oxidation. Propionate is mainly used for liver gluconeogenesis. Some bacteria species intake lactate and succinate and convert them into propionates. Notably the intake of a MACs-rich diet in humans is associated with increased content of faecal SCFA [184]. SCFAs are an important source of ATP and contribute to maintenance of the intestinal barrier.

There is a close interaction between diet and gut microbiota in governing the production of SCFA. In an ideal situation, the higher the microbiota diversity, the more enriched the diet is with many types of complex carbohydrates which are amenable to digestion by the microbiota. By contrast, the lower the microbiota diversity, the lower the percentage of these complex carbohydrates available for microbiota. As a consequence, the production levels of certain SCFA, such as propionate, might increase. However, in the case of a decreased diversity of the microbiota, several functions will decrease or become impaired, including metabolic effects. Preserved increased diversity of the microbiota, by contrast, will provide multiple types of SCFA and additional gut microbiota diversity [29]. Evidence points to a close interaction between microbiota, fibers, and SCFA. An abundance of *Bacteroides* spp. is associated with the production of propionate [185] and acetate [186], while butyrate is produced mainly by Firmicutes phylum [186]. Colonic fermentation of fibers decreases pH levels, increases faecal acidification, and increases the growth and diversity of the gut microbiota taxa [187]. Patients with type 2 diabetes mellitus have a reduced abundance of butyrate-producing bacteria [188]. SCFAs supplementation in patients with type 2 diabetes mellitus increases the abundance of butyrate-producing bacteria, GLP-1, and haemoglobin A1c levels [189]. Further and specific aspects of SCFAs are discussed in the section relative to NAFLD.

#### 4.1.2. Additional Effects of Fibers

Inulins are naturally occurring polysaccharides also classified as fructans found in many types of plants and are industrially extracted, especially from chicory. Inulin is considered a prebiotic and produces several metabolic and bacterial effects in the body. In the rat model, Singh et al. [190] demonstrated that inulin in the range 0–25% in diet dose-dependently decreased caloric intake; improved glucose tolerance; increased the abundance of Bacteroidetes and *Bifidobacterium* spp.; decreased *Clostridium* clusters I and IV; increased butyryl-CoA:acetate CoA-transferase in cecum; upregulated peptide YY, cholecystokinin, and proglucagon transcripts in the cecum and colon; and increased plasma peptide YY and glucagon-like peptide-1 concentrations. Importantly, inulin at 25% attenuated the reduction in energy expenditure associated with calorie restriction and decreased adiposity. These findings show that inulin dose-dependently decreased caloric intake, modulated gut microbiota, and upregulated satiety hormones, with metabolic effects being largely independent of caloric restriction [190]).

In constipated patients, inulin induced the increase in *Anaerostipes*, *Bilophila*, and *Bifidobacterium* genus [191]. Some bacteria such as *Bilophila* spp. produce softer stool and improve quality of life in constipated patients [191]. The abundance of fiber-degrading bacteria decreases in patients with type 2 diabetes mellitus [188,192]. 

Microbiota-accessible carbohydrates (MACs) modulate the gut microbiota. In mice, a low-MACs diet increases levels of *Bacteroides thetaiotaomicron*, affecting the gut mucus glycans [143]. In germ-free mice on a low-MACs diet, the microbiota transplant increases mucin-degrading bacteria (*B.* *thetaiotaomicron* and *A. muciniphila*). *A. muciniphila* represents 1–4% of human gut microbiota [193] and decreased levels of *A. muciniphila* have been reported in subjects with inflammatory bowel disease [193,194]. In animal models, administration of *A. muciniphila* ameliorates DSS-induced colitis and protects the gut barrier function [195]. In mice with chronic colitis, the administration of *A. muciniphila* decreased spleen weight, colon inflammation index, colon histological score, and expression of the pro-inflammatory cytokines TNF-α and IFN-γ [196]. In mice, administration of pasteurized *A. muciniphila* or of a specific outer membrane protein (Amuc_1100) components from *A. muciniphila* improved colitis through effects on CD8+ cytotoxic T lymphocytes [197]. Highly abundant pili-like protein Amuc_1100 of *A. muciniphila* increases intracellular permeability, activates toll-like receptor 2 (TLR2) and TLR4, and cytokine production from peripheral blood mononuclear cells in vitro [198].

### 4.2. Polyphenols and Other Metabolites

Fruit and vegetables contain metabolites such as polyphenols, including flavonoids and stilbenes. Mechanisms of protection include the enhancement of the intestinal barrier function and inhibition of intestinal dysbiosis. In principle, several products can provide a diet enriched in polyphenols [199] but clinical studies are still lacking. Resveratrol was protective in the mice fed with a high-fat diet [200] and tested by the serum LPS and FITC-dextran test. Galangin is a flavonoid whose heat-induced inactivation prevented the protective effect on mucosal barrier in the rat intestinal epithelial cell (IEC-6) model [201].

The non-steroidal anti-inflammatory drug indomethacin is responsible for the decrease of ZO-1 and occludin expression in the TJ, an effect inhibited by quercetin [202]. The protective effect of quercetin on intestinal barrier function was confirmed in Caco-2 cell culture which showed assembly of ZO-2, occludin, and claudin-1 and the expression of claudin-4 [203].

Anthocyanin pigments are antioxidants which belong to flavonoids, provide colors in fruits, and appear in high concentrations in berries (elderberries, black berries, raspberries, purple corn, and black carrots), red grapes, and red wine. Depending on the food, concentrations of anthocyanins range from 50 to 1500 mg/100 g fresh weight [37]. The protective effect on the intestinal barrier was evident in the mouse model of obesity [204].

In humans, the combined intervention of the New Zealand black currant anthocyanin-rich extract with physical exercise improved the oxidative stress and likely intestinal barrier [205]. Additional protective effects are anticipated for the antioxidant polymer ellagitannins, normally ingested as punicalagin, pedunculagin, and sanguiin, which also show antimicrobial, anticancer, antinutritional, and cardioprotective properties [206]. The content of ellagitannins in pomegranate and walnuts ranges from 150 to 1600 mg/100 mL and 100 g, respectively. In support of this, the polyphenols contained in pomegranate peel reduced the high-fat diet effect in the rat model on chronic low-grade inflammatory responses. By modulating gut microbiota, the authors documented reduced circulating endotoxin and colonic inflammation. In addition, the two microbiota metabolites of pomegranate ellagitannins punicalagin and urolithin A counteracted the LPS-induced inhibition of tight junction protein expression and inflammation in Caco-2 cells [207]. Distinct effects are anticipated for the punicalagin metabolite ellagic acid which improves the intestinal barrier, while urolithin A protects against the inflammation-induced barrier dysfunction [208].

### 4.3. Glutamine

Glutamine is the L-alpha-amino acid most abundant in blood in humans. It can be synthetized and obtained from diet and enters the protein synthesis or energetic pathways. Glutamine can act as a nitrogen donor in intracellular metabolism. Protective effects of glutamine have been shown on tissue integrity, inflammation, and intestinal permeability in IBS patients [209] and Chron’s disease [210]. A review of foods and supplements containing glutamine and their effects on health addresses this issue [211].

### 4.4. Vitamin D and Zinc

Vitamin D levels improved in IBS patients with increased intestinal permeability on a low-FODMAP diet [212]. Vitamin D administered to Chron’s disease patients ((2000 IU/daily) for 3 months was associated with improved permeability in the gastroduodenal tract by a sucralose test but not in the small intestine (lactulose, mannitol ratio) or colon [213].

The food supplement zinc carnosine appears to protect the intestinal barrier via a proliferative response, as shown in the cell culture model and a clinical study using the lactulose to rhamnose ratio [214].

### 4.5. Probiotics, Symbiotics and Prebiotics

The ultimate role of these treatments requires further studies. Probiotics and synbiotics enhance intestinal barrier function in response to stressor or disease states. Clinical outcomes must be investigated [37,215].

### 4.6. Fats

Diet provides continuous delivery of fats such as triglycerides and cholesterol which are digested and absorbed in the gut lumen. Starting from dietary triglycerides and following BA-induced emulsion and formation of FFA+BA micelles, FFA undergo further assembly in the enterocytes [216]. 

Dietary FFA are about 15% of the total pool of FFA in the body and are divided into saturated or unsaturated fatty acids. According to the chain length, FFA are classified as short-chain (SFCAs), middle-chain (MCFAs), and long-chain fatty acids (LCFAs). Circulating FFA represent about 60% of the total pool and are generated from the lipolysis of triglycerides (TG) in adipose tissue [217]. Finally, FFA from de novo lipogenesis (DNL) represent ~25% of the total pool and originate mainly from dietary carbohydrates metabolized in the hepatocyte.

In vitro studies show how FFA, according to their structure, can distinctively interact with components of the intestinal barrier and permeability such as TJs, bacteria, BA, and inflammation.

In Caco-2 cells the unsaturated LCFA eicosapentaenoic acid (EPA), docosahexaenoic acid (DHA), and γ-linolenic acid increase TJ permeability without disrupting the intestinal barrier [218,219]. In T84 cells, LCFAs decrease TJ permeability; EPA and DHA reduce the IL-4-mediated increase in paracellular permeability [220]. In Caco-2 cells, saturated MCFAs C8 (caprylate), C10 (caprate), and C12 (laurate) increased the paracellular permeability of the hydrophilic marker molecule [14C]-mannitol. The mechanism likely involves the activation of MLCK [221]. C10 (caprate) [222] and not lauric acid [221] is responsible for the conformational alteration of TJ proteins and the mechanism involves occludin and ZO-1. In the rodent model, the high-fat diet (HFD) increases gut permeability and changes are associated with decreased mRNA or protein expression of TJ (claudins-1-3, ZO-1) [223,224,225,226]. More specifically, in IL-10 knockout mice with IBD, IBD-like colitis is spontaneously triggered with an increase in gut permeability [227]. In another study, a diet enriched in saturated fat activated the Th1 immune response and this effect was associated with increased incidence of colitis [228]. A potential mechanism is that luminal taurine-conjugated BA can increase the density of *Bilophila wadsworthi*, a sulfite-reducing pathobiont. Further evidence about the effect of fat on gut permeability shows that the levels of proteobacteria in stool increase in mice on a high-fat and high-sugar combination diet, and changes are associated with increased faecal inflammatory markers [229]. An additional mechanism of damage can involve the levels of secretory IgA coating the gut microbiota, since the IgA levels are diminished in HFD fed mice [230].

Clinical trials show protective effects of higher consumption of DHA and progression of ulcerative colitis [231]. Opposite effects are generated by n-6 PUFAs (which promote inflammatory signaling pathways) and n-3 PUFAs (anti-inflammatory properties) [232,233,234]. There are no definitive results from clinical studies on the effects of a high-fat diet on the homeostasis of the gut barrier or on intestinal permeability. However, in healthy subjects, plasma LPS levels parallel the extent of fat intake [235,236]. In a group of healthy adults, serum endotoxin levels increase in the postprandial period following a high-saturated fat meal but decrease following ingestion of an n-3 (ω3) meal [237]. In another group of adult normal-weight males, no significant changes were recorded in gastroduodenal, small intestinal, or colonic permeability following fatty diet, although fasting endotoxin concentrations increased [238].

The role of LPS as a marker of increased gut permeability needs to be discussed. In the animal model increased serum levels of LPS point to increased gut permeability and LPS absorption from the leaky gut [226,236]. In addition, increased LPS absorption can modulate the lipidemia, since injection of LPS in mice reduced plasma HDL cholesterol and increased triglycerides [239]. In patients with T1DM high serum LPS level was associated with hypertriglyceridemia and diastolic hypertension [240]. The gut TLR4, a receptor for LPS, can influence the TJ function and activates signaling pathways leading to diet-induced insulin resistance and atherosclerosis [241]. In contrast, TLR4 knockout mice oppose the HFD-induced insulin resistance [242,243], and atherosclerosis [244].

In another study in mice, DSS-induced disruption of the intestinal barrier plus HFD induced downregulated ZO-1 and Claudin-1 expression in the colon (a picture compatible with leaky gut and likely LPS absorption), liver damage and inflammation with increased leukocyte infiltration, and mRNA expression of TLR4 and TLR9 inflammatory cytokines [245]. In humans, serum LPS levels increase with high fat and high carbohydrate intake and the mechanism involves increased TLR2 and TLR4 expression in mononuclear cells [246,247]. LPS-induced stimulation of TLR4 is associated with T helper cell (Th) 17 differentiation [248] and inflammatory changes via decreased expression of peroxisome proliferator-activated receptor-α [249].

Beside the above-mentioned studies, it is clear that a HFD has the potential to change the gut microbiome in animals [250,251,252] and humans [253,254]. The mechanism likely involves the interaction between bacteria and TJ.

In mice, a HFD induces insulin resistance, inflammation, and steatohepatitis while decreasing butyrate-producing bacteria *Butyricicoccus, Clostridium,* and *Turicibacter*, colonic butyrate levels, and expressions of occludin-1 and ZO-1. By contrast, serum LPS and mRNA expression of liver LPS-binding protein increase [255].

Of note, administration of *A. muciniphila* brings protective effects against HFD-diet in animal models. This effect is associated with the suppression of increased weight gain and lipid and glucose levels through a downregulation of intestinal TLR4 and TJ mRNAs [256]. 

In obese patients, a negative correlation has been detected between the relative abundance of *A. muciniphila* and fasting glucose levels, waist-to-hip ratio, and the diameter of subcutaneous fat cells. On the other hand, a direct relation has been shown with insulin sensitivity markers and other parameters after calorie restriction [53]. In patients with type 2 diabetes, reduced levels of faecal A. muciniphila extracellular vehicles (AmEVs) have been recorded, as compared with healthy controls [257]. Furthermore, a purified membrane protein from *A. muciniphila* is able to ameliorate glucose intolerance and decreases plasma LPS levels, with an upregulation of pathways involving insulin signaling and claudin-3 [258].

HFD, compared with low or normal fat diets, will increase the hepatic secretion of hepatic BA and the gut content of both primary and potentially harmful [259] secondary BA, especially in the colon [260]. BA contribute to the maintenance of the intestinal barrier, and mechanisms involve BA as signaling molecules on the small gut membrane receptor GPBAR-1 and the nuclear orphan receptor FXR [261]. GPBAR-1 knockout mice develop a disrupted molecular architecture of epithelial TJ. Expression of ZO-1 is increased, but subcellular distribution is deranged and associated with increased gut permeability. Mice become more sensitive to DSS stimuli and develop severe colitis [262]. By contrast, activation of GPBAR-1 ameliorates gut inflammation in models of chemically induced colitis. GPBAR-1 is involved in decreased mobility of blood monocytes to gut mucosa, decreased activation of macrophages, and decreased expression of inflammatory genes, including TNF-a, IFN-g, IL-1b, IL-6, and CCL2 [263].

FXR knockout mice show increased gut permeability [264]. FXR activation protects from chemically induced colitis and decreases gut permeability and inflammatory markers [265]. Notably, gut-specific FXR-deficient mice develop a combination of events, which include increased gut permeability, reduced mucosal integrity, decreased secretion of mucin 2 protein, and lower levels of E-cadherin protein [266].

### 4.7. Emulsifiers

Emulsifiers can damage the intestinal barrier via decreased microbial diversity and mucosal inflammation. These molecules appear in processed foods and beverages and the molecular coexistence of hydrophilic and lipophilic groups helps the dispersion of fat molecules and water-soluble molecules in a hydrophilic or hydrophobic environment, respectively [267]. As an example, carboxymethylcellulose decreases mucus pore size and significantly slows *E. coli* speed and particle diffusion rates through mucus. Tween (polysorbate 80) increases *E. coli* speed in mucus. Despite such distinct effects, both emulsifiers decrease the thickness of the mucus layer, resulting in closer contact of bacteria to enterocytes, changes to membrane-associated proteins such as ZO-1, and higher levels of bacterial translocation [268]. Activation of inflammatory pathways can occur with emulsifiers and involve Bcl-10, TLR-4, the release of NF-kB, and secretion of proinflammatory cytokines such as TNF-α and IL-6. Clinical studies have focused on the role of dietary manipulation and intake of emulsifiers, although intestinal permeability was not measured [269,270,271,272,273].

### 4.8. Alcohol

The intestinal barrier and intestinal permeability can change in response to both acute and chronic alcohol use [274]. Mechanisms include direct enterocyte damage and disruption of the tight junction [37]. Additional contributing aspects include impaired intestinal motility occurring upon acute [275] and chronic [276] alcohol intake.

In the Caco-2 cell culture, ethanol induces changes to the expression of ZO-1 and claudin-1, pointing to increased intestinal epithelial barrier permeability [277].

In the mice model, chronic alcohol administration induces intestinal dysbiosis, bacterial overgrowth and translocation [278].

Clinical studies support the effects of alcohol on the intestinal barrier. In healthy subjects, a single oral intake of alcohol equal to 1 g/kg was associated with altered histology in the duodenum [279]. Excessive alcohol intake was associated goblet cell depletion and mononuclear cell infiltration and inflammation in the rectal mucosa [280]. Direct intraduodenal instillation of alcohol (20 g) reduces the expression of ZO-1 and occludin and increases the phosphorylation of mitogen-activated protein (MAP) kinase isoforms. These findings are associated with increased the permeability of the small intestine and colon, measured by lactulose and sucralose excretion [281].

Patients with alcoholic liver disease exhibit increased miR-212 expression in colonic mucosal biopsies. This finding points to the miR-212-dependent inhibition of ZO-1 synthesis and derangement ZO-1 structure [282]. Intestinal dysbiosis can occur in alcoholics, as confirmed by a lower fungal diversity, and a shift of faecal mycobiome with an increased abundancy of the faecal *Candida* genus, as opposed to the *Penicillium* genus of non-alcoholic controls. These findings were not associated with increased intestinal permeability measured by plasma zonulin or LPS binding protein levels [283].

Intestinal permeability to large molecules is increased in chronic alcoholic men with liver disease. PEG 10,000 with a diameter of 46 Å is recovered in urines while plasma endotoxin LPS is increased, as compared to controls. PEG 4000 urinary recovery increases with and plasma endotoxins [284]. The ultimate value of such findings remains unclear. Large PEG molecules have a weight close to LPS (5000–8000 Da), but the chemical moiety of LPS, which combines a hydrophilic sugar chain with a hydrophobic lipid region, is prone to micellization in a watery environment with structures of 10^6^ Da. Thus, LPS permeability might not be totally reflected by PEG permeability.

Liver disease can be associated with increased intestinal permeability. We showed that patients with obesity/NAFLD had increased colonic permeability assessed by increased urinary recovery of sucralose [136]. In chronic alcoholics, however, alcohol abuse rather than liver damage accounts for increased intestinal permeability. In this respect, small intestinal permeability measured by lactulose/mannitol ratio was increased in alcoholics with chronic liver disease, but not in alcoholics without liver disease or patients with non-alcohol-related liver disease [285].

The effect of chronic alcohol abuse on increased intestinal permeability can persist beyond cessation of alcohol intake. This was the case with a study using ^51^Cr-labelled EDTA as a marker of intestinal permeability at baseline and 2 weeks of cessation of alcohol intake [286]. Alcohol use can affect chain fatty acids (SCFAs) and permeability occurring with circadian variation [180]. Further aspects of alcohol damage on the intestine are discussed in the context of NAFLD/bacterial products.

## 5. The Burden of Obesity

Human overweight and obesity are defined as an excessive accumulation of adipose tissue, a condition of adiposity. The World Health Organization defines overweight as a body mass index (BMI) of 25 to 29.9 kg/m^2^ and obesity as a BMI of ≥30 kg/m^2^ [287]. Both overweight and obesity are chronic diseases of increasing prevalence in children, adolescents, and adults as part of a global pandemic [288,289]. Both conditions put the populations at increased risk of many metabolic disorders such as hyperglycemia, insulin resistance, dyslipidemia, metabolic syndrome, and ultimately cardiovascular diseases [287,290,291,292] and many specific cancers [293]. Metabolic syndrome is a state of low-grade systemic inflammation which paves the way to the development of chronic diseases such as type 2 diabetes mellitus, NAFLD, and cardiovascular disease.

Obesity is the fifth leading cause of death in the world, and accounts for almost 3.4 million deaths yearly. By 2030, about 38% of the world’s adult population will be overweight and another 20% obese [294]. Data prevalence of obesity from the United States are very instructive and somewhat worrisome. The NHANES survey reported that from 1988 to 1994, 1999 to 2000, and 2017 to 2018 the age-adjusted overall prevalence of obesity increased progressively from 22.9 to 30.5 to 42.4% [295]. The prevalence of obesity was similar in adult males and females in 2017 to 2018 [296]. Globally, similar trends are following. Obesity worldwide in 2015 was reported in about 604 million adults and 108 million children [297]. Since 1980 the prevalence had doubled in more than 70 countries, while showing a continuous increasing trend in most other countries, similar between males and females in all age groups and highest during early adulthood. In 2015 the prevalence of obesity was higher for females than males at all socioeconomic levels and for all age groups. The most evident increase of obesity, ranging from 11.1 to 38.3%, occurred in males aged 25–29 from 1980 to 2015 and living in low- to middle-income countries.

Even the most severe types of obesity show continuous increasing trends, since the age-adjusted prevalence of class III obesity, meaning a BMI ≥ 40 kg/m^2^, increased from 5.7 to 9.2% between 2007 and 2018 [295,296]. The progressive expansion of visceral fat at increasing BMI, the chronic metabolically mediated pro-inflammatory status, and the ongoing insulin resistance condition are factors largely related to the increased cardiovascular risk and accumulation of excess free fatty acids/triglycerides and lipid metabolites in the liver [216,298,299,300,301,302]. 

Among all types of obesities, normal-weight but metabolically obese people and sarcopenic obese are the two categories at increased metabolic/cardiovascular/hepatic risk, due to a pro-inflammatory state affecting the visceral adipose tissue (VAT) as well as the liver [298].

## 6. The Burden of Non-Alcoholic Fatty Liver Disease (NAFLD)

Triglycerides physiologically accumulate in the hepatocyte as lipid droplets. Liver steatosis appears when the level of hepatic TG exceeds the 95th percentile that, in the case of healthy, lean individuals must be >55 mg per g of liver tissue. At histology, a fatty liver is characterized by the detection of intracellular triglycerides in 5% or more of hepatocytes [303,304]. Liver steatosis is also detectable by a magnetic resonance imaging proton density fat fraction [MRI-PDFF] when the estimated liver fat content is ≥5%, or by magnetic resonance spectroscopy, in presence of a liver fat content ≥5.56% [305].

The term NAFLD defines a type of liver steatosis which is not secondary to alcohol damage. NAFLD subjects do not drink or do not have significant alcohol consumption [306]), and there are no other causes which explain liver steatosis (Table 2). The alcohol-associated liver injury which includes steatosis remains the second most frequent aetiology.

The NAFLD spectrum is broad [320] and includes simple steatosis (nonalcoholic fatty liver, NAFL) and steatohepatitis (nonalcoholic steatohepatitis, NASH), liver cirrhosis and hepatocellular carcinoma (HCC). NAFL has little or no inflammation and no evidence of hepatocellular injury. NAFL occurs in about 80% of NAFLD subjects and is the non-progressive form since the risk of progression to liver cirrhosis is minimal [321]. NASH occurs in about 20% of NAFLD. Features of NASH include not only steatosis, but also inflammation and hepatocellular injury with ballooning and apoptosis (features which are indistinguishable from those of alcoholic steatohepatitis) [322]. NASH patients are at high risk of developing liver fibrosis [323,324,325,326] and to progress to cryptogenic compensated/decompensated cirrhosis as well as to HCC [327,328,329].

NAFLD is the most frequent liver disorder to date [303,330,331,332] with a median prevalence of about 25% worldwide [333,334,335]. The increasing trends of NAFLD likely depend on the increasing prevalence of other metabolic disorder worldwide, namely overweight, obesity, insulin resistance, type 2 diabetes mellitus, sedentary lifestyles, dyslipidaemia, and metabolic syndrome [297,333,336,337,338]. NAFLD occurs in non-obese individuals as well, encompassing the condition of “lean NAFLD” which develops with a prevalence of 10–30% in both Western and Eastern countries [339]. Lean NAFLD is also associated with metabolic dysfunction and increased cardiovascular risk [337,340]. The overall prevalence of NAFLD is likely underestimated since many studies rely on mild hypertransaminasemia and/or on ultrasonographic steatosis [333] and not on true fat content in the liver, as disclosed by exact quantitative evaluation. In particular, the specific serum alanine aminotransferase (ALT) may be normal in patients with NAFLD, while abdominal ultrasonography can easily detect a hyperechoic texture in the liver (“bright liver”) due to diffuse fatty infiltration. Ultrasonography is not able to distinguish the necro-inflammatory changes typical of steatohepatitis and has a poor accuracy in diagnosing the presence of a mild steatosis (<30%) [341]. NAFLD exposes the populations to the increased risk of liver-related mortality and, similarly to obesity, to all-cause-mortality due to increased risk of cardiovascular disease and extrahepatic malignancies [342,343,344]. The metabolic form of liver steatosis, NAFLD represents the leading liver disease worldwide with an estimated two billion individuals affected [345]. NAFLD is commonly associated with several metabolic abnormalities which include obesity, hypertension, dyslipidaemia, and diabetes [306,346].

Notably, liver fibrosis represents the strongest known predictor of poor clinical outcomes in NAFLD. Time for progression of fibrosis is significantly slower in NAFL than NASH with an average of 14 years vs. 7 years, and it is even shorter in NAFLD “rapid progressors” which account for 10–20% of patients [321]. To detect the predictors of rapid progression of NAFLD/NASH is therefore of paramount importance to reduce the ultimate burden of disease. Factors such as increased serum transaminases, especially alanine aminotransferase (ALT), morbid obesity, diabetes, genetic susceptibility with a family history of cirrhosis in first-degree relatives, and host microbiota, are likely involved [347,348,349,350]. In this respect, patients with cryptogenic cirrhosis exhibit 1.5–2% yearly incident risk of developing HCC. Therefore, patients with confirmed NASH require a careful screening [351] since NAFLD has become the second leading indication for liver transplantation in the US, and there are a growing number of cases with NASH-related HCC [334].

According to a recent debate, the term “non-alcoholic” seems to overemphasize “alcohol” rather than metabolic risk factors [306,346]. A change in terminology has been recently adopted from NAFLD to metabolic dysfunction-associated fatty liver disease (MAFLD). In this context the diagnosis of liver steatosis becomes active, rather than a diagnosis of exclusion, since MAFLD occurs with at least one of the following three comorbidities: overweight/obesity meaning expansion of visceral fat, type 2 diabetes mellitus, or evidence of metabolic dysregulation [352]. The term MAFLD is gaining interest worldwide [353,354,355,356,357,358], despite some authors warning that changing definition requires further knowledge about the molecular basis of the disease entity, new insights in risk stratification, and practical implications [335]. Indeed, studies are in progress looking at the contribution of environment, comorbidities, and the gut microbiome to the pathogenesis and natural history of NAFLD/MAFLD [11,136,216,359].

## 7. The Intestinal Barrier: General Implications in Obesity and NAFLD

Obesity and NAFLD develop because of the interaction of genetic factors, epistasis, and environmental factors, exposome [349,360] such as race, ethnicity, gender, age, and foetal period. Lifestyles which include quality of diet, sedentary life, sweetened beverages, hypercaloric intake, induce potent gut, microbial, and dietary modifications [3,361]. Additional factors playing a key role are food contaminants, contaminated consumer products, or air pollution [362,363,364,365,366,367,368,369]. Diet is a main contributor to gut microbiota diversity and accounts for more than 55% of the variations, compared to about 12% estimated for genetic variation [370]. Dietary habits will shape the microbiota composition already during breast and formula feeding. *Bifidobacteria* spp. are higher in breast-fed babies compared to formula-fed babies [371,372], while formula-fed babies show higher levels of *Bacteroides* spp. and *Lactobacillus* spp. [373]. Probiotics comprise non-pathogenic microorganisms used as food ingredients which bring benefits to the health of the host. *Limosilactobacillus reuteri* can significantly lower the low-density lipoprotein cholesterol (LDL-C) in patients with hypercholesterolemia, likely involving the orphan FXR [374]. Prebiotics are fermented dietary fibers able to modulate the composition and/or activity of colonic microbiota with beneficial effects on health [375]. Examples of prebiotics are lactulose, resistant starch, and inulin (targeting especially *Bifidobacterium* and *Lactobacillus* genera) [376]. Animal studies have addressed additional issues [377]. Humanized mice generated by transplanting human faeces into germ-free mice were switched from a low-fat, plant polysaccharide-rich diet to a “Western diet”. This included a high fat and sugar content which altered the composition of the microbiota within a single day with an increased number of the Erysipelotrichi class of bacteria within the Firmicutes phylum and reduced *Bacteroides* spp. Mice fed a vegetarian fiber-rich diet had lower counts of *Bacteroides* spp. *E. Coli* and other bacteria compared to the controls.

In general, the vegetarian diet is associated with decreased *Bacteroides* spp., *Bifidobacterium* spp., *E. coli* and Enterobacteriaceae spp. [378], decreased Enterobacteriaceae and increased *Bacteroides* [379], increased Bacteroidetes, and decreased Firmicutes and Enterobacteriaceae [380]. By contrast, a high-fat diet is associated with decreased genera within the ileal class *Clostridia*, while colonic *Bacteroidales* increase [381]. Others reported increased *Lactobacillus* spp., *Bifidobacterium* spp., *Bacteroides* spp., and *Enterococcus* spp., and decreased *Clostridium leptum* and *Enterobacter* spp. [382]; increased Firmicutes to Bacteriodetes ratio and increased *Enterobecteriaceae* [383]; and increased *Bacteroidales*, *Clostridiales* and *Enterobacteriales* [223]. The calorie-restricted diet can decrease the Firmicutes to Bacteroidetes ratio [384].

External agents can strongly influence the intestinal barrier function by interacting with the microbiota [44], the gut immune system [385], and the ability to secrete local peptides and immunoglobulins with antimicrobial function [18,386,387].

Beside dietary habits [388] many other factors are involved, such as smoking [389], ethanol intake [390], drugs (antibiotics [188,391,392,393,394], liraglutide [395], metformin [396], curcumin [397]), and environmental pollutants (heavy metals, persistent organic pollutants, volatile organic compounds, and pesticides [398,399,400,401,402]).

Changes to gut function and permeability can have potent consequences on metabolic homeostasis, including energy harvest and fat accumulation in obesity and NAFLD [403]. The role of dietary habits deserves attention in this respect, due to the potential repercussions on all levels of the intestinal barrier [404,405]. Whether the starting problem is the initial modification of the gut microbiota pointing to dysbiosis, rather than the composition of diet per se*,* is a matter of debate [113]. A Western-style fat- and sugar-enriched diet might initiate the detrimental process. High-fat or fiber-deprived diets in mice can reshape the gut microbiota, decrease the thickness of the mucous layer, and increase gut permeability, with low-grade gut inflammation [46,406,407]. High-fat or high-fructose intake increases gut permeability in the animal models of NAFLD [408,409]. Saturated fat and fructose in diet promote the pro-inflammatory microbiome, decrease the production of protective SCFA, and increase the recruitment of macrophages producing cytokines and TNF-α as markers of gut mucosal inflammation [410,411]. Dietary changes can decrease the expression of TJ proteins and increase the pathological permeability of the intestinal barrier [412]. Indeed, a high-fat diet or a high-fructose diet in the animal model induces changes of gut ZO-1 and occludin and promotes endotoxemia [120,413,414,415]. A diet-dependent increase of serum LPS will promote TLR-mediated low-grade liver inflammation. This is a metabolic scenario potentially associated with obesity, NAFLD, and NASH [405].

Thus, one chain of events can include dysbiosis, changes of TJ proteins [113], reduced lamina propria Treg cells, increased production of IFN-γ (by Th1 and CD8+ T cells), and increased production of IL-17 (by γδ-T cells) as expressions of local inflammatory changes [407]. Several findings are available in both animal models and human studies.

Mice on a high-fructose, cholesterol diet have defective gut permeability and develop more severe NASH than control mice, while colon tissues from NAFLD patients display lower levels of the JAM-A junctional adhesion molecule and higher levels of inflammation than subjects without NAFLD [416]. Mice genetically deficient in Jam1 on a high-fat and high-fructose diet had increased gut permeability, endotoxemia, and hepatic inflammation [417]. In NAFLD mice, administration of probiotics for 4 weeks improved NKF-beta activity, steatosis, and hepatomegaly [418]. In the genetically obese (ob/ob) mice fed with a high-fat diet, the antidiabetic drug liraglutide modified the overall composition and the relative abundance of gut microbiota phylotypes involved in the pathogenesis of NAFLD with reduced Proteobacteria, increased *A. muciniphila*, and decreased liver fat content reversing steatosis [395]. In mice, curcumin changed the composition of several operational taxonomic units related to hepatic steatosis. The treatment attenuated liver fat deposition and improved the integrity of the intestinal barrier [397]. Metformin administration in mice of the inbred strain C57Bl/6J that were fed fat-, fructose-, and cholesterol-rich diets showed protective effects in both gut microbiota and integrity of the intestinal barrier [396].

Adolescents with NAFLD observed for 24 h had increased postprandial endotoxin levels following fructose-enriched but not glucose-enriched drinks [419]. By contrast, a less pro-inflammatory diet, such as the Mediterranean diet, enriched in fibers, mono- and polyunsaturated fatty acids, antioxidants, polyphenols, and phytochemicals, could protect gut permeability by increasing SCFAs-producing bacteria because of diet-induced prebiotic effects [420]. Nevertheless, gut permeability did not improve in NAFLD patients with increased baseline gut permeability by ^51^Cr-EDTA, put on 16 weeks of a Mediterranean diet and 16 weeks of a low-fat diet [421].

Antibiotics can greatly affect the microbiome with consequences on energy storage and metabolic disorders which include diabetes, obesity, fatty liver, and metabolic syndrome [188,391,392,393,394]. The use of polymyxin B improved steatosis grades in both rats and humans on total parenteral nutrition and in alcohol-exposed rats [422,423,424]. Following gut bypass surgery with associated hepatic steatosis, metronidazole improved the hepatic damage [425]. The administration of some probiotics in children increased levels of glucagon-like peptide-1 (GLP-1) and showed improvement in fatty liver [426].

## 8. Intestinal Barrier Features in Obesity

Obesity and associated metabolic abnormalities can induce changes of networking, bidirectional crosstalk, and control of inflammation at the level of different tissues, such as visceral fat and the liver, and will pave the way to initiation, perpetuation, and aggravation of metabolic damages [5,11,403]. The gut microbiota plays an important role in this respect and might represent a link with obesity [235] since the microbiota participates in the regulation of fat storage [427]. Exposure to antibiotics can decrease bacterial diversity and put individuals at risk of weight gain [428], especially during the first 6 months of age [429]. By contrast, probiotics, similarly to antibodies to TNF, inhibit inflammatory activity and improve NAFLD [418]. The gut microbiota can increase energy production from diet, promote low-grade inflammation, and govern fatty acid tissue composition [186,430]. There are no definitive conclusions in this respect, due to the complexity and diversity of gut microbes, ethnic differences in the populations, and large variations between studies [431].

Studies point to the changed ratio between Firmicutes phyla which increase in obesity and Bacteroidetes phyla which decrease in obesity. This diversity can be associated with increased energy absorption from food and increased low-grade inflammation [432,433]. Morbidly obese patients undergoing bariatric surgery by Rouex-en-Y gastric bypass exhibit a dramatic amelioration of their metabolic profile, with a concomitant shift of bacterial population [13,434,435,436]. Of note, mice experiments showed that faecal transplantation from RYGB-treated mice into germ-free mice led to weight loss and decreased fat mass in mice [437].

Other factors can shape the metabolic profile in obesity. In the obese subjects, a fiber-poor diet will affect the gut environment, microbiota diversity, and the fiber-derived production of SCFA, as reported before [29,143]. The role of SCFA in obesity cannot be ignored. Butyrate dietary supplementation reduces diet-induced insulin resistance in mice. The mechanism likely includes increased energy expenditure and mitochondria function [438], while a fat-enriched diet can easily shift the composition of the gut mucus layer [48] and associated microbiota. In addition, by re-shaping the diversity of the gut microbiome, obesity can also have a detrimental effect on the microbiome-immune system crosstalk, the gut immune response, and the production of bacteria-specific IgA antibodies [439,440]. In this context, a dysbiotic microbiome is a predisposing factor linking the immune system with obesity-associated disease outcomes [91,441]. The intestine develops alterations in immune composition during obesity and function which includes the microbiota. A dysbiotic microbiome is an important factor linking the immune system with obesity-associated disease outcomes [441].

Such changes will have a consequence on the performance of the intestinal barrier and the associated components. A subsequent step is the permeation of bacterial products in the portal tract to the liver [113]. Notably, microbially produced endotoxins such as LPS can be taken up into chylomicrons that are formed from dietary saturated fats. This step can promote inflammation in the host that induces insulin resistance [29]. Such changes, in turn, will impact the mechanisms involved in both local and systemic inflammation controlling metabolic abnormalities.

Genetically obese ob/ob mice and obese subjects have increased levels of SCFA in the cecum and faeces, likely due to decreased colonic absorption [442,443,444]. Butyrate and propionate protected from diet-induced obesity [445]. Acetate given orally also improved glucose tolerance [446]. SCFA-dependent activation of the AMP-activated protein kinase (AMPK) in liver and muscle tissues can activate pathways involved in cholesterol, lipid, and glucose metabolism. Mechanisms include interaction with peroxisome proliferator-activated receptor-gamma coactivator 1 alpha (PGC-1α), peroxisome proliferator-activated receptor gamma (PPARγ), and Liver X receptors (LXR) [447]. The interaction of acetate, propionate and butyrate with G-protein-coupled receptors (GPR41 and GPR43), resulting in the release of the hormone glucagon-like peptide-1 and PYY has profound metabolic consequences [448,449]. Gpr41 is expressed in intestine, adipocytes, and immune cells, suggesting involvement in lipid and immune regulation. Of note, Gpr41-KO mice on a high-fat diet had lower body fat mass, increased lean body mass, improved glucose control and lower HOMA index, indicating improved insulin sensitivity. These animals had higher energy expenditure accompanied by higher core body temperature and increased food intake, decreased liver weight and content of triglycerides and plasma levels of cholesterol. Gpr41-KO mice had decreased lipid interspersed in brown adipose tissue with no differences in white adipose tissue (WAT) cell size but significantly lower macrophage content. Thus, the absence of the GPR41 receptor protects from high-fat diet-induced obesity and dyslipidemia at least partly via increased energy expenditure [450].

The same outcome did not occur in mice grown under germ-free conditions or when treated with antibiotics [451]. A second receptor of SCFA, GPR41, is activated mainly by propionate and butyrate [445]. Beside inducing the gut hormone peptide YY (PYY) and GLP-1, this receptor improves insulin signaling through SCFA produced by gut microbiota [452,453].

Dysregulation of BA homeostasis during the enterohepatic circulation might also play a role by up-regulating transcription factors that link it to nutritional-induced inflammation, lipid absorption, and de novo lipogenesis [454].

Toll-like receptors (TLRs), the nuclear factor kappa (NF-kB) are master regulators of inflammatory pathways, and their role is important in obesity [455,456,457,458]. 

LPS (originating from the outer membrane of Gram-negative bacteria) are produced in the gut [455].

Upon increased fat intake, levels of LPS increase. Animal experiments in mice support this possibility and adding LPS to a normal diet induce insulin-resistance and lead to weight gain. LPS binding to the TLR4 receptor on macrophages activates the production of inflammatory markers. In turn, this step induces insulin resistance which is secondary to the inhibition of pancreatic β-cell function and decreased gene expression of Pancreatic And Duodenal Homeobox 1 (PDX1) [459].

### Obesity and Gut Immunity

Mice models of diet-induced obesity confirm that there is a shift in the inflammatory potential of the gut immune environment. Such adaptation increases the numbers of lamina propria Th1 and CD8+ T cells, CD44+ MAIT cells, gut homing CCR2+ macrophages, and gut intra-epithelial CD8αβ+ T cells. In parallel, tolerogenic cell types will decrease. Small intestine type 2 innate lymphoid cells (ILC2s) promote obesity via an IL-2 feedback system. Obese subjects show increased gut CD8αβ+ T cells. Overall, dietary obesity-driven cellular immune changes will contribute to the onset of an inflammatory environment and gut dysfunction and will have consequences on deranged glucose homeostasis [91]. The innate immune system is responsive to the obesogenic environment as documented by a decrease in IL-22- and IL-17-secreting gut group 3 innate lymphoid cells (ILC3s) [460].

The obesity-induced pro-inflammatory skewing of immune cells is a predisposing factor to the release of cytokines (TNF and IFNγ), which parallels a reduction in protective cytokines (IL-10 and IL-22). This will result in the reduced expression of antimicrobial proteins (RegIIIγ), mucin, and epithelial tight junction proteins [407,461]. Barrier dysfunction can pave the way to the onset and aggravation of chronic inflammation, metabolic syndrome, and insulin resistance [235].

Dysfunctional gut immune cells can promote the impaired bioavailability of gastrointestinal hormones such as GLP-1, which has important metabolic effects (as incretin that enhances the secretion of insulin to reduce blood glucose levels) and, in turn, is involved in the maintenance of barrier integrity acting on TJs and gut intraepithelial lymphocytes, which express the glucagon-like peptide 1 receptor (GLP1R) and have anti-inflammatory activity cytokines [462]. Additional mechanisms driven by obesity include gut epithelial lymphocytes sequestering GLP-1, decreased availability of GLP1-secreting enteroendocrine L cells, or lymphocyte-driven upregulation of the expression of molecules (dipeptidyl peptidase 4 (DPP4)) degrading GLP1 [91,463]. Lastly, gut immune cells such as anti-inflammatory IgA+ antibody-secreting cells (ASCs) can leave the barrier, decreasing the local protection (IL-10 and IgA), and migrate to other inflamed tissues (liver, visceral fat) [439].

An additional aspect includes the consequences of immune-microbiota crosstalk as a regulator of metabolic alterations. Obesity is associated with dysbiosis and a loss in bacterial diversity. This condition impairs the capacity of mononuclear phagocytes to produce factors effective on IgA class switching (transforming growth factor-beta TGF-β), interleukin-5 (IL-5), retinoic acid (RA) via retinaldehyde dehydrogenase (RALDH) enzymes, and a proliferation-inducing ligand (APRIL)). Both quantity and quality of secretory IgA (SIgA) will be affected with dysfunctional capacity to bind bacteria and providing the condition for expansion of opportunistic and pathogenic taxa Proteobacteria and associated dysbiosis. In addition, the environment will produce defective secretion of factors linked to IgA antibody-secreting cell (ASC) function, and reduced ability to induce T helper 17 (Th17) and regulatory T (Treg) cells. In parallel, pro-inflammatory signaling in gut epithelial cells, T cells, and potentially enteric neurons, will impair the production of anti-microbial peptides (AMPs), or can blunt the protective effects of *Akkermansia*. A further aspect to consider is the microbiota ability to control the immune cells, a mechanism which can fail during obesity, and involve short-chain fatty acids (SCFAs) function (via G protein-coupled receptors (GPRs)) to increase levels of gut secretory IgA, promote Treg cell responses and strengthen the CX3CR1+ MNP function. Aryl hydrocarbon receptor (AhR) ligands appear to increase levels of protective interleukin (IL)-22 and IL-10 cytokine, AMP production, and the promotion of epithelial layer mucus and tight junction proteins. BA will act on the ileal farnesoid X receptor (FXR) and GPBAR-1, while increasing the population of Treg cells and decreasing Th17 cells. Mechanisms likely change during diet-induced obesity [91].

As the prevalence of overweight and obesity are rising worldwide, the impact of gut microbiota on the development of type 2 diabetes mellitus is becoming clearer [464]. Mechanisms involve modifications in the secretion butyrate and incretins [413,434,453,465,466]. Evidence shows that type 2 diabetes mellitus patients host gut dysbiosis, decreased butyrate-producing bacteria, and increased opportunistic pathogens [188]. Involvement of pathways such as insulin signaling, inflammation, and glucose homeostasis is an additional possibility [188,434,451,467,468,469,470,471]. The gut microbiota can modulate the SCFA binding to GPR41 and influence the secretion of GLP-2 and PYY, two key insulin-signaling molecules [413], which decrease insulin resistance and β-cells function [413]. In mice, an increase in *Bifidobacterium* spp. has an anti-inflammatory effect via production of GLP2 and reduced gut permeability [413].

## 9. Intestinal Barrier Features in NAFLD

Metabolic abnormalities and gut microbiota can heavily affect the liver which is central in gluco-lipidic homeostasis. Evidence from pre-clinical studies indicates that germ-free mice might be protected against obesity and hepatic steatosis [472], although results are still controversial [473]. Furthermore, a specific gut microbiome signature has been detected in NAFLD [474]. Knowing the pathophysiology of the gut–liver axis is very instructive in this context. The microbiome provides a huge source of diverse bacterial products and metabolites. If gut permeability increases abnormally, the epithelial barrier is massively crossed by bacterial products (mainly lipopolysaccharides, peptidoglycans, nucleic acids, flagellin, trimethylamine, ethanol and other volatile organoids, fatty acids, acetaldehyde), with release of P/MAMP in portal blood. This process also induces the release of pro-inflammatory cytokines, eicosanoids, and chemokines from lymphatic cells. As a limiting factor, the gut vascular barrier parallels the epithelial barrier, regulates the rate of transfer into bloodstream, and filters the molecules entering the portal blood, also according to their size. When arrived into the liver, these molecules drive chronic inflammation, fibrogenesis, and carcinogenesis [475].

### 9.1. NAFLD as a Model of Systemic Inflammation

Low-grade inflammation driven by the chronic release of cytokines, acute phase proteins, and adhesion molecules is a feature of NAFLD and especially NASH patients [476,477,478,479]. Innate immunity is involved [480,481] and the gut microbiota provides inflammatory mediators and metabolites as well [350].

Low-grade chronic inflammation contributes to the onset and progression of metabolically driven hepatic diseases from NAFL to NASH to cirrhosis [482,483] and extrahepatic diseases which include atherosclerosis and cardiovascular complications [320]. Pathways involved include insulin signaling and insulin resistance [484]. Distinct cytokines play either a pro- or anti-inflammatory role. Interleukin 37 (IL-37) protects from metabolic dysfunction as confirmed after bariatric surgery (associated with an increase in subcutaneous adipose tissue IL-37 [485]). In transgenic IL-37 (IL-37tg) mice metabolic dysfunction and insulin sensitivity improved in various models of obesity-related disorders [486].

### 9.2. The Gut Microbiota

The gut microbiota can be involved in the pathogenesis of NAFLD, although a direct effect is still a matter of research due to the complex combination of local and systemic factors [403]. The BMI contributes to shape the gut microbiota [487] and this is pathophysiologically relevant, since NAFLD is very often associated with overweight and obesity [11,136,488]. Obesity, metabolic, and liver diseases can develop on the top of anatomical and functional changes of the intestinal barrier [408,489]. The concept of the intestinal barrier includes the immune barrier, the gut vascular barrier, and the liver barrier with most of the blood flow moving from the intestine to the liver through the portal vein. Immune adaptations at the gut level can disrupt gut permeability promoting bacterial translocation. In addition, specific profiles of the gut microbiome and ongoing dysbiosis can contribute to the inflammatory and fibrosis responses in NAFLD patients [490].

Few pre-clinical studies are worth mentioning in this context.

Germ-free mice receiving microbiota from animals fed a high-fat diet and developing weight gain, hyperglycaemia, and a high plasma concentration of pro-inflammatory cytokines, developed the same features, together with hepatic macrovesicular steatosis. Findings from this study indicate that gut microbiota can promote NAFLD independently from obesity [491]. 

Manipulation of gut microbiota can re-shape the bacterial metabolic signature and function. In the high-fat animal model guar gum changed the gut microbiota composition and decreased diet-induced obesity. Glucose tolerance improved, despite the liver developing more inflammation and fibrosis [492]. A pro-inflammatory role for bacterial-derived secondary BA was important in this context, since chronic oral administration of an antibiotic suppressed gut bacteria, reduced portal secondary BA levels, and decreased liver damage.

Notably, rats fed a HFD and high glucose/fructose syrup, abbreviated as HFGFD, exhibited NASH, including portal hypertension, which improved after faecal transplantation from a healthy rat [493].

Maternal obesity increases the risk for offspring obesity and NAFLD and can disrupt mechanisms of microbial immunity and metabolic function in the infant. Sodeborg et al. [494] performed an animal study in which germ-free mice were colonized with stool microbes from 2-week-old infants born to obese or normal-weight mothers. Mice receiving stools from infants of obese mothers had many abnormalities, including increased hepatic gene expression for endoplasmic reticulum stress and innate immunity, histological periportal inflammation mimicking NAFLD changes, gut permeability increased, and impaired macrophage function was documented by reduced macrophage phagocytosis, and dampened cytokine production. A Western-style diet in these mice was associated with excess weight gain and accelerated NAFLD.

Clinical studies confirm that gut dysbiosis occurs in NAFLD. In addition, NASH likely brings a peculiar “microbiome signature” promoting disease progression and clinical phenotype.

Wigg et al. [495] used 14C-D-xylose and lactulose breath test and observed that NASH subjects (N = 22) had small gut bacterial overgrowth and increased circulating endotoxin and TNFa levels, compared to control subjects (N = 23). A metanalysis on 128 NAFLD patients and 83 control subjects confirmed that the gut permeability was abnormal in liver steatosis, and more evident in NASH patients [496].

Sung et al. described the case of small gut bacterial overgrowth following jejuno-colic bypass surgery. Dysbiosis was reversed after surgical correction [497].

The development of NASH is often associate with small intestinal bacterial overgrowth, a condition characterized by increased expression of TLR4 on CD14 positive monocytes and higher plasma IL-8 levels [498].

Several reports have underscored the peculiar signature of gut microbiome in NAFLD/NASH patients when looking at phylum, family, genus, and species [499,500]. Particularly enriched in NAFLD was the phylum *Proteobacteria* [474], the family *Lactobacillaceae* [501], the genus *Bacteroides* [490], *Ruminococcus* [490], *Lactobacillus* [501], and the species *E. coli* [474]. By contrast, decreased in NAFLD was the phylum Actinobacteria [502], Bacteroidetes [502], Firmicutes [474], the genus *Oscillobacter* [502], *Prevotella* [490], *Ruminococcus* [501], *Coprococcus* [501], and the species *Faecalibacterium prausnitzii* [501].

Significant differences between obese and NASH patients emerged at phylum level for Proteobacteria, at family level for Enterobacteriaceae, and *Escherichia* genus [499].

Mouzaki et al. [500] found that NASH had a lower rate of *Bacteroidetes* compared to steatosis and healthy controls. Species within the *Oscillobacter* genus were lower in NAFLD, whereas *Ruminococcus*, *Blautia*, and *Dorea* were increased in NASH [502].

Notably, *Bacteroides* abundance increased and correlated with histology-proven NAFLD severity, whereas *Prevotella* abundance was decreased. In addition, *Ruminococcus* abundance increased in more severe diseases, especially in advanced fibrosis. [490].

A recent observation confirmed that a circulating microbiome is detectable in blood, as found in central, hepatic, and portal venous blood and peripheral blood from seven liver cirrhosis patients receiving a transjugular portosystemic shunt. Cases showed how dominating *Proteobacteria* and changes were compartment-specific. Detected bacteria can be viable and potentially bioactive. There was a direct correlation with cytokine levels suggesting that inflammatory changes in liver cirrhosis are an expression of gut-derived bacteria [503].

Another study detected changes in blood microbiota associated with liver fibrosis in obese. Although the faecal microbiome was not studied, the analysis of blood microbiota might soon become a potential biomarker for the detection of liver fibrosis in patients at risk [504].

In the study by Loomba et al. [474] on biopsy-proven NAFLD, advanced fibrosis was characterized by an increased abundance of Proteobacteria and *E. coli* and a decrease in Firmicutes. Thus, microbiome markers would point to advanced fibrosis in NAFLD.

NAFLD cirrhosis was differentiated when including a panel of 27 bacteria [505].

Gut dysbiosis in NAFLD can develop irrespective of obesity or insulin resistance since *Lactobacillus* and Lactobacillaceae were more abundant and *Ruminococcus*, *F. prausnitzii*, and *Coprococcus* were decreased compared to healthy controls [501]. Data on *Ruminococcus* require further studies since others have reported increases in NAFLD [490,502]. Notably, probiotic treatment might become a strategy to decrease liver fat in NAFLD. In a placebo-controlled trial of 12 weeks, treatment with *Lactobacillus acidophilus, Lacticaseibacillus rhamnosus, Lacticaseibacillus paracasei, Pediococcus pentosaceus*, *Bifidobacterium lactis*, and *Levilactobacillus brevis* decreased body weight, total body fat, and intrahepatic fat in obese patients [506]. Certain bacteria with rather pro-inflammatory features increase and include Proteobacteria or *E. coli,* while protective bacteria are decreased and include *F. prausnitzii*. Bacterial metabolites and microbiota-generated secondary BA contribute to NAFLD-associated metabolic dysfunction.

### 9.3. Bacterial Products

#### 9.3.1. MAMPs/PAMPs

The role of bacterial products/metabolites in NAFLD is a matter of ongoing research. In the human body, the gut microbiota is a dynamic source of circulating metabolites which have several protective, anti-inflammatory, or proinflammatory functions. M/PAMPs are produced by the interaction of the microbiota with endogenous and exogenous substances, and include gases, metabolites, and bacterial products.

Key targets of microbial metabolites are the G protein-coupled receptors (GPCRs) [507] which appear in many cell types and have immune and metabolic functions [508,509]. Permeation of PAMPs and MAMPs such as LPS, microbial DNA, peptidoglycans and lipopeptides, metabolites, and whole bacteria can bring these products to local MLNs. Here, the clearance can be defective [510,511,512,513], and this defect contributes to the migration of the agents/bacteria via the mesenteric and portal circulation to the liver [2]. In the liver, the detrimental agents can perpetuate the local damage via activated Kupffer cells [124,514,515,516,517]. A wider systemic low-grade chronic inflammatory response is also possible [515,518,519,520,521].

PAMPs and TLRs interaction can activate specific intracellular molecular pathways which are MyD88-dependent or MyD88-independent. This step is followed by the activation of NF-κB and several inflammatory cytokines which include TNF-α, IL-1β, IL-6, IL-12, IL-18, chemokines such as CXCL1, CXCL2, CCL2, CCL5, CCL3, CCL4, and vasoactive factors such as nitric oxide (NO) [522]. The recruitment of systemic leukocytes, namely CD4+ T cells, neutrophils, and monocytes will promote inflammatory changes [514,515], hepatocyte apoptosis and necrosis [523]. Activated and proliferating hepatic stellate cells (HSC) will release transforming growth factor-β (TGFβ) which plays a role in liver fibrosis [517,524]. Upregulation of the expression of matrix metalloproteinases (MMPs) will promote the destruction of hepatic tissue [525,526], and this step is associated with the increased expression of TIMPs (tissue inhibitors of matrix metalloproteinases). TIMPs inhibit the degradation of hepatic collagen fibrogenesis [525,526,527,528] and become predictive markers of NASH, such as TIMP-1 [529]. The production of reactive oxygen species (ROS) following the ongoing oxidative stress contributes to liver damage [522,530], since hepatocytes become sensitive to oxidative stress-related molecules [530,531,532]. The damage also includes liver steatosis [533], and disruption of the intestinal barrier with negative effect on the gut–liver axis. The abnormal gut redox state can be triggered by factors such as diet [534], alcohol [535], infections [536], primary inflammatory diseases [537], and drugs [538]. The onset of hypoperfusion-dependent hypoxia in the gut mucosa can increase the activity of xanthine oxidase, ROS release, and therefore oxidative damage [539]. The ROS-induced activation of the TLR of Kupffer cells will also generate ROS [540], cytokines and chemokines, and the activation/proliferation of hepatic stellate cells (HSC) [532,541]. In this complex inflammatory scenario, protective mechanisms at the gut mucosa include the release of macrophagic IL-10, which contributes to the modulation of the innate immune activation, decreased tissue damage, amelioration of integrity of the intestinal barrier, and decreased endotoxin absorption [542,543]. A similar effect in the liver contributes to reduced inflammations and fibrosis, and decreased activation of Kupffer cell functions [544,545]. In addition, NK cells become killers of early activated and senescent HSCs, a step leading to the limitation of fibrogenesis [546,547].

If the intestinal barrier becomes pathologically permeable, several metabolites will enter the portal circulation becoming effectors of liver damage and inflammatory changes by acting on patter-recognition receptors (PRRs) located on the hepatic stellate cells [548] and the macrophagic Kupffer cells [517]. Endotoxins will interact with liver TLR4, TLR9 by methylated DNA and TLR2 by Gram-positive bacteria [84], and this step is the first step of innate immune response. Activated hepatic stellate cells promote fibrosis via TLR4 signaling that downregulates BMP and activin membrane-bound inhibitor homologue defined as BAMBI, a decoy receptor for transforming growth factor-β (TGFβ) [522]. Additional inflammatory events include the activation of nuclear factor- χB (NF-χB) by the myeloid differentiation primary response protein (MYD88) and enhanced expression of hepatic tumour-necrosis factor (TNF)-alpha. These responses contribute to NASH progression [392]. Contributing factors of liver damage include the release of inflammatory cytokines, oxidative stress, and endoplasmic reticulum stress [549]. Animal studies show that diets enriched in fat or deficient in choline drive steatogenic, inflammatory, and fibrogenic responses via TLR-4 or TLR-9 [550,551,552].

The combination of dysbiosis (meaning changes of quality and/or quantity and/or topographic distribution of the microbiota) and increased gut permeability promotes the release of MAMPs and PAMPs, such as LPS, or by products of their metabolism such as ethanol, SCFAs, and trimethylamine, which are transported through the portal vein. This flow contributes to about 70% of the blood entering the liver, and the transport of toxic molecules can ultimately cause liver damage [181,416,553]. LPS will activate the cell toll-like receptor 4 of both Kupffer cells and hepatic stellate cells. Kupffer cells mainly reside in the lumen of hepatic sinusoids as 80% to 90% of colonized macrophages in the human body. These phagocytic cells play a crucial role in regulating and maintaining homeostasis and upon liver damage and activation will release inflammatory cytokines and chemokines [554].

In animal studies, rats injected with LPS develop steatohepatitis, while anti-tumour necrosis factor antibodies improve the steatosis [555,556]. Genetically obese mice show increased gut permeability which promotes the increased portal endotoxemia [120,557]. Obesity-induced leptin is involved in NASH progression [558] via enhanced responsivity to bacteria-derived endotoxins.

Mice receiving a high-fat diet (HFD) develop steatosis and accelerated NASH progression with liver inflammation and fibrosis and upregulation of CD14 in Kupffer cells and hyperreactivity against low-dose LPS. Chow-fed control mice did not have a similar effect and leptin increased hepatic expression of CD14 via STAT3 signaling and hyperreactivity against low-dose LPS without steatosis, while leptin-deficient ob/ob mice with severe steatosis had a marked decrease in hepatic CD14 [558].

Studies in mice investigated the role of innate immunity inflammasomes NLRP6 and NLRP3, protein IL-18 in relation to gut microbiota and NAFLD/NASH progression [392]. The gut microbiota profile changed with the inflammasome deficiency while hepatic steatosis and inflammation worsened. TLR4 and TLR9 agonists migrated into the portal circulation and this influx increases hepatic tumour-necrosis factor (TNF)-alpha expression and facilitates the progression of NASH. The findings suggest that upon defective NLRP3 and NLRP6 inflammasome sensing, the microbiome will connect changes related to systemic auto-inflammatory and metabolic disorders. An association exists between NAFLD, small intestinal bacterial overgrowth, and increased endotoxemia [235,495,559,560] when gut dysbiosis becomes small intestinal bacterial overgrowth [561,562]. Indeed, the prevalence of small intestinal bacterial overgrowth was higher in NAFLD [559]/NASH [495] patients than healthy controls.

The role of specific bacterial species must be considered as well in patients with liver damage. The gut microbiota plays a role in the evolution from NAFLD to NASH. In NAFLD, disease-specific gut microbiome signature exists with pro-inflammatory bacteria present. Protective bacteria are decreased, and the gut microbiota is involved in this process [418]. A gut microbiota signature exists in obesity and NAFLD [433,474,505]. This imbalance causes the dismission of many bacterial metabolites, the generation of bacteria-dependent secondary BA involved in NAFLD metabolic dysfunction. [472].

In NAFLD with advanced fibrosis, patients show increased abundance of *E. coli* and *Bacteriodes vulgatus* [474]. NASH obese children have greater abundance of the genus *Escherichia* [499]. Dietary factors can re-shape the gut microbiota in NAFLD since a Western diet enriched in fat, proteins of animal origin and simple sugars will increase the abundance of *Bacteroides*. A diet rich in fiber and indigestible plant polysaccharides will increase *Prevotella* abundance. This shift of microbiota is of interest since *Bacteroides* genus correlates with NASH while *Prevotella* abundance decreased in NASH [490,563]. In humans, *Ruminococcus* genus abundance increases with significant liver fibrosis meaning a score ≥F2 [490] and in the animal model *Ruminococcus* genus correlates with the development of metabolic impairment [564].

Changes of gut microbiota also occur in conditions related to NAFLD which represents the hepatic expression of the metabolic syndrome [565]. Obese individuals display increased Firmicutes/Bacteroidetes ratio compared to lean subjects on the same diet [392]. Patients with metabolic disorders including diabetes mellitus and cardiovascular risk display serum microbiota dysbiosis on atherothrombotic disease [566]. In another study on 3280 participants without diabetes or obesity at baseline, the 16S rDNA concentration was higher in those destined to have diabetes and in those who had abdominal adiposity at the end of 9-year follow-up. The core blood microbiota consisted mostly of the Proteobacteria phylum (85–90%), suggesting that tissue bacteria are involved in the onset of diabetes in humans [567].

Patients with liver fibrosis ad obesity show specific differences in the proportion of several bacterial taxa, as detected by both faecal microbiota and blood microbiome profiles, which correlate with the presence of liver fibrosis [504].

In a Korean series [568], NAFLD patients showed different bacterial community and reduced diversity, as compared with controls. Further differences were noticed in terms of gut microbiota and serum microbiome profile, when lean and obese NAFLD subjects were compared. Different features (i.e., in lean NAFLD: decreased *Desulfovibrionaceae*, with an opposite trend in blood *Succinivibrionaceae*; in obese NAFLD: gut and blood *Leuconostocaceae*) might therefore become potential biomarkers to discriminate diverse NAFLD phenotypes. 

Members of Firmicutes were decreased in abundance in NAFLD [569]. 

In patients with histology-proven NAFLD (*n* = 57), the increase in *Bacteroides* genus count was linked with a twofold increase in NASH, and an elevated *Ruminococcus* count was associated with a twofold increase in stage 2 or greater fibrosis, [474,570]. As compared with healthy subjects, subjects with NAFLD have been reported as having a lower percentage of species Bacteroidetes and higher levels of *Porphyromonas* and *Prevotella* [499].

In the enterohepatic circulation, BA undergoes biotransformation to secondary BA by the resident colonic microbiota [4,5,571]. As mentioned earlier, a fat-enriched diet can re-shape the gut mucus layer [47,48] predisposing to dysbiosis. In this environment, the increased deconjugation of BAs can explain the hepatocellular injury induced by more cytotoxic secondary BA and inactivation of hepatic lipotropes including choline. Of note, a choline-deficient diet in the rat induces NAFLD [533,572,573,574,575]. A direct activation of NLRP3 inflammasome by BA promotes liver inflammation or fibrosis. C57BL/6 wild-type (WT) and Nlrp3−/− mice fed with a diet supplemented with cholic (CA), deoxycholic (DCA) or lithocholic acid (LCA) for 7 days showed activation of NLRP3 inflammasome. LCA mainly affected ex vivo Kuppfer cells, leading to a pro-inflammatory condition, with liver damage improved in Nlrp3-deficient mice or cells. Liver fibrosis was promoted by DCA feeding, with an upregulation in NLRP3 in primary hepatic stellate cells. These signals, however, were decreased in Nlrp3−/− mice or cells [576].

The NAFLD spectrum encompasses liver cirrhosis, which is characterized by more abundant genus level, Bacteroides, Streptococcus, Ruminococcus, Klebsiella, Prevotella, Enterococcus, Haemophilus, Lactobacillus, Pseudomonas, Phascolarctobacterium, Veillonella, Atopobium, Parabacteroides, Dialister, Christensenella, and decreased Methanobrevibacter and Akkermansia [577]. To what extent dysbiosis depends on early metabolic changes or late complications of disease, is still a matter of debate.

#### 9.3.2. Alcohol

Additional aspects depending on alcohol effect on the intestine require attention in the following context. The metabolism of alcohol in the body is mainly based on dietary ethanol which is able to cross the gastrointestinal mucosa in the stomach (~20%) and small intestine (~70%) by simple diffusion [578]. In the liver, ethanol is transformed into acetaldehyde (by the alcohol dehydrogenase), and to acetate (by the acetaldehyde dehydrogenase) [579,580]. Notably, the gut microbiota and enterocytes are equipped with alcohol-metabolizing enzymes which contribute to enriching the pool of gut alcohol with a small amount and as a product of luminal microbial fermentation [580,581]. Evidence in the animal models and human studies point to a role for both exogenous and endogenous ethanol at the gut and hepatic level. The damage can range from dysbiosis to gut dysmotility [275,276], mucosal damage and inflammation extending to chronic systemic low-grade inflammation [582]. Altogether, changes can contribute to an increase in gut permeability [583,584,585,586]. In addition, gut-derived alcohol may contribute to fatty liver disease.

In the Caco2 cellular model ethanol and acetaldehyde can damage the gut TJs (zonulin 1 and occludin) [587]. In biopsies of human colonic mucosa, acetaldehyde induced tyrosine phosphorylation and disrupted tight junction and adherens junction, and the effect was prevented by EGF and glutamine [588]. In another study using Caco2 cell cultures, the EGF-mediated protection of tight junctions from acetaldehyde required the activity of ERK1/2 but was independent from p38 MAPK or JNK1/2 [589].

Alcohol-fed mice developed bacterial overgrowth and enteric dysbiosis testified by the relative abundance of Bacteroidetes and *Verrucomicrobia* bacteria, compared with control mice which hosted a relative predominance of Firmicutes bacteria. In addition, alcohol feeding was associated with down-regulation in gene and protein expression of bactericidal c-type lectins Reg3b and Reg3g in the small intestine. Notably, treatment with prebiotics partially restored Reg3g protein levels, reduced bacterial overgrowth, and decreased alcoholic steatohepatitis [278]. The role of mucin within the intestinal barrier in relation to the effect of ethanol should not be neglected. In the Tsukamoto-French method for alcohol-induced liver disease obtained by continuous intragastric feeding of an isocaloric diet or alcohol, Muc2(-/-) mice showed less alcohol-induced liver injury, steatosis, plasma lipopolysaccharide than the wild-type mice. Muc2(-/-) mice were protected from alcohol-associated dysbiosis and had higher expression of jejunal antimicrobial proteins regenerating islet-derived 3 beta and gamma. This study shows that Muc2(-/-) mice are protected from gut bacterial overgrowth and dysbiosis in response to alcohol feeding. As anticipated, lower amounts of bacterial products such as endotoxin translocate into the systemic circulation, decreasing liver disease [590]. In another study using the Tsukamoto-French method for alcohol-induced liver disease, alcohol caused gut dysbiosis, reducing the capacity of the microbiome to synthesize saturated LCFA and the proportion of Lactobacillus species [591]. Germ-free mice have increased hepatic expression of ethanol-metabolizing genes and exacerbation in hepatic steatosis [592]. Xie et al. performed a detailed metabolomic study on rats that were fed for 8 weeks with ethanol. Ethanol consumption was associated with altered BA, increased fatty acids and steroids, decreased carnitines and metabolites involved in lipid metabolism, a significant decrease of all amino acids and branched chain amino acids, and significantly decreased SCFA (except for elevated acetic acid, as a product of ethanol metabolism) [593]. In a previous study on ob/ob mice, breath ethanol decreased with a course of non-absorbable antibiotics, suggesting that the ethanol is derived from gut bacterial flora [594]. In another study, breath was collected from genetically obese, ob/ob male C57BL/6 mice and lean male littermates at different ages (14, 20, and 24 weeks). Even in the absence of ethanol ingestion, ethanol was detected in exhaled breath and in obesity, an age-related increase in breath ethanol content likely reflected increased production of ethanol by the gut microflora and contribute to the genesis of obesity-related fatty liver [595]. Increased endogenous ethanol production occurs in animals with gut blind-loops [596].

Evidence points to the effect of alcohol metabolism also in the human model. Humans display high concentrations of endogenous alcohol production [597]. Patients who were obese were more likely to have higher breath ethanol concentrations, suggesting that gut-derived ethanol may contribute to the pathogenesis of NASH [598].

In humans, antigens derived from lipid peroxidation in alcoholics contribute to the development of the host inflammatory immune responses associated with alcoholic liver disease [599]. Studies on metabolomic analysis of faecal volatile organic compounds (VOC) demonstrate that alcoholics show distinct profiles compared to non-alcoholic individuals, which include increased oxidative stress biomarker tetradecane, decreased fatty alcohols with antioxidant property and decreased SCFA propionate and isobutyrate (which contribute to gut epithelial cell health and barrier integrity). In addition, in faecal samples from alcoholics decreased caryophyllene (natural suppressant in alcohol consumption), camphene (natural product and hepatic steatosis attenuator), and dimethyl disulfide and dimethyl trisulfide (two microbial products of decomposition) were reported [600]. 

Obese females with *Candida albicans* overgrowth have increased breath alcohol levels after a carbohydrate load [601]. Colonic bacteria and yeast are able to produce both ethanol and acetaldehyde [595]. The microbiota oxidizes low concentrations of ethanol to high concentrations of acetaldehyde which is absorbed into the portal blood stream. These pathways promote endotoxin-induced activation of Kupffer’s cells and therefore initiate histological changes similar to those occurring in NAFLD [602]. Proteobacteria such as Enterobacteriaceae can ferment carbohydrates to considerable concentration of ethanol [499,603], and in children the abundance of Proteobacteria, Enterobacteriaceae and *Escherichia* correlates with liver inflammation (NASH) and serum levels of alcohol [499]. Fasting ethanol levels were significantly higher in children with NAFLD than in controls and were positively associated with insulin resistance. Beside the increased synthesis of endogenous ethanol, the increased blood ethanol levels in NAFLD patients can also depend on insulin-dependent impairments of ADH activity in liver tissue [604]. Bacteria of the genus *Ruminococcus*, also part of the “core gut microbiome” found in 90% of humans, ferment complex carbohydrates such as cellulose with production of ethanol [605]. Non-alcoholic and alcoholic liver disease show increased luminal and circulating levels of ethanol, acetaldehyde and acetate [559,606], and metabolites are independently associated with liver damage [607,608,609]. Notably, increased ethanol and metabolite production will activate metabolic pathways and oxidative stress in the liver [610], contributing to the pathogenesis of NASH [499].

#### 9.3.3. Bile Acids (BA)

Bile and pancreatic fluid shape the gut microbiota [64,475,611]. Gut dysbiosis and survival develop in the absence of antimicrobial secretion by acinar cells. Indeed, deletion of Orai1 in pancreatic acinar cells of adult mice was associated with high mortality as a result of severe gut bacterial outgrowth with dysbiosis, in spite of an intact and fully activated gut innate immune response [611]. In the gut, the secondary BA interact with various nuclear receptors such as FXR, TGR5, pregnane X receptor, or vitamin D receptor [612]. Drugs participate in this scenario since metformin decreases *Bacteroides fragilis* while increasing the murine BA glycoursodeoxycholic acid (GUDCA). This effect, in turn, inhibits the gut FXR activation explaining some metabolic benefits [253].

In rodents, the postoperative bile diversion improvement in glycemia (but not changes in body weight or food intake) required FXR-Glp-1 axis activation and was associated with an increase in gut *A. muciniphila* [613]. BA changes after bariatric surgery by laparoscopic adjustable gastric banding promote metabolic benefits through increased conjugated and secondary BA (3 months after surgery with only glycolithocholic acid sulfate significantly elevated even after 1 year [614]. GLP-1 and fibroblast growth factor 19 (FGF-19) levels also correlated with BA levels. FXR stimulation by specific BA could become a therapeutic strategy for NAFLD [615].

The gut microbiome contributes to the final proportional profile of BA in terms of primary, secondary, and tertiary BAs. The colonic biotransformation of the primary BAs to the secondary BAs includes deconjugation, oxidation of hydroxyl groups in 3, 7 and 12 positions, and 7-dehydroxylation [616]. During the process of the enterohepatic circulation BA and gut bacteria share a bidirectional crosstalk [11,300,617]. The secondary DCA has antimicrobial properties due to the detergent effects on bacterial cell membranes. This feature can influence the bacteria integrity and contributes to shaping microbial populations [618]. Activation of the gut FXR by BA will promote the synthesis of peptides with antimicrobial effect (AMPs) such as angiogenin 1, RNAase family member 4 [264,619]. This effect has preventive action on intestinal barrier disruption. Modification of the characteristics of the BA pool can promote FXR-dependent effect on intestinal barrier and inflammation [265], metabolic pathways [620], and carcinogenic effects [621]. FXR-deficient mice do not develop diet-induced obesity [620], and mechanisms likely include changes of gut FXR [622] and microbiome [619]. Obstructed bile flow predisposes to gut bacterial overgrowth and translocations, while oral administration of BAs in the mouse model can reverse this condition. The FXR-induced gene expression might modulate this pathway associated with the enteral protection and the inhibition of bacteria damage to the gut mucosa [264].

With NAFLD, several changes point to a role for gut microbiota, BA profile, intestine and liver damage. An early study found that biopsy-proven NASH patients had higher fasting and post-prandial exposure to BA, which include the more hydrophobic and cytotoxic secondary species. Increased bile acid exposure can promote liver injury of NAFLD and NASH [623].

In a comprehensive study in NAFLD individuals and high-fat fed rats, Jao et al. [624] demonstrated that NAFLD was associated with increased serum concentrations of primary and secondary BA. The FXR antagonistic DCA was increased while the agonistic CDCA was decreased in parallel with reduced serum FGF19 pointing to impaired FXR and fibroblast growth factor receptor 4 (FGFR4)-mediated signaling. In addition, taurine and glycine metabolising bacteria were increased in the gut of NAFLD patients, reflecting increased secondary BA production. Mouzaki et al. found that, compared to the control, NASH patients had increased levels of total faecal BA, CA, CDCA, and BA synthesis and a higher primary to secondary BA ratio. Bacteroidetes and *Clostridium leptum* counts were decreased in a subset of NASH patients suggesting that, in NAFLD, dysbiosis is associated with altered BA homeostasis and an increased risk of hepatic injury [625]. Additional evidence shows that an abundance of DCA-producing bacteria can explain the DCA-dependent suppression of FXR- and FGFR4-mediated signaling [3,624]. In this respect, the expression of gut FXR decreases in mice on a high-fiber diet, and obeticholic acid restores the integrity of the gut vascular barrier and reduces the portal influx of PAMP to the liver [113].

#### 9.3.4. SCFA including Propionate

SCFAs are produced during the degradation (fermentation) of nondigestible nutritional fibers by bacteria and include propionate (C3), butyrate (C4), and acetate (C2). SCFAs provide an energy source to enterocytes, contribute to faecal acidification, and have a variety of anti-inflammatory properties in immune cells (T cells, regulatory T cells (Tregs), neutrophils, and macrophages) where they control migration, cytolytic activity, cytokine production, microbicidal activity [105,108], and epigenetic regulation of gene expression [26]. 

SCFAs are greatly produced by gut microbiota [29], providing an energetic substrate for colon cells, contributing to an adequate efficiency of the intestinal barrier and modulating inflammation processes and satiety [626,627,628]. A rise in acetate levels might indicate increased production and liver metabolism of endogenous ethanol by intestinal microbiota. On the other hand, reduced butyrate levels might indicate altered tight junctions and increased intestinal permeability [583,591]. In this respect, the administration of tributyrin (the glycerol ester providing butyrate) is able to improve gut permeability and to reduce liver injury in mice fed on an alcohol-enriched diet [583].

In mice, faecal SCFA correlate inversely with the presence of *Bacteroides* and positively with *Alistipes*, *Barnesiella*, and *Prevotella* [629].

Propionate is a ligand of Gpr41 [630] and GPR41 knockout mice fed on a high-fat diet; male mice increased body fat content. These results suggest that gut-derived SCFA may raise energy expenditure and help to protect against obesity by activating GPR41 [631]. Among the beneficial effects on metabolism, SCFA can promote gluconeogenesis and lipogenesis [632,633]. Beneficial effects in liver steatosis have been documented in the animal model [634,635]. Butyrate counteracts the HFD-induced obesity and insulin resistance [438]. Inhibition of histone deacetylases (HDACs) by SCFA also promotes regulatory T cells and reduces insulin resistance [636,637,638,639].

#### 9.3.5. Fasting-Induced Adipocyte Factor (Fiaf)

The gut L cells and enterocytes produce the fasting-induced adipocyte factor (Fiaf), a member of the angiopoietin-like family of proteins. Fiaf is a circulating lipoprotein lipase inhibitor, but microbial suppression of Fiaf in the gut epithelium will increase the microbiota-induced deposition of triglycerides in adipocytes, via increased lipoprotein lipase (LPL) activity in adipocytes. The microbiota in the intestine is involved in the processing of dietary polysaccharides and increased hepatic lipogenesis (by activation of the carbohydrate responsive element-binding protein (ChREBP) and the sterol regulatory element-binding protein-1 (SREB-1)). These microbiota-dependent steps coordinate increased hepatic lipogenesis and triglyceride storage in adipocytes, a benefit that becomes detrimental in Westernized societies and obesogenic (hypercaloric) environments [427].

#### 9.3.6. Trimethylamine (TMA)

Phosphatidylcholine, choline, and carnitine are abundant in meat, eggs, and high-fat diets. The essential macronutrient choline is metabolized in lecithin and contributes to the hepatic assembly of very-low density lipoprotein (VLDL), and to their excretion by the liver, thus preventing triglyceride accumulation and the subsequent onset of liver steatosis [640]. In fact, mice fed a choline-deficient diet develop oxidative stress and liver steatosis [533,572,574,575,641]. Choline is also used by gut microbiota to synthetize trimethylamine (TMA), which is subsequently converted to TMA-N-oxide (TMAO) by the liver enzyme flavin-containing monooxygenase 3 (FMO3). Gut bacteria of taxa *Erysipelotrichia* produce TMA following choline metabolization, thus decreasing the bioavailability of this macronutrient and increasing the portal influx of TMA and its conversion to trimethylamine N-oxide (TMAO). This small metabolite [642,643,644,645,646,647,648] is associated with atherosclerosis and cardiovascular complications, including myocardial infarction and stroke [646,649,650,651,652]. In addition, increased TMAO levels correlate with the presence of type 2 diabetes mellitus, glycemic control in type 2 diabetes mellitus, its complications, and steatogenic effects [653,654,655,656,657,658,659].

Suppressing the gut microbiota in atherosclerosis-prone mice inhibited dietary-choline-enhanced atherosclerosis [651], whereas NAFLD patients show increased gut metabolism of choline, choline deficiency, and abundance of *Erysipelotrichia* taxa [658].

#### 9.3.7. Phenylacetate

A study focusing on gut microbiome and hepatic transcriptome [660] found decreased microbial gene richness associated with increased branched chain amino acids (BCAA). *Actinobacteria*, *Proteobacteria*, and *Verrucomicrobia* occurrence was increased, and phenylacetate, one bacteria-derived metabolite, was highly associated with steatosis. At a pathophysiological level, the mechanisms involved are of interest, since phenylacetate is a metabolite of essential amino acids such as phenylalanine and tyrosine, and likely enhances hepatic lipid accumulation via increased BCAA utilization, while promoting hepatic steatosis in mice.

#### 9.3.8. Imidazole Propionate

Imidazole propionate is a gut microbial product from histidine, regulating insulin signaling without the involvement of immune or inflammatory changes [661]. Type 2 diabetes mellitus patients display higher circulating levels of Imidazole propionate which interacts with insulin signaling by the activation of p38g MAPK, phosphorylation of p62, and ultimately activation of mechanistic target of rapamycin (mTORC1).

#### 9.3.9. Other Metabolites

The presence of gut Firmicutes, Bacteroidetes, and Proteobacteria correlate with metabolites such as 3-(4-hydroxyphenyl) lactate and hepatic fibrosis [570]. Other microbiome-derived metabolites from branched-chain and aromatic amino acid can also play a role in NAFLD. Phenylacetic acid and 3-(4-hydroxyphenyl)-lactate can be related to insulin resistance. Obese, non-diabetic steatotic patients show low microbial gene richness and increased microbial genetic potential for processing dietary lipids and endotoxin biosynthesis from Proteobacteria. Both aromatic and branched-chain amino acid metabolism are dysregulated [660].

## 10. NAFLD and Gut Permeability

Animal studies point to a role for abnormal gut permeability in NAFLD. Steatotic mice develop endotoxin, triggering liver inflammation [662]. Obese mice, such as C57BL/6Job/ob genetically leptin deficient and C57BL/6Jdb/db functionally deficient for the long-form leptin receptor, have increased epithelial permeability to horseradish peroxidase. Compared to control mice, both groups of obese mice showed abnormal distribution of ZO-1 and Occludin TJ proteins, increased circulating levels of endotoxin in portal circulation and levels of circulating proinflammatory cytokines (IL-1, IL-6, INF-γ, and TNF-α). In the liver, HSC were activated with enhanced sensitivity to LPS and increased levels of cytokines [120]. The murine gene *F11r* encodes the junctional adhesion molecule A (JAM-A), a constituent of the TJs which regulates permeability and inflammation [663,664,665,666]. Notably, F11r−/− mice fed a high-saturated fat, -fructose, and -cholesterol steatogenic diet for 8 weeks developed severe steatohepatitis (hepatocyte ballooning and inflammatory cells infiltration), fibrogenesis, and increased in serum transaminases, compared with control animals [416]. Gut inflammation exacerbates liver injury and fibrosis in HFD mice and contributes to the development of NASH. Abnormalities include damaged gut epithelial barrier, gut vascular barrier disruption with bacterial translocation towards lymph nodes and even the liver. In C57BL/6 mice fed a high-fat diet (HFD) for 12 weeks, gut inflammation and gut vascular barrier dysfunction induced by dextran sulfate sodium (DSS) was associated with liver fat vacuoles and leukocyte infiltration, increased levels of hepatic mRNA coding for inflammatory cytokines (IL-1, IL-6, TNF-α, MCP-1), higher expression of collagen I and profibrogenic factors mRNA (TGF-β, Actin α2, tissue inhibitor of metalloproteinase-1 and plasminogen activator inhibitor-1). The study documented upregulation of TLR4 and TLR 9, downregulation of ZO-1 and Claudin-1, and increased expression of PV1. Changes were more evident than those observed in HFD-fed mice. [245]. 

Human studies point to a role for increased gut permeability and liver damage, as shown in NAFLD patients [559,667]. Luther et al. [496] performed a metanalysis to compare the rates of increased gut permeability in patients with NASH and healthy controls and studied changes in gut permeability in a diet-induced (methionine-and-choline-deficient) murine model of NASH. The effect of methionine-and-choline-deficient culture medium was studied on hepatocytes, Kupffer cells, and gut epithelial cells. This study confirms that NAFLD/NASH patients are more likely to have increased gut permeability compared with healthy controls. In addition, mice and cellular experiments point to an early phase of hepatic injury and inflammation contributing to altered gut permeability in a TNFa- and myosin light-chain kinase (MLCK)-independent fashion. NAFLD patients can show small intestinal bacterial overgrowth [559,668,669,670,671] and dysbiosis [490], and the ongoing abnormal gut permeability acts as a factor leading to liver inflammation and fibrosis [672]. Additional factors include disrupted TJs and small intestinal bacterial overgrowth in NAFLD patients. A previous trial compared 22 biopsy-proven NASH patients with 23 controls subjects and assessed gut overgrowth by (14)C-D-xylose and lactulose breath test, gut permeability by a dual lactulose-rhamnose sugar test, serum endotoxin levels by limulus amoebocyte lysate assay, and TNF-alpha levels by ELISA. Small intestinal bacterial overgrowth occurred in 50% of patients and 22% of controls (*p* = 0.048), gut permeability and serum endotoxin levels were similar between the two groups, but the endotoxin assay confirmed significantly higher TNF-α levels in patients than controls (14.2 and 7.5 pg/mL, respectively, *p* = 0.001) [495]. Another study investigated small intestinal bacterial overgrowth (by glucose hydrogen breath test), gut permeability (by urinary excretion of (51)Cr-ethylene diamine tetraacetate ((51)Cr-EDTA), and immunohistochemical analysis of zona occludens-1 (ZO-1) expression in duodenal biopsy specimens as a marker of the integrity of TJs in patients with biopsy-proven NAFLD (*n* = 35), patients with untreated celiac disease (*n* = 27) and healthy subjects (*n* = 24). Notably, orally administered ^51^Cr-EDTA is not metabolized and is poorly absorbed (1%-3%) from the gastrointestinal tract, but with TJs disruption ^51^Cr-EDTA crosses the intestinal barrier through the paracellular pathway [59,673,674]. NAFLD patients had significantly increased gut permeability and three times the small intestinal bacterial overgrowth compared to control [559]. ^51^Cr-EDTA excretion levels and small intestinal bacterial overgrowth prevalence increased with the degree of liver steatosis. At histology, NAFLD patients had reduced duodenal ZO-1 expression. Increased gut permeability and small intestinal bacterial overgrowth were independent with the severity of liver inflammation, fibrosis, and NASH. NALFD children had increased gut permeability as confirmed by urinary excretion of orally administered lactulose and mannitol (L/M ratio) [495,560,675]. L/M ratio further increased in NASH patients. The increased LPS is a marker of bacterial translocation while the extent of hepatic inflammation and fibrosis is proportional to the degree of gut permeability [560]. As compared with healthy controls, children with mild NAFLD (simple steatosis grade 1) show higher serum levels of alanine aminotransferase, inflammatory markers and insulin resistance [676]. NAFLD children also showed higher levels of plasma bacterial endotoxin (+50%) and lipopolysaccharide-binding protein (LBP, +24%). Results from the cited study also revealed a positive association between plasma endotoxin/LBP levels and the proinflammatory markers plasminogen activator inhibitor-1, c-reactive protein, interleukin-6 and leptin, with no effects from the extent of insulin resistance. These data point to the possibility of an alter intestinal barrier already present in the early phase of pediatric NAFLD.

The homeostatic mechanisms responsible for normal intestinal permeability can be altered in the presence of diffused liver diseases as cirrhosis and NAFLD [136,677,678].

**Figure 2:**
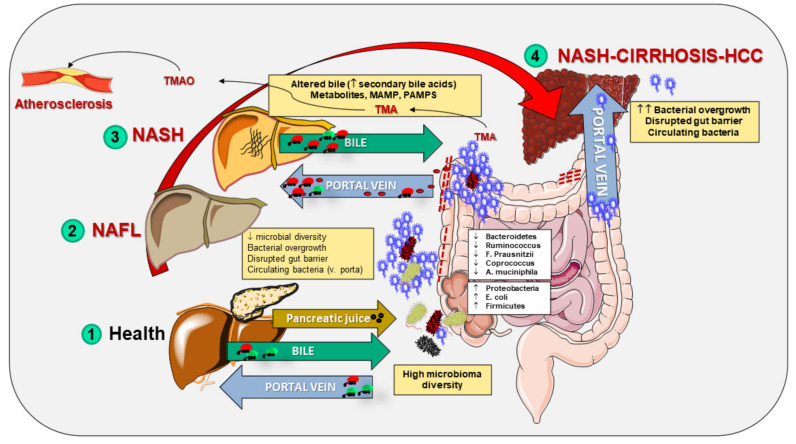
Potential progression of changes in the gut and the liver with ongoing nonalcoholic fatty liver disease. (1) In health, the gut microbiota has high diversity of microbial species to guarantee all physiological tasks. Both bile secretion and pancreatic juice contribute to shaping the gut microbiota. The ratio of primary (green color) to secondary bile acids (red color) is under the control of the healthy gut microbial population (see text for details). (2) With the accumulation of triglycerides, long-chain fatty acids and their metabolites in the liver (simple steatosis, nonalcoholic fatty liver, NAFL), gut microbiota can be reshaped by decreased microbial diversity, small gut overgrowth, disrupted intestinal barrier and circulating bacteria in the portal tract. (3) A further step includes the progressive necro-inflammatory and fibrotic form nonalcoholic steatohepatitis (NASH). This evolution is often associated with the rise in pro-inflammatory and pro-steatotic bacterial products in the portal circuit. Changes of the bile acid pool (a shift to increased cytotoxic secondary bile acids, deoxycholic acid, lithocholic acid by bacterial deconjugation especially in the colon) will increase the delivery of these bile acids via the portal vein to the liver, driving a further damage. The intestinal barrier will further increase the permeability, and mechanisms of damage will be perpetuated. (4) If the sequence NASH-Cirrhosis (and even hepatocellular carcinoma, HCC) develops, the intestinal barrier will be further disrupted and, culturable bacteria can translocate via the portal vein to the systemic circulation. The role of bacterial-gut-derived metabolites with systemic effects is shown with trimethylamine (TMA) produced by bacteria out of dietary compounds, is metabolized in the liver to trimethylamine N-oxide (TMAO) which has pro-atherogenic effects and increases the risk of cardiovascular events.

In a recent study, we assessed urine recovery of orally administered sucrose, lactulose/mannitol, and sucralose by triple quadrupole mass-spectrometry and high-performance liquid chromatography. Increased colonic (but not stomach and small gut) permeability occurred in obesity and liver steatosis, regardless dietary habits, age, and physical activity [136]. A summary of key events involved in the progression of changes in the gut and the liver with ongoing nonalcoholic fatty liver disease are depicted in Figure 2.

## 11. Conclusions and Future Perspectives

**Figure 3:**
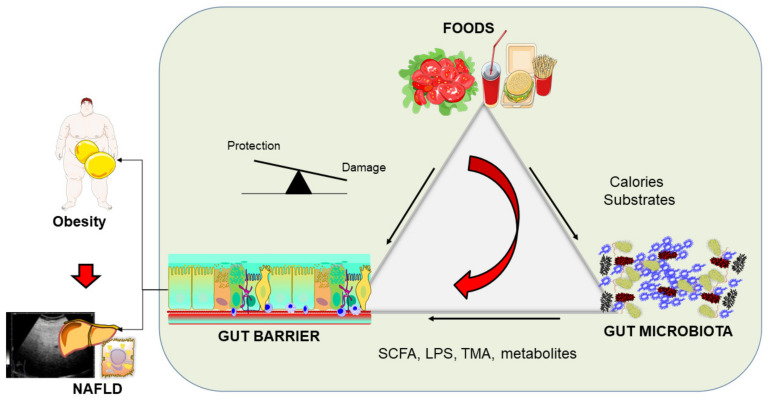
Relationships between foods, gut microbiota and intestinal barrier, as main contributors to obesity and nonalcoholic fatty liver disease (NAFLD). SCFA, short-chain fatty acids, LPS, lipopolysaccharides; TMA, trimetylamine.

The gut surface is exposed to the external environment and consists of a complex anatomical and functional structure encompassing the intestinal barrier. In health and disease, nutrients and metabolites interact with the gut microbiota, mucin, motility and secretions, enterocytes and junctions, immune responses, and the vascular and hepatic barrier to control and prevent the abnormal translocation and permeability of bacteria, bacterial products, and metabolites. The gut microbiota can be re-shaped by exogenous and endogenous actors and events and display a rather specific signature. The increased expression of Gram-negative bacteria such as Enterobacteria, *E. coli*, and Proteobacteria can elaborate a pro-inflammatory phenotype which includes the endotoxin [136,559,679]. The gut–liver interaction drives changes bidirectionally, and factors include BA, immunity, and gut permeability as well. Mechanisms of gut permeability can be disrupted in frequent conditions including obesity and the metabolically associated liver steatosis (nonalcoholic fatty liver disease, NAFLD) (Figure 3). Consequences of impaired gut permeability represent predisposing or aggravating factors in both obesity and NAFLD. There is evidence that the close crosstalk between the gut and microbiome is essential in keeping health and can be involved in diseases, including metabolic disorders.

## Figures and Tables

**Table 1:** Main food groups and fiber varieties according to solubility.

Food Group	Soluble Fibers	Insoluble Fibers
Cereals and grains	Nonstarch polysaccharides	Nonstarch polysaccharides
	Hemicellulose Arabinoxylan β-glucan	Hemicellulose Cellulose Lignin
	Resistant oligosaccharides	Resistant starch
	Inulin	
Fruits and vegetables	Nonstarch polysaccharides	Nonstarch polysaccharides
	Hemicellulose	Hemicellulose
	Pectin	Cellulose
		Pectin
	Resistant oligosaccharides	Lignin
	Inulin	Resistant starch
Legumes and pulses	Nonstarch polysaccharides	Nonstarch polysaccharides
	Hemicellulose	Hemicellulose
	Pectin	Pectin
	Gum	
		Lignin
		Resistant starch

Adapted from Swann et al., 2019 [142] and Institute of Medicine, 2005 [139].

**Table 2:** Most frequent causes of liver steatosis and relative prevalence in each pathologic condition.

Metabolic, nonalcoholic fatty liver disease (NAFLD, 23–69%) [307]
Alcoholic fatty liver disease (ALD) (about 5%) [308]
Viral hepatitis B and C (especially genotype 3) (30–80%) [309,310,311,312]
Lipodystrophy (80%) [313]
Wilson’s disease (about 50%) [314]
Starvation (prevalence undetermined)
Parenteral nutrition (about 28%) [315]
Abetalipoproteinemia (prevalence undetermined)
Hepatotoxic drugs (about 2%) [316] (amiodarone, anti-retroviral agents for HIV, glucocorticoids, methotrexate, tamoxifen, valproate)
Pregnancy (incidence 1:7000–15,000 pregnancies) [317]
Reye syndrome (100%) [318]
Inborn errors of metabolism (about 40% of children hospitalized for nonalcoholic fatty liver disease) [319] (lecithin-cholesterol acyltransferase deficiency, cholesterol ester storage disease, Wolman disease)

## References

[1] Dommett R., Zilbauer M., George J.T., Bajaj-Elliott M. (2005). Innate immune defence in the human gastrointestinal tract. Mol. Immunol..

[2] Brandl K., Kumar V., Eckmann L. (2017). Gut-liver axis at the frontier of host-microbial interactions. Am. J. Physiol. Gastrointest. Liver Physiol..

[3] Tripathi A., Debelius J., Brenner D.A., Karin M., Loomba R., Schnabl B., Knight R. (2018). The gut-liver axis and the intersection with the microbiome. Nat. Rev. Gastroenterol. Hepatol..

[4] Di Ciaula A., Garruti G., Lunardi Baccetto R., Molina-Molina E., Bonfrate L., Wang D.Q., Portincasa P. (2017). Bile Acid Physiology. Ann. Hepatol..

[5] Garruti G., Di Ciaula A., Wang H.H., Wang D.Q., Portincasa P. (2017). Cross-Talk Between Bile Acids and Gastro-Intestinal and Thermogenic Hormones: Clues from Bariatric Surgery. Ann. Hepatol..

[6] Garruti G., Wang D.Q., Di Ciaula A., Portincasa P. (2018). Cholecystectomy: A way forward and back to metabolic syndrome?. Lab. Invest..

[7] Portincasa P., Di Ciaula A., Garruti G., Vacca M., De Angelis M., Wang D.Q. (2020). Bile Acids and GPBAR-1: Dynamic Interaction Involving Genes, Environment and Gut Microbiome. Nutrients.

[8] Nicoletti A., Ponziani F.R., Biolato M., Valenza V., Marrone G., Sganga G., Gasbarrini A., Miele L., Grieco A. (2019). Intestinal permeability in the pathogenesis of liver damage: From non-alcoholic fatty liver disease to liver transplantation. World J. Gastroenterol..

[9] Okumura R., Takeda K. (2018). Maintenance of intestinal homeostasis by mucosal barriers. Inflamm. Regen..

[10] Meyer-Hoffert U., Hornef M.W., Henriques-Normark B., Axelsson L.G., Midtvedt T., Putsep K., Andersson M. (2008). Secreted enteric antimicrobial activity localises to the mucus surface layer. Gut.

[11] Di Ciaula A., Baj J., Garruti G., Celano G., De Angelis M., Wang H.H., Di Palo D.M., Bonfrate L., Wang D.Q.-H., Portincasa P. (2020). Liver Steatosis, Gut-Liver Axis, Microbiome and Environmental Factors. A Never-Ending Bidirectional Cross-Talk. J. Clin. Med..

[12] Savage D.C. (1977). Microbial ecology of the gastrointestinal tract. Annu. Rev. Microbiol..

[13] Kallus S.J., Brandt L.J. (2012). The intestinal microbiota and obesity. J. Clin. Gastroenterol..

[14] Huttenhower C., Gevers D., Knight R., Abubucker S., Badger J.H., Chinwalla A.T., Creasy H.H., Earl A.M., FitzGerald M.G., Fulton R.S. (2012). Structure, function and diversity of the healthy human microbiome. Nature.

[15] McGhee J.R., Fujihashi K. (2012). Inside the mucosal immune system. PLoS Biol..

[16] Sender R., Fuchs S., Milo R. (2016). Are We Really Vastly Outnumbered? Revisiting the Ratio of Bacterial to Host Cells in Humans. Cell.

[17] Lynch S.V., Pedersen O. (2016). The Human Intestinal Microbiome in Health and Disease. New Engl. J. Med..

[18] Jandhyala S.M., Talukdar R., Subramanyam C., Vuyyuru H., Sasikala M., Nageshwar Reddy D. (2015). Role of the normal gut microbiota. World J. Gastroenterol..

[19] Gilbert J.A., Blaser M.J., Caporaso J.G., Jansson J.K., Lynch S.V., Knight R. (2018). Current understanding of the human microbiome. Nat. Med..

[20] Maslowski K.M., Mackay C.R. (2011). Diet, gut microbiota and immune responses. Nat. Immunol..

[21] Hooper L.V., Macpherson A.J. (2010). Immune adaptations that maintain homeostasis with the intestinal microbiota. Nat. Rev. Immunol..

[22] De Lacy Costello B., Amann A., Al-Kateb H., Flynn C., Filipiak W., Khalid T., Osborne D., Ratcliffe N.M. (2014). A review of the volatiles from the healthy human body. J. Breath Res..

[23] Guarner F., Malagelada J.R. (2003). Gut flora in health and disease. Lancet.

[24] Reynes B., Palou M., Rodriguez A.M., Palou A. (2018). Regulation of Adaptive Thermogenesis and Browning by Prebiotics and Postbiotics. Front. Physiol..

[25] Said H.M., Ortiz A., McCloud E., Dyer D., Moyer M.P., Rubin S. (1998). Biotin uptake by human colonic epithelial NCM460 cells: A carrier-mediated process shared with pantothenic acid. Am. J. Physiol..

[26] Biesalski H.K. (2016). Nutrition meets the microbiome: Micronutrients and the microbiota. Ann. N. Y. Acad. Sci..

[27] Belancic A. (2020). Gut microbiome dysbiosis and endotoxemia—Additional pathophysiological explanation for increased COVID-19 severity in obesity. Obes. Med..

[28] Salguero M.V., Al-Obaide M.A.I., Singh R., Siepmann T., Vasylyeva T.L. (2019). Dysbiosis of Gram-negative gut microbiota and the associated serum lipopolysaccharide exacerbates inflammation in type 2 diabetic patients with chronic kidney disease. Exp. Ther. Med..

[29] Sonnenburg J.L., Backhed F. (2016). Diet-microbiota interactions as moderators of human metabolism. Nature.

[30] Pelaseyed T., Bergstrom J.H., Gustafsson J.K., Ermund A., Birchenough G.M., Schutte A., van der Post S., Svensson F., Rodriguez-Pineiro A.M., Nystrom E.E. (2014). The mucus and mucins of the goblet cells and enterocytes provide the first defense line of the gastrointestinal tract and interact with the immune system. Immunol. Rev..

[31] Ismail A.S., Severson K.M., Vaishnava S., Behrendt C.L., Yu X., Benjamin J.L., Ruhn K.A., Hou B., DeFranco A.L., Yarovinsky F. (2011). γδ intraepithelial lymphocytes are essential mediators of host–microbial homeostasis at the intestinal mucosal surface. Proc. Natl. Acad. Sci. USA.

[32] Johansson M.E., Phillipson M., Petersson J., Velcich A., Holm L., Hansson G.C. (2008). The inner of the two Muc2 mucin-dependent mucus layers in colon is devoid of bacteria. Proc. Natl. Acad. Sci. USA.

[33] Kim Y.S., Ho S.B. (2010). Intestinal goblet cells and mucins in health and disease: Recent insights and progress. Curr. Gastroenterol. Rep..

[34] Vereecke L., Beyaert R., van Loo G. (2011). Enterocyte death and intestinal barrier maintenance in homeostasis and disease. Trends Mol. Med..

[35] Gibbins H.L., Proctor G.B., Yakubov G.E., Wilson S., Carpenter G.H. (2015). SIgA binding to mucosal surfaces is mediated by mucin-mucin interactions. PLoS ONE.

[36] Bergström J.H., Birchenough G.M.H., Katona G., Schroeder B.O., Schütte A., Ermund A., Johansson M.E.V., Hansson G.C. (2016). Gram-positive bacteria are held at a distance in the colon mucus by the lectin-like protein ZG16. Proc. Natl. Acad. Sci. USA.

[37] Camilleri M., Vella A. (2021). What to do about the leaky gut. Gut.

[38] Paone P., Cani P.D. (2020). Mucus barrier, mucins and gut microbiota: The expected slimy partners?. Gut.

[39] Tsilingiri K., Barbosa T., Penna G., Caprioli F., Sonzogni A., Viale G., Rescigno M. (2012). Probiotic and postbiotic activity in health and disease: Comparison on a novel polarised ex-vivo organ culture model. Gut.

[40] Tsilingiri K., Rescigno M. (2013). Postbiotics: What else. Beneficial Microbes 4.

[41] Levy M., Blacher E., Elinav E. (2017). Microbiome, metabolites and host immunity. Curr. Opin. Microbiol..

[42] Blacher E., Levy M., Tatirovsky E., Elinav E. (2017). Microbiome-modulated metabolites at the interface of host immunity. J. Immunol..

[43] Mosca F., Gianni M.L., Rescigno M. (2019). Can Postbiotics Represent a New Strategy for NEC. Probiotics and Child Gastrointestinal Health.

[44] Jakobsson H.E., Rodriguez-Pineiro A.M., Schutte A., Ermund A., Boysen P., Bemark M., Sommer F., Backhed F., Hansson G.C., Johansson M.E. (2015). The composition of the gut microbiota shapes the colon mucus barrier. EMBO Rep..

[45] Wrzosek L., Miquel S., Noordine M.L., Bouet S., Joncquel Chevalier-Curt M., Robert V., Philippe C., Bridonneau C., Cherbuy C., Robbe-Masselot C. (2013). Bacteroides thetaiotaomicron and Faecalibacterium prausnitzii influence the production of mucus glycans and the development of goblet cells in the colonic epithelium of a gnotobiotic model rodent. BMC Biol..

[46] Desai M.S., Seekatz A.M., Koropatkin N.M., Kamada N., Hickey C.A., Wolter M., Pudlo N.A., Kitamoto S., Terrapon N., Muller A. (2016). A Dietary Fiber-Deprived Gut Microbiota Degrades the Colonic Mucus Barrier and Enhances Pathogen Susceptibility. Cell.

[47] Mastrodonato M., Mentino D., Portincasa P., Calamita G., Liquori G.E., Ferri D. (2014). High-fat diet alters the oligosaccharide chains of colon mucins in mice. Histochem. Cell Biol..

[48] Liquori G.E., Mastrodonato M., Mentino D., Scillitani G., Desantis S., Portincasa P., Ferri D. (2012). In situ characterization of O-linked glycans of Muc2 in mouse colon. Acta Histochem..

[49] Birchenough G.M., Nystrom E.E., Johansson M.E., Hansson G.C. (2016). A sentinel goblet cell guards the colonic crypt by triggering Nlrp6-dependent Muc2 secretion. Science.

[50] Abreu M.T. (2010). Toll-like receptor signalling in the intestinal epithelium: How bacterial recognition shapes intestinal function. Nat. Rev. Immunol..

[51] Ouwerkerk J.P., de Vos W.M., Belzer C. (2013). Glycobiome: Bacteria and mucus at the epithelial interface. Best Pract. Res. Clin. Gastroenterol..

[52] Derrien M., Van Baarlen P., Hooiveld G., Norin E., Muller M., de Vos W.M. (2011). Modulation of Mucosal Immune Response, Tolerance, and Proliferation in Mice Colonized by the Mucin-Degrader Akkermansia muciniphila. Front. Microbiol..

[53] Dao M.C., Everard A., Aron-Wisnewsky J., Sokolovska N., Prifti E., Verger E.O., Kayser B.D., Levenez F., Chilloux J., Hoyles L. (2016). Akkermansia muciniphila and improved metabolic health during a dietary intervention in obesity: Relationship with gut microbiome richness and ecology. Gut.

[54] Grander C., Adolph T.E., Wieser V., Lowe P., Wrzosek L., Gyongyosi B., Ward D.V., Grabherr F., Gerner R.R., Pfister A. (2018). Recovery of ethanol-induced Akkermansia muciniphila depletion ameliorates alcoholic liver disease. Gut.

[55] Everard A., Belzer C., Geurts L., Ouwerkerk J.P., Druart C., Bindels L.B., Guiot Y., Derrien M., Muccioli G.G., Delzenne N.M. (2013). Cross-talk between Akkermansia muciniphila and intestinal epithelium controls diet-induced obesity. Proc. Natl. Acad. Sci. USA.

[56] Johansson M.E.V. (2012). Fast renewal of the distal colonic mucus layers by the surface goblet cells as measured by in vivo labeling of mucin glycoproteins. PLoS ONE.

[57] Johansson M.E., Sjovall H., Hansson G.C. (2013). The gastrointestinal mucus system in health and disease. Nat. Rev. Gastroenterol. Hepatol..

[58] Begley M., Gahan C.G.M., Hill C. (2005). The interaction between bacteria and bile. FEMS Microbiol. Rev..

[59] Ponziani F.R., Gerardi V., Gasbarrini A. (2016). Diagnosis and treatment of small intestinal bacterial overgrowth. Expert Rev. Gastroenterol. Hepatol..

[60] Kakiyama G., Pandak W.M., Gillevet P.M., Hylemon P.B., Heuman D.M., Daita K., Takei H., Muto A., Nittono H., Ridlon J.M. (2013). Modulation of the fecal bile acid profile by gut microbiota in cirrhosis. J. Hepatol..

[61] Inagaki T., Choi M., Moschetta A., Peng L., Cummins C.L., McDonald J.G., Luo G., Jones S.A., Goodwin B., Richardson J.A. (2005). Fibroblast growth factor 15 functions as an enterohepatic signal to regulate bile acid homeostasis. Cell Metab..

[62] Garruti G., Wang H.H., Bonfrate L., de Bari O., Wang D.Q., Portincasa P. (2012). A pleiotropic role for the orphan nuclear receptor small heterodimer partner in lipid homeostasis and metabolic pathways. J. Lipids.

[63] Liu H., Hu C., Zhang X., Jia W. (2018). Role of gut microbiota, bile acids and their cross-talk in the effects of bariatric surgery on obesity and type 2 diabetes. J. Diabetes Investig..

[64] Wahlström A., Sayin S.I., Marschall H.-U., Bäckhed F. (2016). Intestinal crosstalk between bile acids and microbiota and its impact on host metabolism. Cell Metab..

[65] Ory D.S. (2004). Nuclear receptor signaling in the control of cholesterol homeostasis: Have the orphans found a home?. Circ.Res..

[66] Kurashima Y., Kiyono H. (2017). Mucosal Ecological Network of Epithelium and Immune Cells for Gut Homeostasis and Tissue Healing. Annu. Rev. Immunol..

[67] Nevo S., Kadouri N., Abramson J. (2019). Tuft cells: From the mucosa to the thymus. Immunol. Lett..

[68] Turner J.R. (2009). Intestinal mucosal barrier function in health and disease. Nat. Rev. Immunol..

[69] Salzman N.H. (2010). Paneth cell defensins and the regulation of the microbiome: Détente at mucosal surfaces. Gut Microbes.

[70] Salzman N.H., Hung K., Haribhai D., Chu H., Karlsson-Sjöberg J., Amir E., Teggatz P., Barman M., Hayward M., Eastwood D. (2010). Enteric defensins are essential regulators of intestinal microbial ecology. Nat. Immunol..

[71] Bennett K.M., Walker S.L., Lo D.D. (2014). Epithelial microvilli establish an electrostatic barrier to microbial adhesion. Infect. Immun..

[72] Odenwald M.A., Turner J.R. (2017). The intestinal epithelial barrier: A therapeutic target?. Nat. Rev. Gastroenterol. Hepatol..

[73] Buckley A., Turner J.R. (2018). Cell Biology of Tight Junction Barrier Regulation and Mucosal Disease. Cold Spring Harb. Perspect. Biol..

[74] Yamazaki Y., Okawa K., Yano T., Tsukita S., Tsukita S. (2008). Optimized proteomic analysis on gels of cell-cell adhering junctional membrane proteins. Biochemistry.

[75] Schneeberger E.E., Lynch R.D. (2004). The tight junction: A multifunctional complex. Am. J. Physiol. Cell Physiol..

[76] Hollander D., Kaunitz J.D. (2020). The "Leaky Gut": Tight Junctions but Loose Associations?. Dig. Dis. Sci..

[77] Schoultz I., Keita Å.V. (2020). The Intestinal Barrier and Current Techniques for the Assessment of Gut Permeability. Cells.

[78] Van Itallie C.M., Anderson J.M. (2014). Architecture of tight junctions and principles of molecular composition. Semin. Cell Dev. Biol..

[79] Anderson J.M., Van Itallie C.M. (2009). Physiology and function of the tight junction. Cold Spring Harb. Perspect. Biol..

[80] Van Itallie C.M., Holmes J., Bridges A., Gookin J.L., Coccaro M.R., Proctor W., Colegio O.R., Anderson J.M. (2008). The density of small tight junction pores varies among cell types and is increased by expression of claudin-2. J. Cell Sci..

[81] Taylor C.T., Dzus A.L., Colgan S.P. (1998). Autocrine regulation of epithelial permeability by hypoxia: Role for polarized release of tumor necrosis factor alpha. Gastroenterology.

[82] Madara J.L., Stafford J. (1989). Interferon-gamma directly affects barrier function of cultured intestinal epithelial monolayers. J. Clin. Investig..

[83] Turner J.R., Rill B.K., Carlson S.L., Carnes D., Kerner R., Mrsny R.J., Madara J.L. (1997). Physiological regulation of epithelial tight junctions is associated with myosin light-chain phosphorylation. Am. J. Physiol..

[84] Hartmann P., Haimerl M., Mazagova M., Brenner D.A., Schnabl B. (2012). Toll-like receptor 2-mediated intestinal injury and enteric tumor necrosis factor receptor I contribute to liver fibrosis in mice. Gastroenterology.

[85] Ahmad R., Rah B., Bastola D., Dhawan P., Singh A.B. (2017). Obesity-induces Organ and Tissue Specific Tight Junction Restructuring and Barrier Deregulation by Claudin Switching. Sci. Rep..

[86] Zhang B., Yue R., Chen Y., Huang X., Yang M., Shui J., Peng Y. (2020). The Herbal Medicine Scutellaria-Coptis Alleviates Intestinal Mucosal Barrier Damage in Diabetic Rats by Inhibiting Inflammation and Modulating the Gut Microbiota. Evid. Based. Complement. Altern. Med..

[87] Nighot M., Ganapathy A.S., Saha K., Suchanec E., Castillo E., Gregory A., Shapiro S., Ma T., Nighot P. (2021). Matrix Metalloproteinase MMP-12 promotes macrophage transmigration across intestinal epithelial tight junctions and increases severity of experimental colitis. J. Crohns Colitis.

[88] Wiest R., Lawson M., Geuking M. (2014). Pathological bacterial translocation in liver cirrhosis. J. Hepatol..

[89] Cheroutre H., Lambolez F., Mucida D. (2011). The light and dark sides of intestinal intraepithelial lymphocytes. Nat. Rev. Immunol..

[90] McDonald B.D., Jabri B., Bendelac A. (2018). Diverse developmental pathways of intestinal intraepithelial lymphocytes. Nat. Rev. Immunol..

[91] Khan S., Luck H., Winer S., Winer D.A. (2021). Emerging concepts in intestinal immune control of obesity-related metabolic disease. Nat. Commun..

[92] Chieppa M., Rescigno M., Huang A.Y.C., Germain R.N. (2006). Dynamic imaging of dendritic cell extension into the small bowel lumen in response to epithelial cell TLR engagement. J. Exp. Med..

[93] Niess J.H., Brand S., Gu X., Landsman L., Jung S., McCormick B.A., Vyas J.M., Boes M., Ploegh H.L., Fox J.G. (2005). CX3CR1-mediated dendritic cell access to the intestinal lumen and bacterial clearance. Science.

[94] Mazzini E., Massimiliano L., Penna G., Rescigno M. (2014). Oral tolerance can be established via gap junction transfer of fed antigens from CX3CR1+ macrophages to CD103+ dendritic cells. Immunity.

[95] Brennan P.J., Brigl M., Brenner M.B. (2013). Invariant natural killer T cells: An innate activation scheme linked to diverse effector functions. Nat. Rev. Immunol..

[96] Dias J., Leeansyah E., Sandberg J.K. (2017). Multiple layers of heterogeneity and subset diversity in human MAIT cell responses to distinct microorganisms and to innate cytokines. Proc. Natl. Acad. Sci. USA.

[97] Corbett A.J., Eckle S.B., Birkinshaw R.W., Liu L., Patel O., Mahony J., Chen Z., Reantragoon R., Meehan B., Cao H. (2014). T-cell activation by transitory neo-antigens derived from distinct microbial pathways. Nature.

[98] Sandquist I., Kolls J. (2018). Update on regulation and effector functions of Th17 cells. F1000Research.

[99] Hirota K., Turner J.-E., Villa M., Duarte J.H., Demengeot J., Steinmetz O.M., Stockinger B. (2013). Plasticity of T H 17 cells in Peyer’s patches is responsible for the induction of T cell–dependent IgA responses. Nat. Immunol..

[100] Atarashi K., Tanoue T., Ando M., Kamada N., Nagano Y., Narushima S., Suda W., Imaoka A., Setoyama H., Nagamori T. (2015). Th17 Cell Induction by Adhesion of Microbes to Intestinal Epithelial Cells. Cell.

[101] Gaboriau-Routhiau V., Rakotobe S., Lecuyer E., Mulder I., Lan A., Bridonneau C., Rochet V., Pisi A., De Paepe M., Brandi G. (2009). The key role of segmented filamentous bacteria in the coordinated maturation of gut helper T cell responses. Immunity.

[102] Ivanov I.I., Atarashi K., Manel N., Brodie E.L., Shima T., Karaoz U., Wei D., Goldfarb K.C., Santee C.A., Lynch S.V. (2009). Induction of intestinal Th17 cells by segmented filamentous bacteria. Cell.

[103] Sharma A., Rudra D. (2018). Emerging Functions of Regulatory T Cells in Tissue Homeostasis. Front. Immunol..

[104] Wojno E.D.T., Artis D. (2016). Emerging concepts and future challenges in innate lymphoid cell biology. J. Exp. Med..

[105] Park J.-H., Eberl G. (2018). Type 3 regulatory T cells at the interface of symbiosis. J. Microbiol..

[106] Gautreaux M.D., Gelder F.B., Deitch E.A., Berg R.D. (1995). Adoptive transfer of T lymphocytes to T-cell-depleted mice inhibits Escherichia coli translocation from the gastrointestinal tract. Infect. Immun..

[107] Gautreaux M.D., Deitch E.A., Berg R.D. (1994). T lymphocytes in host defense against bacterial translocation from the gastrointestinal tract. Infect. Immun..

[108] Belkaid Y., Hand T.W. (2014). Role of the microbiota in immunity and inflammation. Cell.

[109] Spadoni I., Zagato E., Bertocchi A., Paolinelli R., Hot E., Di Sabatino A., Caprioli F., Bottiglieri L., Oldani A., Viale G. (2015). A gut-vascular barrier controls the systemic dissemination of bacteria. Science.

[110] Spadoni I., Fornasa G., Rescigno M. (2017). Organ-specific protection mediated by cooperation between vascular and epithelial barriers. Nat. Rev. Immunol..

[111] Cornet A., Savidge T.C., Cabarrocas J., Deng W.L., Colombel J.F., Lassmann H., Desreumaux P., Liblau R.S. (2001). Enterocolitis induced by autoimmune targeting of enteric glial cells: A possible mechanism in Crohn’s disease?. Proc. Natl. Acad. Sci. USA.

[112] Ciccia F., Guggino G., Rizzo A., Alessandro R., Luchetti M.M., Milling S., Saieva L., Cypers H., Stampone T., Di Benedetto P. (2017). Dysbiosis and zonulin upregulation alter gut epithelial and vascular barriers in patients with ankylosing spondylitis. Ann. Rheum. Dis..

[113] Mouries J., Brescia P., Silvestri A., Spadoni I., Sorribas M., Wiest R., Mileti E., Galbiati M., Invernizzi P., Adorini L. (2019). Microbiota-driven gut vascular barrier disruption is a prerequisite for non-alcoholic steatohepatitis development. J. Hepatol..

[114] Balmer M.L., Slack E., de Gottardi A., Lawson M.A., Hapfelmeier S., Miele L., Grieco A., Van Vlierberghe H., Fahrner R., Patuto N. (2014). The liver may act as a firewall mediating mutualism between the host and its gut commensal microbiota. Sci. Transl. Med..

[115] Wood N.J. (2014). Liver: The liver as a firewall--clearance of commensal bacteria that have escaped from the gut. Nat. Rev. Gastroenterol. Hepatol..

[116] Macpherson A.J., Harris N.L. (2004). Interactions between commensal intestinal bacteria and the immune system. Nat. Rev. Immunol..

[117] Maynard C.L., Elson C.O., Hatton R.D., Weaver C.T. (2012). Reciprocal interactions of the intestinal microbiota and immune system. Nature.

[118] Macpherson A.J., Uhr T. (2004). Induction of protective IgA by intestinal dendritic cells carrying commensal bacteria. Science.

[119] Macpherson A.J., Gatto D., Sainsbury E., Harriman G.R., Hengartner H., Zinkernagel R.M. (2000). A primitive T cell-independent mechanism of intestinal mucosal IgA responses to commensal bacteria. Science.

[120] Brun P., Castagliuolo I., Di Leo V., Buda A., Pinzani M., Palu G., Martines D. (2007). Increased intestinal permeability in obese mice: New evidence in the pathogenesis of nonalcoholic steatohepatitis. Am. J. Physiol. Gastrointest. Liver Physiol..

[121] Etienne-Mesmin L., Vijay-Kumar M., Gewirtz A.T., Chassaing B. (2016). Hepatocyte Toll-Like Receptor 5 Promotes Bacterial Clearance and Protects Mice Against High-Fat Diet-Induced Liver Disease. Cell Mol. Gastroenterol. Hepatol..

[122] Lee W.Y., Moriarty T.J., Wong C.H., Zhou H., Strieter R.M., van Rooijen N., Chaconas G., Kubes P. (2010). An intravascular immune response to Borrelia burgdorferi involves Kupffer cells and iNKT cells. Nat. Immunol..

[123] Knook D.L., Barkway C., Sleyster E.C. (1981). Lysosomal enzyme content of Kupffer and endothelial liver cells isolated from germfree and clean conventional rats. Infect. Immun..

[124] Schwabe R.F., Seki E., Brenner D.A. (2006). Toll-like receptor signaling in the liver. Gastroenterology.

[125] Fox E.S., Thomas P., Broitman S.A. (1989). Clearance of gut-derived endotoxins by the liver. Release and modification of 3H, 14C-lipopolysaccharide by isolated rat Kupffer cells. Gastroenterology.

[126] Su G.L., Klein R.D., Aminlari A., Zhang H.Y., Steinstraesser L., Alarcon W.H., Remick D.G., Wang S.C. (2000). Kupffer cell activation by lipopolysaccharide in rats: Role for lipopolysaccharide binding protein and toll-like receptor 4. Hepatology.

[127] Schumann R.R., Kirschning C.J., Unbehaun A., Aberle H.P., Knope H.P., Lamping N., Ulevitch R.J., Herrmann F. (1996). The lipopolysaccharide-binding protein is a secretory class 1 acute-phase protein whose gene is transcriptionally activated by APRF/STAT/3 and other cytokine-inducible nuclear proteins. Mol. Cell. Biol..

[128] Pugin J., Schurer-Maly C.C., Leturcq D., Moriarty A., Ulevitch R.J., Tobias P.S. (1993). Lipopolysaccharide activation of human endothelial and epithelial cells is mediated by lipopolysaccharide-binding protein and soluble CD14. Proc. Natl. Acad. Sci. USA.

[129] Landmann R., Knopf H.P., Link S., Sansano S., Schumann R., Zimmerli W. (1996). Human monocyte CD14 is upregulated by lipopolysaccharide. Infect. Immun..

[130] Frey E.A., Miller D.S., Jahr T.G., Sundan A., Bazil V., Espevik T., Finlay B.B., Wright S.D. (1992). Soluble CD14 participates in the response of cells to lipopolysaccharide. J. Exp. Med..

[131] Grover M., Camilleri M., Hines J., Burton D., Ryks M., Wadhwa A., Sundt W., Dyer R., Singh R.J. (2016). 13C mannitol as a novel biomarker for measurement of intestinal permeability. Neurogastroenterol. Motil..

[132] Camilleri M., Nadeau A., Lamsam J., Nord S.L., Ryks M., Burton D., Sweetser S., Zinsmeister A.R., Singh R. (2010). Understanding measurements of intestinal permeability in healthy humans with urine lactulose and mannitol excretion. Neurogastroenterol. Motil..

[133] Rao A.S., Camilleri M., Eckert D.J., Busciglio I., Burton D.D., Ryks M., Wong B.S., Lamsam J., Singh R., Zinsmeister A.R. (2011). Urine sugars for in vivo gut permeability: Validation and comparisons in irritable bowel syndrome-diarrhea and controls. Am. J. Physiol.-Gastrointest. Liver Physiol..

[134] Khoshbin K., Khanna L., Maselli D., Atieh J., Breen-Lyles M., Arndt K., Rhoten D., Dyer R.B., Singh R.J., Nayar S. (2021). Development and Validation of Test for “Leaky Gut” Small Intestinal and Colonic Permeability Using Sugars in Healthy Adults. Gastroenterology.

[135] Seethaler B., Basrai M., Neyrinck A.M., Nazare J.-A., Walter J., Delzenne N.M., Bischoff S.C. (2021). Biomarkers for assessment of intestinal permeability in clinical practice. Am. J. Physiol.—Gastrointest. Liver Physiol..

[136] Di Palo D.M., Garruti G., Di Ciaula A., Molina-Molina E., Shanmugam H., De Angelis M., Portincasa P. (2020). Increased Colonic Permeability and Lifestyles as Contributing Factors to Obesity and Liver Steatosis. Nutrients.

[137] Suenaert P., Bulteel V., Lemmens L., Noman M., Geypens B., Van Assche G., Geboes K., Ceuppens J.L., Rutgeerts P. (2002). Anti-tumor necrosis factor treatment restores the gut barrier in Crohn’s disease. Am. J. Gastroenterol..

[138] Cantarel B.L., Lombard V., Henrissat B. (2012). Complex carbohydrate utilization by the healthy human microbiome. PLoS ONE.

[139] Institute of Medicine (2005). Dietary, Functional, and Total Fiber. Dietary Reference Intakes for Energy, Carbohydrate, Fiber, Fat, Fatty Acids, Cholesterol, Protein, and Amino Acids.

[140] Soliman G.A. (2019). Dietary Fiber, Atherosclerosis, and Cardiovascular Disease. Nutrients.

[141] Titgemeyer E.C., Bourquin L.D., Fahey G.C., Garleb K.A. (1991). Fermentability of various fiber sources by human fecal bacteria in vitro. Am. J. Clin. Nutr..

[142] Swann O.G., Kilpatrick M., Breslin M., Oddy W.H. (2019). Dietary fiber and its associations with depression and inflammation. Nutr. Rev..

[143] Sonnenburg E.D., Smits S.A., Tikhonov M., Higginbottom S.K., Wingreen N.S., Sonnenburg J.L. (2016). Diet-induced extinctions in the gut microbiota compound over generations. Nature.

[144] Macia L., Tan J., Vieira A.T., Leach K., Stanley D., Luong S., Maruya M., Ian McKenzie C., Hijikata A., Wong C. (2015). Metabolite-sensing receptors GPR43 and GPR109A facilitate dietary fibre-induced gut homeostasis through regulation of the inflammasome. Nat. Commun..

[145] Hytting-Andreasen R., Balk-Møller E., Hartmann B., Pedersen J., Windeløv J.A., Holst J.J., Kissow H. (2018). Endogenous glucagon-like peptide- 1 and 2 are essential for regeneration after acute intestinal injury in mice. PLoS ONE.

[146] Maruta K., Takajo T., Akiba Y., Said H., Irie E., Kato I., Kuwahara A., Kaunitz J.D. (2020). GLP-2 Acutely Prevents Endotoxin-Related Increased Intestinal Paracellular Permeability in Rats. Dig. Dis. Sci..

[147] Hunt J.E., Hartmann B., Schoonjans K., Holst J.J., Kissow H. (2021). Dietary Fiber Is Essential to Maintain Intestinal Size, L-Cell Secretion, and Intestinal Integrity in Mice. Front. Endocrinol..

[148] Genda T., Sasaki Y., Kondo T., Hino S., Nishimura N., Tsukahara T., Sonoyama K., Morita T. (2017). Fructo-oligosaccharide-Induced Transient Increases in Cecal Immunoglobulin A Concentrations in Rats Are Associated with Mucosal Inflammation in Response to Increased Gut Permeability. J. Nutr..

[149] Chen T., Ma Y., Xu L., Sun C., Xu H., Zhu J. (2021). Soluble Dietary Fiber Reduces Feeding Intolerance in Severe Acute Pancreatitis: A Randomized Study. JPEN J. Parenter Enter. Nutr..

[150] Wilms E., Gerritsen J., Smidt H., Besseling-Van Der Vaart I., Rijkers G.T., Garcia Fuentes A.R., Masclee A.A.M., Troost F.J. (2016). Effects of Supplementation of the Synbiotic Ecologic^®^ 825/FOS P6 on Intestinal Barrier Function in Healthy Humans: A Randomized Controlled Trial. PLoS ONE.

[151] Drabińska N., Krupa-Kozak U., Jarocka-Cyrta E. (2020). Intestinal Permeability in Children with Celiac Disease after the Administration of Oligofructose-Enriched Inulin into a Gluten-Free Diet—Results of a Randomized, Placebo-Controlled, Pilot Trial. Nutrients.

[152] Ganda Mall J.-P., Fart F., Sabet J.A., Lindqvist C.M., Nestestog R., Hegge F.T., Keita Å.V., Brummer R.J., Schoultz I. (2020). Effects of Dietary Fibres on Acute Indomethacin-Induced Intestinal Hyperpermeability in the Elderly: A Randomised Placebo Controlled Parallel Clinical Trial. Nutrients.

[153] Schirmer M., Smeekens S.P., Vlamakis H., Jaeger M., Oosting M., Franzosa E.A., Ter Horst R., Jansen T., Jacobs L., Bonder M.J. (2016). Linking the Human Gut Microbiome to Inflammatory Cytokine Production Capacity. Cell.

[154] Rios-Covian D., Ruas-Madiedo P., Margolles A., Gueimonde M., de Los Reyes-Gavilan C.G., Salazar N. (2016). Intestinal Short Chain Fatty Acids and their Link with Diet and Human Health. Front. Microbiol..

[155] Morrison D.J., Preston T. (2016). Formation of short chain fatty acids by the gut microbiota and their impact on human metabolism. Gut Microbes.

[156] Rios-Covian D., Gueimonde M., Duncan S.H., Flint H.J., de los Reyes-Gavilan C.G. (2015). Enhanced butyrate formation by cross-feeding between Faecalibacterium prausnitzii and Bifidobacterium adolescentis. FEMS Microbiol. Lett..

[157] Mahowald M.A., Rey F.E., Seedorf H., Turnbaugh P.J., Fulton R.S., Wollam A., Shah N., Wang C., Magrini V., Wilson R.K. (2009). Characterizing a model human gut microbiota composed of members of its two dominant bacterial phyla. Proc. Natl. Acad. Sci. USA.

[158] Canfora E.E., Jocken J.W., Blaak E.E. (2015). Short-chain fatty acids in control of body weight and insulin sensitivity. Nat. Rev. Endocrinol..

[159] Chambers E.S., Viardot A., Psichas A., Morrison D.J., Murphy K.G., Zac-Varghese S.E.K., MacDougall K., Preston T., Tedford C., Finlayson G.S. (2015). Effects of targeted delivery of propionate to the human colon on appetite regulation, body weight maintenance and adiposity in overweight adults. Gut.

[160] Zambell K.L., Fitch M.D., Fleming S.E. (2003). Acetate and Butyrate Are the Major Substrates for De Novo Lipogenesis in Rat Colonic Epithelial Cells. J. Nutr..

[161] Kim C.H., Park J., Kim M. (2014). Gut microbiota-derived short-chain Fatty acids, T cells, and inflammation. Immune Netw..

[162] Usuda H., Okamoto T., Wada K. (2021). Leaky Gut: Effect of Dietary Fiber and Fats on Microbiome and Intestinal Barrier. Int. J. Mol. Sci..

[163] Feng Y., Wang Y., Wang P., Huang Y., Wang F. (2018). Short-Chain Fatty Acids Manifest Stimulative and Protective Effects on Intestinal Barrier Function Through the Inhibition of NLRP3 Inflammasome and Autophagy. Cell. Physiol. Biochem. Int. J. Exp. Cell. Physiol. Biochem. Pharmacol..

[164] Alex S., Lange K., Amolo T., Grinstead J.S., Haakonsson A.K., Szalowska E., Koppen A., Mudde K., Haenen D., Al-Lahham S. (2013). Short-chain fatty acids stimulate angiopoietin-like 4 synthesis in human colon adenocarcinoma cells by activating peroxisome proliferator-activated receptor gamma. Mol. Cell. Biol..

[165] Mathewson N.D., Jenq R., Mathew A.V., Koenigsknecht M., Hanash A., Toubai T., Oravecz-Wilson K., Wu S.R., Sun Y., Rossi C. (2016). Gut microbiome-derived metabolites modulate intestinal epithelial cell damage and mitigate graft-versus-host disease. Nat. Immunol..

[166] Huang X., Oshima T., Tomita T., Fukui H., Miwa H. (2021). Butyrate Alleviates Cytokine-Induced Barrier Dysfunction by Modifying Claudin-2 Levels. Biology.

[167] Gaudier E., Jarry A., Blottiere H.M., de Coppet P., Buisine M.P., Aubert J.P., Laboisse C., Cherbut C., Hoebler C. (2004). Butyrate specifically modulates MUC gene expression in intestinal epithelial goblet cells deprived of glucose. Am. J. Physiol. Gastrointest. Liver Physiol..

[168] Saeedi B.J., Kao D.J., Kitzenberg D.A., Dobrinskikh E., Schwisow K.D., Masterson J.C., Kendrick A.A., Kelly C.J., Bayless A.J., Kominsky D.J. (2015). HIF-dependent regulation of claudin-1 is central to intestinal epithelial tight junction integrity. Mol. Biol. Cell.

[169] Kelly C.J., Zheng L., Campbell E.L., Saeedi B., Scholz C.C., Bayless A.J., Wilson K.E., Glover L.E., Kominsky D.J., Magnuson A. (2015). Crosstalk between microbiota-derived short-chain fatty acids and intestinal epithelial HIF augments tissue barrier function. Cell Host Microbe.

[170] Vernero M., De Blasio F., Ribaldone D.G., Bugianesi E., Pellicano R., Saracco G.M., Astegiano M., Caviglia G.P. (2020). The Usefulness of Microencapsulated Sodium Butyrate Add-On Therapy in Maintaining Remission in Patients with Ulcerative Colitis: A Prospective Observational Study. J. Clin. Med..

[171] Nowarski R., Jackson R., Gagliani N., de Zoete M.R., Palm N.W., Bailis W., Low J.S., Harman C.C., Graham M., Elinav E. (2015). Epithelial IL-18 Equilibrium Controls Barrier Function in Colitis. Cell.

[172] Tong L.C., Wang Y., Wang Z.B., Liu W.Y., Sun S., Li L., Su D.F., Zhang L.C. (2016). Propionate Ameliorates Dextran Sodium Sulfate-Induced Colitis by Improving Intestinal Barrier Function and Reducing Inflammation and Oxidative Stress. Front. Pharm..

[173] Marchix J., Goddard G., Helmrath M.A. (2018). Host-Gut Microbiota Crosstalk in Intestinal Adaptation. Cell Mol. Gastroenterol. Hepatol..

[174] Musso G., Gambino R., Cassader M. (2010). Obesity, diabetes, and gut microbiota: The hygiene hypothesis expanded?. Diabetes Care.

[175] Svegliati-Baroni G., Saccomanno S., Rychlicki C., Agostinelli L., De Minicis S., Candelaresi C., Faraci G., Pacetti D., Vivarelli M., Nicolini D. (2011). Glucagon-like peptide-1 receptor activation stimulates hepatic lipid oxidation and restores hepatic signalling alteration induced by a high-fat diet in nonalcoholic steatohepatitis. Liver Int..

[176] Park J., Kim M., Kang S.G., Jannasch A.H., Cooper B., Patterson J., Kim C.H. (2015). Short-chain fatty acids induce both effector and regulatory T cells by suppression of histone deacetylases and regulation of the mTOR-S6K pathway. Mucosal. Immunol..

[177] Kim M.H., Kang S.G., Park J.H., Yanagisawa M., Kim C.H. (2013). Short-chain fatty acids activate GPR41 and GPR43 on intestinal epithelial cells to promote inflammatory responses in mice. Gastroenterology.

[178] Suzuki T., Yoshida S., Hara H. (2008). Physiological concentrations of short-chain fatty acids immediately suppress colonic epithelial permeability. Br. J. Nutr..

[179] Suligoj T., Vigsnaes L.K., Abbeele P.V.D., Apostolou A., Karalis K., Savva G.M., McConnell B., Juge N. (2020). Effects of Human Milk Oligosaccharides on the Adult Gut Microbiota and Barrier Function. Nutrients.

[180] Swanson G.R., Siskin J., Gorenz A., Shaikh M., Raeisi S., Fogg L., Forsyth C., Keshavarzian A. (2020). Disrupted diurnal oscillation of gut-derived Short chain fatty acids in shift workers drinking alcohol: Possible mechanism for loss of resiliency of intestinal barrier in disrupted circadian host. Transl. Res. J. Lab. Clin. Med..

[181] Leung C., Rivera L., Furness J.B., Angus P.W. (2016). The role of the gut microbiota in NAFLD. Nat. Rev. Gastroenterol. Hepatol..

[182] Brussow H., Parkinson S.J. (2014). You are what you eat. Nat. Biotechnol..

[183] Subramanian S., Goodspeed L., Wang S., Kim J., Zeng L., Ioannou G.N., Haigh W.G., Yeh M.M., Kowdley K.V., O’Brien K.D. (2011). Dietary cholesterol exacerbates hepatic steatosis and inflammation in obese LDL receptor-deficient mice. J. Lipid Res..

[184] Pham V.T., Calatayud M., Rotsaert C., Seifert N., Richard N., Van den Abbeele P., Marzorati M., Steinert R.E. (2021). Antioxidant Vitamins and Prebiotic FOS and XOS Differentially Shift Microbiota Composition and Function and Improve Intestinal Epithelial Barrier In Vitro. Nutrients.

[185] Salonen A., Lahti L., Salojarvi J., Holtrop G., Korpela K., Duncan S.H., Date P., Farquharson F., Johnstone A.M., Lobley G.E. (2014). Impact of diet and individual variation on intestinal microbiota composition and fermentation products in obese men. ISME J..

[186] Baothman O.A., Zamzami M.A., Taher I., Abubaker J., Abu-Farha M. (2016). The role of Gut Microbiota in the development of obesity and Diabetes. Lipids Health Dis..

[187] Walker A.W., Duncan S.H., McWilliam Leitch E.C., Child M.W., Flint H.J. (2005). pH and peptide supply can radically alter bacterial populations and short-chain fatty acid ratios within microbial communities from the human colon. Appl. Environ. Microbiol..

[188] Qin J., Li Y., Cai Z., Li S., Zhu J., Zhang F., Liang S., Zhang W., Guan Y., Shen D. (2012). A metagenome-wide association study of gut microbiota in type 2 diabetes. Nature.

[189] Zhao L., Zhang F., Ding X., Wu G., Lam Y.Y., Wang X., Fu H., Xue X., Lu C., Ma J. (2018). Gut bacteria selectively promoted by dietary fibers alleviate type 2 diabetes. Science.

[190] Singh A., Zapata R.C., Pezeshki A., Reidelberger R.D., Chelikani P.K. (2018). Inulin fiber dose-dependently modulates energy balance, glucose tolerance, gut microbiota, hormones and diet preference in high-fat-fed male rats. J. Nutr. Biochem..

[191] Vandeputte D., Falony G., Vieira-Silva S., Wang J., Sailer M., Theis S., Verbeke K., Raes J. (2017). Prebiotic inulin-type fructans induce specific changes in the human gut microbiota. Gut.

[192] Karlsson F.H., Tremaroli V., Nookaew I., Bergstrom G., Behre C.J., Fagerberg B., Nielsen J., Backhed F. (2013). Gut metagenome in European women with normal, impaired and diabetic glucose control. Nature.

[193] Zhang T., Li P., Wu X., Lu G., Marcella C., Ji X., Ji G., Zhang F. (2020). Alterations of Akkermansia muciniphila in the inflammatory bowel disease patients with washed microbiota transplantation. Appl. Microbiol. Biotechnol..

[194] Bajer L., Kverka M., Kostovcik M., Macinga P., Dvorak J., Stehlikova Z., Brezina J., Wohl P., Spicak J., Drastich P. (2017). Distinct gut microbiota profiles in patients with primary sclerosing cholangitis and ulcerative colitis. World J. Gastroenterol..

[195] Bian X., Wu W., Yang L., Lv L., Wang Q., Li Y., Ye J., Fang D., Wu J., Jiang X. (2019). Administration of Akkermansia muciniphila Ameliorates Dextran Sulfate Sodium-Induced Ulcerative Colitis in Mice. Front. Microbiol..

[196] Zhai R., Xue X., Zhang L., Yang X., Zhao L., Zhang C. (2019). Strain-Specific Anti-inflammatory Properties of Two Akkermansia muciniphila Strains on Chronic Colitis in Mice. Front. Cell. Infect. Microbiol..

[197] Wang L., Tang L., Feng Y., Zhao S., Han M., Zhang C., Yuan G., Zhu J., Cao S., Wu Q. (2020). A purified membrane protein from Akkermansia muciniphila or the pasteurised bacterium blunts colitis associated tumourigenesis by modulation of CD8(+) T cells in mice. Gut.

[198] Ottman N., Reunanen J., Meijerink M., Pietila T.E., Kainulainen V., Klievink J., Huuskonen L., Aalvink S., Skurnik M., Boeren S. (2017). Pili-like proteins of Akkermansia muciniphila modulate host immune responses and gut barrier function. PLoS ONE.

[199] Guglielmetti S., Bernardi S., Del Bo C., Cherubini A., Porrini M., Gargari G., Hidalgo-Liberona N., Gonzalez-Dominguez R., Peron G., Zamora-Ros R. (2020). Effect of a polyphenol-rich dietary pattern on intestinal permeability and gut and blood microbiomics in older subjects: Study protocol of the MaPLE randomised controlled trial. BMC Geriatr..

[200] Wang P., Wang J., Li D., Ke W., Chen F., Hu X. (2020). Targeting the gut microbiota with resveratrol: A demonstration of novel evidence for the management of hepatic steatosis. J. Nutr. Biochem..

[201] Fan J., Zhao X.H., Li T.J. (2021). Heat treatment of galangin and kaempferol inhibits their benefits to improve barrier function in rat intestinal epithelial cells. J. Nutr. Biochem..

[202] Carrasco-Pozo C., Morales P., Gotteland M. (2013). Polyphenols Protect the Epithelial Barrier Function of Caco-2 Cells Exposed to Indomethacin through the Modulation of Occludin and Zonula Occludens-1 Expression. J. Agric. Food Chem..

[203] Suzuki T., Hara H. (2009). Quercetin Enhances Intestinal Barrier Function through the Assembly of Zonnula Occludens-2, Occludin, and Claudin-1 and the Expression of Claudin-4 in Caco-2 Cells. J. Nutr..

[204] Cremonini E., Daveri E., Mastaloudis A., Adamo A.M., Mills D., Kalanetra K., Hester S.N., Wood S.M., Fraga C.G., Oteiza P.I. (2019). Anthocyanins protect the gastrointestinal tract from high fat diet-induced alterations in redox signaling, barrier integrity and dysbiosis. Redox Biol..

[205] Lyall K.A., Hurst S.M., Cooney J., Jensen D., Lo K., Hurst R.D., Stevenson L.M. (2009). Short-term blackcurrant extract consumption modulates exercise-induced oxidative stress and lipopolysaccharide-stimulated inflammatory responses. Am. J. Physiol. Regul. Integr. Comp. Physiol..

[206] Smeriglio A., Barreca D., Bellocco E., Trombetta D. (2017). Proanthocyanidins and hydrolysable tannins: Occurrence, dietary intake and pharmacological effects. Br. J. Pharm..

[207] Zhao R., Long X., Yang J., Du L., Zhang X., Li J., Hou C. (2019). Pomegranate peel polyphenols reduce chronic low-grade inflammatory responses by modulating gut microbiota and decreasing colonic tissue damage in rats fed a high-fat diet. Food Funct..

[208] Hering N.A., Luettig J., Jebautzke B., Schulzke J.D., Rosenthal R. (2021). The Punicalagin Metabolites Ellagic Acid and Urolithin A Exert Different Strengthening and Anti-Inflammatory Effects on Tight Junction-Mediated Intestinal Barrier Function In Vitro. Front. Pharm..

[209] Zhou Q., Verne M.L., Fields J.Z., Lefante J.J., Basra S., Salameh H., Verne G.N. (2019). Randomised placebo-controlled trial of dietary glutamine supplements for postinfectious irritable bowel syndrome. Gut.

[210] Benjamin J., Makharia G., Ahuja V., Anand Rajan K.D., Kalaivani M., Gupta S.D., Joshi Y.K. (2012). Glutamine and whey protein improve intestinal permeability and morphology in patients with Crohn’s disease: A randomized controlled trial. Dig. Dis. Sci..

[211] Anderson P.M., Lalla R.V. (2020). Glutamine for Amelioration of Radiation and Chemotherapy Associated Mucositis during Cancer Therapy. Nutrients.

[212] Linsalata M., Riezzo G., Orlando A., D’Attoma B., Prospero L., Tutino V., Notarnicola M., Russo F. (2021). The Relationship between Low Serum Vitamin D Levels and Altered Intestinal Barrier Function in Patients with IBS Diarrhoea Undergoing a Long-Term Low-FODMAP Diet: Novel Observations from a Clinical Trial. Nutrients.

[213] Raftery T., Martineau A.R., Greiller C.L., Ghosh S., McNamara D., Bennett K., Meddings J., O’Sullivan M. (2015). Effects of vitamin D supplementation on intestinal permeability, cathelicidin and disease markers in Crohn’s disease: Results from a randomised double-blind placebo-controlled study. United Eur. Gastroenterol. J..

[214] Mahmood A., FitzGerald A.J., Marchbank T., Ntatsaki E., Murray D., Ghosh S., Playford R.J. (2007). Zinc carnosine, a health food supplement that stabilises small bowel integrity and stimulates gut repair processes. Gut.

[215] Camilleri M. (2021). Human Intestinal Barrier: Effects of Stressors, Diet, Prebiotics, and Probiotics. Clin. Transl. Gastroenterol..

[216] Di Ciaula A., Passarella S., Shanmugam H., Noviello M., Bonfrate L., Wang D.Q.-H., Portincasa P. (2021). Nonalcoholic Fatty Liver Disease (NAFLD). Mitochondria as Players and Targets of Therapies?. Int. J. Mol. Sci..

[217] Donnelly K.L., Smith C.I., Schwarzenberg S.J., Jessurun J., Boldt M.D., Parks E.J. (2005). Sources of fatty acids stored in liver and secreted via lipoproteins in patients with nonalcoholic fatty liver disease. J. Clin. Investig..

[218] Usami M., Komurasaki T., Hanada A., Kinoshita K., Ohata A. (2003). Effect of γ-linolenic acid or docosahexaenoic acid on tight junction permeability in intestinal monolayer cells and their mechanism by protein kinase C activation and/or eicosanoid formation. Nutrition.

[219] Usami M., Muraki K., Iwamoto M., Ohata A., Matsushita E., Miki A. (2001). Effect of eicosapentaenoic acid (EPA) on tight junction permeability in intestinal monolayer cells. Clin. Nutr..

[220] Willemsen L.E., Koetsier M.A., Balvers M., Beermann C., Stahl B., van Tol E.A. (2008). Polyunsaturated fatty acids support epithelial barrier integrity and reduce IL-4 mediated permeability in vitro. Eur. J. Nutr..

[221] Lindmark T., Nikkila T., Artursson P. (1995). Mechanisms of absorption enhancement by medium chain fatty acids in intestinal epithelial Caco-2 cell monolayers. J. Pharmacol. Exp. Ther..

[222] Anderberg E.K., Lindmark T., Artursson P. (1993). Sodium caprate elicits dilatations in human intestinal tight junctions and enhances drug absorption by the paracellular route. Pharm. Res..

[223] De La Serre C.B., Ellis C.L., Lee J., Hartman A.L., Rutledge J.C., Raybould H.E. (2010). Propensity to high-fat diet-induced obesity in rats is associated with changes in the gut microbiota and gut inflammation. Am. J. Physiol. Gastrointest. Liver Physiol..

[224] Tian B., Zhao J., Zhang M., Chen Z., Ma Q., Liu H., Nie C., Zhang Z., An W., Li J. (2021). Lycium ruthenicum Anthocyanins Attenuate High-Fat Diet-Induced Colonic Barrier Dysfunction and Inflammation in Mice by Modulating the Gut Microbiota. Mol. Nutr. Food Res..

[225] Mujawdiya P.K., Sharma P., Sharad S., Kapur S. (2020). Reversal of Increase in Intestinal Permeability by Mangifera indica Seed Kernel Extract in High-Fat Diet-Induced Obese Mice. Pharmaceuticals.

[226] Nascimento J.C., Matheus V.A., Oliveira R.B., Tada S.F.S., Collares-Buzato C.B. (2020). High-Fat Diet Induces Disruption of the Tight Junction-Mediated Paracellular Barrier in the Proximal Small Intestine Before the Onset of Type 2 Diabetes and Endotoxemia. Dig. Dis. Sci..

[227] Zhao J., Wang H., Yang H., Zhou Y., Tang L. (2020). Autophagy induction by rapamycin ameliorates experimental colitis and improves intestinal epithelial barrier function in IL-10 knockout mice. Int. Immunopharmacol..

[228] Devkota S., Wang Y., Musch M.W., Leone V., Fehlner-Peach H., Nadimpalli A., Antonopoulos D.A., Jabri B., Chang E.B. (2012). Dietary-fat-induced taurocholic acid promotes pathobiont expansion and colitis in Il10−/− mice. Nature.

[229] Agus A., Denizot J., Thevenot J., Martinez-Medina M., Massier S., Sauvanet P., Bernalier-Donadille A., Denis S., Hofman P., Bonnet R. (2016). Western diet induces a shift in microbiota composition enhancing susceptibility to Adherent-Invasive *E. coli* infection and intestinal inflammation. Sci. Rep..

[230] Muhomah T.A., Nishino N., Katsumata E., Haoming W., Tsuruta T. (2019). High-fat diet reduces the level of secretory immunoglobulin A coating of commensal gut microbiota. Biosci Microbiota Food Health.

[231] John S., Luben R., Shrestha S.S., Welch A., Khaw K.T., Hart A.R. (2010). Dietary n-3 polyunsaturated fatty acids and the aetiology of ulcerative colitis: A UK prospective cohort study. Eur. J. Gastroenterol. Hepatol..

[232] Schreiner P., Martinho-Grueber M., Studerus D., Vavricka S.R., Tilg H., Biedermann L., on behalf of Swiss IBDnet, an official working group of the Swiss Society of Gastroenterology (2020). Nutrition in Inflammatory Bowel Disease. Digestion.

[233] Patterson E., Wall R., Fitzgerald G.F., Ross R.P., Stanton C. (2012). Health implications of high dietary omega-6 polyunsaturated Fatty acids. J. Nutr. Metab..

[234] Chapkin R.S., Davidson L.A., Ly L., Weeks B.R., Lupton J.R., McMurray D.N. (2007). Immunomodulatory effects of (n-3) fatty acids: Putative link to inflammation and colon cancer. J. Nutr..

[235] Cani P.D., Amar J., Iglesias M.A., Poggi M., Knauf C., Bastelica D., Neyrinck A.M., Fava F., Tuohy K.M., Chabo C. (2007). Metabolic endotoxemia initiates obesity and insulin resistance. Diabetes.

[236] Amar J., Burcelin R., Ruidavets J.B., Cani P.D., Fauvel J., Alessi M.C., Chamontin B., Ferrieres J. (2008). Energy intake is associated with endotoxemia in apparently healthy men. Am. J. Clin. Nutr..

[237] Lyte J.M., Gabler N.K., Hollis J.H. (2016). Postprandial serum endotoxin in healthy humans is modulated by dietary fat in a randomized, controlled, cross-over study. Lipids Health Dis..

[238] Bowser S.M., McMillan R.P., Boutagy N.E., Tarpey M.D., Smithson A.T., Osterberg K.L., Neilson A.P., Davy B.M., Davy K.P., Hulver M.W. (2020). Serum endotoxin, gut permeability and skeletal muscle metabolic adaptations following a short term high fat diet in humans. Metab. Clin. Exp..

[239] Yang Y., Zhong Z., Wang B., Xia X., Yao W., Huang L., Wang Y., Ding W. (2019). Early-life high-fat diet-induced obesity programs hippocampal development and cognitive functions via regulation of gut commensal Akkermansia muciniphila. Neuropsychopharmacol. Off. Publ. Am. Coll. Neuropsychopharmacol..

[240] Lassenius M.I., Pietilainen K.H., Kaartinen K., Pussinen P.J., Syrjanen J., Forsblom C., Porsti I., Rissanen A., Kaprio J., Mustonen J. (2011). Bacterial endotoxin activity in human serum is associated with dyslipidemia, insulin resistance, obesity, and chronic inflammation. Diabetes Care.

[241] Shi H., Kokoeva M.V., Inouye K., Tzameli I., Yin H., Flier J.S. (2006). TLR4 links innate immunity and fatty acid-induced insulin resistance. J. Clin. Investig..

[242] Jia L., Vianna C.R., Fukuda M., Berglund E.D., Liu C., Tao C., Sun K., Liu T., Harper M.J., Lee C.E. (2014). Hepatocyte Toll-like receptor 4 regulates obesity-induced inflammation and insulin resistance. Nat. Commun..

[243] Lee J.J., Wang P.W., Yang I.H., Huang H.M., Chang C.S., Wu C.L., Chuang J.H. (2015). High-fat diet induces toll-like receptor 4-dependent macrophage/microglial cell activation and retinal impairment. Investig. Ophthalmol. Vis. Sci..

[244] Ding Y., Subramanian S., Montes V.N., Goodspeed L., Wang S., Han C., Teresa A.S., Kim J., O’Brien K.D., Chait A. (2012). Toll-like receptor 4 deficiency decreases atherosclerosis but does not protect against inflammation in obese low-density lipoprotein receptor-deficient mice. Arterioscler. Thromb. Vasc. Biol..

[245] Cheng C., Tan J., Qian W., Zhang L., Hou X. (2018). Gut inflammation exacerbates hepatic injury in the high-fat diet induced NAFLD mouse: Attention to the gut-vascular barrier dysfunction. Life Sci..

[246] Pendyala S., Walker J.M., Holt P.R. (2012). A high-fat diet is associated with endotoxemia that originates from the gut. Gastroenterology.

[247] Ghanim H., Abuaysheh S., Sia C.L., Korzeniewski K., Chaudhuri A., Fernandez-Real J.M., Dandona P. (2009). Increase in plasma endotoxin concentrations and the expression of Toll-like receptors and suppressor of cytokine signaling-3 in mononuclear cells after a high-fat, high-carbohydrate meal: Implications for insulin resistance. Diabetes Care.

[248] Park J.H., Jeong S.Y., Choi A.J., Kim S.J. (2015). Lipopolysaccharide directly stimulates Th17 differentiation in vitro modulating phosphorylation of RelB and NF-kappaB1. Immunol. Lett..

[249] Shen T., Chen X., Li Y., Tang X., Jiang X., Yu C., Zheng Y., Guo H., Ling W. (2017). Interleukin-17A exacerbates high-fat diet-induced hepatic steatosis by inhibiting fatty acid beta-oxidation. Biochim. Biophys. Acta Mol. Basis Dis..

[250] Hassan A.M., Mancano G., Kashofer K., Frohlich E.E., Matak A., Mayerhofer R., Reichmann F., Olivares M., Neyrinck A.M., Delzenne N.M. (2019). High-fat diet induces depression-like behaviour in mice associated with changes in microbiome, neuropeptide Y, and brain metabolome. Nutr. Neurosci..

[251] Munch N.S., Fang H.Y., Ingermann J., Maurer H.C., Anand A., Kellner V., Sahm V., Wiethaler M., Baumeister T., Wein F. (2019). High-Fat Diet Accelerates Carcinogenesis in a Mouse Model of Barrett’s Esophagus via Interleukin 8 and Alterations to the Gut Microbiome. Gastroenterology.

[252] Fujisaka S., Avila-Pacheco J., Soto M., Kostic A., Dreyfuss J.M., Pan H., Ussar S., Altindis E., Li N., Bry L. (2018). Diet, Genetics, and the Gut Microbiome Drive Dynamic Changes in Plasma Metabolites. Cell Rep..

[253] Sun L., Xie C., Wang G., Wu Y., Wu Q., Wang X., Liu J., Deng Y., Xia J., Chen B. (2018). Gut microbiota and intestinal FXR mediate the clinical benefits of metformin. Nat. Med..

[254] Bisanz J.E., Upadhyay V., Turnbaugh J.A., Ly K., Turnbaugh P.J. (2019). Meta-Analysis Reveals Reproducible Gut Microbiome Alterations in Response to a High-Fat Diet. Cell Host Microbe.

[255] Wei L., Yue F., Xing L., Wu S., Shi Y., Li J., Xiang X., Lam S.M., Shui G., Russell R. (2020). Constant Light Exposure Alters Gut Microbiota and Promotes the Progression of Steatohepatitis in High Fat Diet Rats. Front. Microbiol..

[256] Ashrafian F., Shahriary A., Behrouzi A., Moradi H.R., Keshavarz Azizi Raftar S., Lari A., Hadifar S., Yaghoubfar R., Ahmadi Badi S., Khatami S. (2019). Akkermansia muciniphila-Derived Extracellular Vesicles as a Mucosal Delivery Vector for Amelioration of Obesity in Mice. Front. Microbiol..

[257] Chelakkot C., Choi Y., Kim D.K., Park H.T., Ghim J., Kwon Y., Jeon J., Kim M.S., Jee Y.K., Gho Y.S. (2018). Akkermansia muciniphila-derived extracellular vesicles influence gut permeability through the regulation of tight junctions. Exp. Mol. Med..

[258] Plovier H., Everard A., Druart C., Depommier C., Van Hul M., Geurts L., Chilloux J., Ottman N., Duparc T., Lichtenstein L. (2017). A purified membrane protein from Akkermansia muciniphila or the pasteurized bacterium improves metabolism in obese and diabetic mice. Nat. Med..

[259] Zeng H., Umar S., Rust B., Lazarova D., Bordonaro M. (2019). Secondary Bile Acids and Short Chain Fatty Acids in the Colon: A Focus on Colonic Microbiome, Cell Proliferation, Inflammation, and Cancer. Int. J. Mol. Sci..

[260] Murakami Y., Tanabe S., Suzuki T. (2016). High-fat Diet-induced Intestinal Hyperpermeability is Associated with Increased Bile Acids in the Large Intestine of Mice. J. Food Sci..

[261] Chiang J.Y., Pathak P., Liu H., Donepudi A., Ferrell J., Boehme S. (2017). Intestinal Farnesoid X Receptor and Takeda G Protein Couple Receptor 5 Signaling in Metabolic Regulation. Dig. Dis..

[262] Cipriani S., Mencarelli A., Chini M.G., Distrutti E., Renga B., Bifulco G., Baldelli F., Donini A., Fiorucci S. (2011). The bile acid receptor GPBAR-1 (TGR5) modulates integrity of intestinal barrier and immune response to experimental colitis. PLoS ONE.

[263] Biagioli M., Carino A., Cipriani S., Francisci D., Marchiano S., Scarpelli P., Sorcini D., Zampella A., Fiorucci S. (2017). The Bile Acid Receptor GPBAR1 Regulates the M1/M2 Phenotype of Intestinal Macrophages and Activation of GPBAR1 Rescues Mice from Murine Colitis. J. Immunol..

[264] Inagaki T., Moschetta A., Lee Y.K., Peng L., Zhao G., Downes M., Yu R.T., Shelton J.M., Richardson J.A., Repa J.J. (2006). Regulation of antibacterial defense in the small intestine by the nuclear bile acid receptor. Proc. Natl. Acad. Sci. USA.

[265] Gadaleta R.M., van Erpecum K.J., Oldenburg B., Willemsen E.C., Renooij W., Murzilli S., Klomp L.W., Siersema P.D., Schipper M.E., Danese S. (2011). Farnesoid X receptor activation inhibits inflammation and preserves the intestinal barrier in inflammatory bowel disease. Gut.

[266] Huang M., Kong B., Zhang M., Rizzolo D., Armstrong L.E., Schumacher J.D., Chow M.D., Lee Y.H., Joseph L.B., Stofan M. (2020). Enhanced alcoholic liver disease in mice with intestine-specific farnesoid X receptor deficiency. Lab. Invest..

[267] Glade M.J., Meguid M.M. (2016). A glance at... dietary emulsifiers, the human intestinal mucus and microbiome, and dietary fiber. Nutrition.

[268] Lock J.Y., Carlson T.L., Wang C.M., Chen A., Carrier R.L. (2018). Acute Exposure to Commonly Ingested Emulsifiers Alters Intestinal Mucus Structure and Transport Properties. Sci. Rep..

[269] Svolos V., Hansen R., Nichols B., Quince C., Ijaz U.Z., Papadopoulou R.T., Edwards C.A., Watson D., Alghamdi A., Brejnrod A. (2019). Treatment of active Crohn’s disease with an ordinary food-based diet that replicates exclusive enteral nutrition. Gastroenterology.

[270] Levine A., Wine E., Assa A., Sigall Boneh R., Shaoul R., Kori M., Cohen S., Peleg S., Shamaly H., On A. (2019). Crohn’s Disease Exclusion Diet Plus Partial Enteral Nutrition Induces Sustained Remission in a Randomized Controlled Trial. Gastroenterology.

[271] Sigall-Boneh R., Pfeffer-Gik T., Segal I., Zangen T., Boaz M., Levine A. (2014). Partial enteral nutrition with a Crohn’s disease exclusion diet is effective for induction of remission in children and young adults with Crohn’s disease. Inflamm. Bowel. Dis..

[272] Sandall A.M., Cox S.R., Lindsay J.O., Gewirtz A.T., Chassaing B., Rossi M., Whelan K. (2020). Emulsifiers Impact Colonic Length in Mice and Emulsifier Restriction is Feasible in People with Crohn’s Disease. Nutrients.

[273] Bhattacharyya S., Shumard T., Xie H., Dodda A., Varady K.A., Feferman L., Halline A.G., Goldstein J.L., Hanauer S.B., Tobacman J.K. (2017). A randomized trial of the effects of the no-carrageenan diet on ulcerative colitis disease activity. Nutr. Healthy Aging.

[274] Starkel P., Leclercq S., de Timary P., Schnabl B. (2018). Intestinal dysbiosis and permeability: The yin and yang in alcohol dependence and alcoholic liver disease. Clin. Sci..

[275] Palasciano G., Portincasa P., Di Ciaula A., Palmieri V. (1995). Prolonged consumption of moderate doses of alcohol and in vitro gastro-duodenal and ileal contractility in the rat. Eur. J. Clin. Investig..

[276] Di Ciaula A., Grattagliano I., Portincasa P. (2016). Chronic alcoholics retain dyspeptic symptoms, pan-enteric dysmotility, and autonomic neuropathy before and after abstinence. J. Dig. Dis..

[277] Wang Y., Tong J., Chang B., Wang B., Zhang D., Wang B. (2014). Effects of alcohol on intestinal epithelial barrier permeability and expression of tight junction-associated proteins. Mol. Med. Rep..

[278] Yan A.W., Fouts D.E., Brandl J., Starkel P., Torralba M., Schott E., Tsukamoto H., Nelson K.E., Brenner D.A., Schnabl B. (2011). Enteric dysbiosis associated with a mouse model of alcoholic liver disease. Hepatology.

[279] Gottfried E.B., Korsten M.A., Lieber C.S. (1978). Alcohol-induced gastric and duodenal lesions in man. Am. J. Gastroenterol..

[280] Brozinsky S., Fani K., Grosberg S.J., Wapnick S. (1978). Alcohol ingestion-induced changes in the human rectal mucosa: Light and electron microscopic studies. Dis. Colon. Rectum..

[281] Elamin E., Masclee A., Troost F., Pieters H.-J., Keszthelyi D., Aleksa K., Dekker J., Jonkers D. (2014). Ethanol impairs intestinal barrier function in humans through mitogen activated protein kinase signaling: A combined in vivo and in vitro approach. PLoS ONE.

[282] Tang Y., Banan A., Forsyth C.B., Fields J.Z., Lau C.K., Zhang L.J., Keshavarzian A. (2008). Effect of alcohol on miR-212 expression in intestinal epithelial cells and its potential role in alcoholic liver disease. Alcohol. Clin. Exp. Res..

[283] Lang S., Duan Y., Liu J., Torralba M.G., Kuelbs C., Ventura-Cots M., Abraldes J.G., Bosques-Padilla F., Verna E.C., Brown R.S. (2020). Intestinal Fungal Dysbiosis and Systemic Immune Response to Fungi in Patients With Alcoholic Hepatitis. Hepatology.

[284] Parlesak A., Schafer C., Schutz T., Bode J.C., Bode C. (2000). Increased intestinal permeability to macromolecules and endotoxemia in patients with chronic alcohol abuse in different stages of alcohol-induced liver disease. J. Hepatol..

[285] Keshavarzian A., Holmes E.W., Patel M., Iber F., Fields J.Z., Pethkar S. (1999). Leaky gut in alcoholic cirrhosis: A possible mechanism for alcohol-induced liver damage. Am. J. Gastroenterol..

[286] Bjarnason I., Peters T.J., Wise R.J. (1984). The leaky gut of alcoholism: Possible route of entry for toxic compounds. Lancet.

[287] González-Muniesa P., Mártinez-González M.A., Hu F.B., Després J.P., Matsuzawa Y., Loos R.J.F., Moreno L.A., Bray G.A., Martinez J.A. (2017). Obesity. Nat. Rev. Dis. Primers.

[288] NCD Risk Factor Collaboration (NCD-RisC) (2017). Worldwide trends in body-mass index, underweight, overweight, and obesity from 1975 to 2016: A pooled analysis of 2416 population-based measurement studies in 128.9 million children, adolescents, and adults. Lancet.

[289] Collaboration N.C.D.R.F. (2016). Trends in adult body-mass index in 200 countries from 1975 to 2014: A pooled analysis of 1698 population-based measurement studies with 19.2 million participants. Lancet.

[290] The Global Burden of Metabolic Risk Factors for Chronic Diseases Collaboration (2013). Metabolic mediators of the effects of body-mass index, overweight, and obesity on coronary heart disease and stroke: A pooled analysis of 97 prospective cohorts with 1.8 million participants. Lancet.

[291] Faienza M.F., Chiarito M., Molina-Molina E., Shanmugam H., Lammert F., Krawczyk M., D’Amato G., Portincasa P. (2020). Childhood obesity, cardiovascular and liver health: A growing epidemic with age. World J. Pediatrics.

[292] Faienza M.F., Wang D.Q.H., Frühbeck G., Garruti G., Portincasa P. (2016). The dangerous link between childhood and adulthood predictors of obesity and metabolic syndrome. Intern. Emerg. Med..

[293] Bhaskaran K., Douglas I., Forbes H., dos-Santos-Silva I., Leon D.A., Smeeth L. (2014). Body-mass index and risk of 22 specific cancers: A population-based cohort study of 5.24 million UK adults. Lancet.

[294] De Meyts P., Delzenne N. (2021). Editorial: The Brain—Gut—Microbiome Network in Metabolic Regulation and Dysregulation. Front. Endocrinol..

[295] Centers for Disease Control and Prevention Overweight and Obesity: Adult Obesity Facts. https://www.cdc.gov/obesity/data/adult.html.

[296] Hales C.M., Carroll M.D., Fryar C.D., Ogden C.L. (2020). Prevalence of Obesity and Severe Obesity Among Adults: United States, 2017–2018. NCHS Data Brief..

[297] Collaborators G.B.D.O., Afshin A., Forouzanfar M.H., Reitsma M.B., Sur P., Estep K., Lee A., Marczak L., Mokdad A.H., Moradi-Lakeh M. (2017). Health Effects of Overweight and Obesity in 195 Countries over 25 Years. New Engl. J. Med..

[298] Vecchie A., Dallegri F., Carbone F., Bonaventura A., Liberale L., Portincasa P., Fruhbeck G., Montecucco F. (2018). Obesity phenotypes and their paradoxical association with cardiovascular diseases. Eur. J. Intern. Med..

[299] Baldini F., Fabbri R., Eberhagen C., Voci A., Portincasa P., Zischka H., Vergani L. (2021). Adipocyte hypertrophy parallels alterations of mitochondrial status in a cell model for adipose tissue dysfunction in obesity. Life Sci..

[300] Grattagliano I., Di Ciaula A., Baj J., Molina-Molina E., Shanmugam H., Garruti G., Wang D.Q., Portincasa P. (2021). Protocols for Mitochondria as the Target of Pharmacological Therapy in the Context of Nonalcoholic Fatty Liver Disease (NAFLD). Methods Mol. Biol..

[301] Grattagliano I., Montezinho L.P., Oliveira P.J., Fruhbeck G., Gomez-Ambrosi J., Montecucco F., Carbone F., Wieckowski M.R., Wang D.Q., Portincasa P. (2019). Targeting mitochondria to oppose the progression of nonalcoholic fatty liver disease. Biochem. Pharmacol..

[302] Grasselli E., Baldini F., Vecchione G., Oliveira P.J., Sardao V.A., Voci A., Portincasa P., Vergani L. (2019). Excess fructose and fatty acids trigger a model of nonalcoholic fatty liver disease progression in vitro: Protective effect of the flavonoid silybin. Int. J. Mol. Med..

[303] European Association for the Study of the Liver (EASL), European Association for the Study of Diabetes (EASD), European Association for the Study of Obesity (EASO) (2016). EASL-EASD-EASO Clinical Practice Guidelines for the management of non-alcoholic fatty liver disease. J. Hepatol..

[304] Cohen J.C., Horton J.D., Hobbs H.H. (2011). Human fatty liver disease: Old questions and new insights. Science.

[305] Szczepaniak L.S., Nurenberg P., Leonard D., Browning J.D., Reingold J.S., Grundy S., Hobbs H.H., Dobbins R.L. (2005). Magnetic resonance spectroscopy to measure hepatic triglyceride content: Prevalence of hepatic steatosis in the general population. Am. J. Physiol. Endocrinol. Metab..

[306] Chalasani N., Younossi Z., Lavine J.E., Charlton M., Cusi K., Rinella M., Harrison S.A., Brunt E.M., Sanyal A.J. (2018). The diagnosis and management of nonalcoholic fatty liver disease: Practice guidance from the American Association for the Study of Liver Diseases. Hepatology.

[307] Maurice J., Manousou P. (2018). Non-alcoholic fatty liver disease. Clin. Med..

[308] Wong T., Dang K., Ladhani S., Singal A.K., Wong R.J. (2019). Prevalence of Alcoholic Fatty Liver Disease Among Adults in the United States, 2001–2016. JAMA.

[309] Li J.F., Qu F., Zheng S.J., Wu H.L., Liu M., Liu S., Ren Y., Ren F., Chen Y., Duan Z.P. (2014). Elevated plasma sphingomyelin (d18:1/22:0) is closely related to hepatic steatosis in patients with chronic hepatitis C virus infection. Eur. J. Clin. Microbiol. Infect. Dis..

[310] Yasui K., Harano Y., Mitsuyoshi H., Tsuji K., Endo M., Nakajima T., Minami M., Itoh Y., Zen Y., Nakanuma Y. (2010). Steatosis and hepatic expression of genes regulating lipid metabolism in Japanese patients infected with hepatitis C virus. J. Gastroenterol..

[311] Jian Wu Y., Shu Chen L., Gui Qiang W. (2006). Effects of fatty liver and related factors on the efficacy of combination antiviral therapy in patients with chronic hepatitis C. Liver Int..

[312] Hwang S.J., Luo J.C., Chu C.W., Lai C.R., Lu C.L., Tsay S.H., Wu J.C., Chang F.Y., Lee S.D. (2001). Hepatic steatosis in chronic hepatitis C virus infection: Prevalence and clinical correlation. J. Gastroenterol. Hepatol..

[313] Safar Zadeh E., Lungu A.O., Cochran E.K., Brown R.J., Ghany M.G., Heller T., Kleiner D.E., Gorden P. (2013). The liver diseases of lipodystrophy: The long-term effect of leptin treatment. J. Hepatol..

[314] Stattermayer A.F., Traussnigg S., Dienes H.P., Aigner E., Stauber R., Lackner K., Hofer H., Stift J., Wrba F., Stadlmayr A. (2015). Hepatic steatosis in Wilson disease--Role of copper and PNPLA3 mutations. J. Hepatol..

[315] Jordan T., Popovic P., Rotovnik Kozjek N. (2020). Liver steatosis in adult patients on home parenteral nutrition. Eur. J. Clin. Nutr..

[316] Satapathy S.K., Kuwajima V., Nadelson J., Atiq O., Sanyal A.J. (2015). Drug-induced fatty liver disease: An overview of pathogenesis and management. Ann. Hepatol..

[317] Liu J., Ghaziani T.T., Wolf J.L. (2017). Acute Fatty Liver Disease of Pregnancy: Updates in Pathogenesis, Diagnosis, and Management. Am. J. Gastroenterol..

[318] Chapman J., Arnold J.K. (2021). Reye Syndrome.

[319] Soullane S., Lee G.E., Auger N. (2021). Perinatal Risk Factors for Pediatric Nonalcoholic Fatty Liver Disease: Impact of Inborn Errors of Metabolism. Clin. Gastroenterol. Hepatol..

[320] Powell E.E., Wong V.W., Rinella M. (2021). Non-alcoholic fatty liver disease. Lancet.

[321] Singh S., Allen A.M., Wang Z., Prokop L.J., Murad M.H., Loomba R. (2015). Fibrosis progression in nonalcoholic fatty liver vs nonalcoholic steatohepatitis: A systematic review and meta-analysis of paired-biopsy studies. Clin. Gastroenterol. Hepatol..

[322] Ludwig J., Viggiano T.R., McGill D.B., Oh B.J. (1980). Nonalcoholic steatohepatitis: Mayo Clinic experiences with a hitherto unnamed disease. Mayo Clin. Proc. Mayo Clin..

[323] Caldwell S.H., Oelsner D.H., Iezzoni J.C., Hespenheide E.E., Battle E.H., Driscoll C.J. (1999). Cryptogenic cirrhosis: Clinical characterization and risk factors for underlying disease. Hepatology.

[324] Browning J.D., Kumar K.S., Saboorian M.H., Thiele D.L. (2004). Ethnic differences in the prevalence of cryptogenic cirrhosis. Am. J. Gastroenterol..

[325] Nasr P., Ignatova S., Kechagias S., Ekstedt M. (2018). Natural history of nonalcoholic fatty liver disease: A prospective follow-up study with serial biopsies. Hepatol. Commun..

[326] Younossi Z., Anstee Q.M., Marietti M., Hardy T., Henry L., Eslam M., George J., Bugianesi E. (2018). Global burden of NAFLD and NASH: Trends, predictions, risk factors and prevention. Nat. Rev. Gastroenterol. Hepatol..

[327] Mittal S., El-Serag H.B., Sada Y.H., Kanwal F., Duan Z., Temple S., May S.B., Kramer J.R., Richardson P.A., Davila J.A. (2016). Hepatocellular Carcinoma in the Absence of Cirrhosis in United States Veterans is Associated With Nonalcoholic Fatty Liver Disease. Clin. Gastroenterol. Hepatol..

[328] Dulai P.S., Singh S., Patel J., Soni M., Prokop L.J., Younossi Z., Sebastiani G., Ekstedt M., Hagstrom H., Nasr P. (2017). Increased risk of mortality by fibrosis stage in nonalcoholic fatty liver disease: Systematic review and meta-analysis. Hepatology.

[329] Torbenson M.S., Yeh M.M. (2021). Steatohepatitic hepatocellular carcinoma. Hepatoma Res..

[330] Williams C.D., Stengel J., Asike M.I., Torres D.M., Shaw J., Contreras M., Landt C.L., Harrison S.A. (2011). Prevalence of nonalcoholic fatty liver disease and nonalcoholic steatohepatitis among a largely middle-aged population utilizing ultrasound and liver biopsy: A prospective study. Gastroenterology.

[331] Vernon G., Baranova A., Younossi Z.M. (2011). Systematic review: The epidemiology and natural history of non-alcoholic fatty liver disease and non-alcoholic steatohepatitis in adults. Aliment. Pharmacol. Ther..

[332] Lazo M., Hernaez R., Eberhardt M.S., Bonekamp S., Kamel I., Guallar E., Koteish A., Brancati F.L., Clark J.M. (2013). Prevalence of nonalcoholic fatty liver disease in the United States: The Third National Health and Nutrition Examination Survey, 1988-1994. Am. J. Epidemiol..

[333] Younossi Z.M., Stepanova M., Afendy M., Fang Y., Younossi Y., Mir H., Srishord M. (2011). Changes in the prevalence of the most common causes of chronic liver diseases in the United States from 1988 to 2008. Clin. Gastroenterol. Hepatol..

[334] Younossi Z., Tacke F., Arrese M., Chander Sharma B., Mostafa I., Bugianesi E., Wai-Sun Wong V., Yilmaz Y., George J., Fan J. (2019). Global Perspectives on Nonalcoholic Fatty Liver Disease and Nonalcoholic Steatohepatitis. Hepatology.

[335] Younossi Z.M., Rinella M.E., Sanyal A.J., Harrison S.A., Brunt E.M., Goodman Z., Cohen D.E., Loomba R. (2021). From NAFLD to MAFLD: Implications of a Premature Change in Terminology. Hepatology.

[336] Molina-Molina E., Lunardi Baccetto R., Wang D.Q., de Bari O., Krawczyk M., Portincasa P. (2018). Exercising the hepatobiliary-gut axis. The impact of physical activity performance. Eur. J. Clin. Investig..

[337] Molina-Molina E., Krawczyk M., Stachowska E., Lammert F., Portincasa P. (2019). Non-Alcoholic Fatty Liver Disease in Non-Obese Individuals: Prevalence, Pathogenesis and Treatment. Clin. Res. Hepatol. Gastroenterol..

[338] Zhou J., Zhou F., Wang W., Zhang X.J., Ji Y.X., Zhang P., She Z.G., Zhu L., Cai J., Li H. (2020). Epidemiological Features of NAFLD From 1999 to 2018 in China. Hepatology.

[339] Kim D., Kim W.R. (2017). Nonobese Fatty Liver Disease. Clin. Gastroenterol. Hepatol..

[340] Yoshitaka H., Hamaguchi M., Kojima T., Fukuda T., Ohbora A., Fukui M. (2017). Nonoverweight nonalcoholic fatty liver disease and incident cardiovascular disease: A post hoc analysis of a cohort study. Medicine.

[341] Palmentieri B., de Sio I., La Mura V., Masarone M., Vecchione R., Bruno S., Torella R., Persico M. (2006). The role of bright liver echo pattern on ultrasound B-mode examination in the diagnosis of liver steatosis. Dig. Liver Dis..

[342] Adams L.A., Lymp J.F., St Sauver J., Sanderson S.O., Lindor K.D., Feldstein A., Angulo P. (2005). The natural history of nonalcoholic fatty liver disease: A population-based cohort study. Gastroenterology.

[343] Lindenmeyer C.C., McCullough A.J. (2018). The Natural History of Nonalcoholic Fatty Liver Disease-An Evolving View. Clin. Liver Dis..

[344] Rinella M.E., Sanyal A.J. (2016). Management of NAFLD: A stage-based approach. Nat. Rev. Gastroenterol. Hepatol..

[345] Loomba R., Friedman S.L., Shulman G.I. (2021). Mechanisms and disease consequences of nonalcoholic fatty liver disease. Cell.

[346] Samuel V.T., Shulman G.I. (2018). Nonalcoholic fatty liver disease as a nexus of metabolic and hepatic diseases. Cell Metab..

[347] Caussy C., Soni M., Cui J., Bettencourt R., Schork N., Chen C.H., Ikhwan M.A., Bassirian S., Cepin S., Gonzalez M.P. (2017). Nonalcoholic fatty liver disease with cirrhosis increases familial risk for advanced fibrosis. J. Clin. Investig..

[348] Stender S., Loomba R. (2020). PNPLA3 Genotype and Risk of Liver and All-Cause Mortality. Hepatology.

[349] Krawczyk M., Portincasa P., Lammert F. (2013). PNPLA3-associated steatohepatitis: Toward a gene-based classification of fatty liver disease. Semin. Liver Dis..

[350] Moschen A.R., Kaser S., Tilg H. (2013). Non-alcoholic steatohepatitis: A microbiota-driven disease. Trends Endocrinol. Metab..

[351] Loomba R., Lim J.K., Patton H., El-Serag H.B. (2020). AGA Clinical Practice Update on Screening and Surveillance for Hepatocellular Carcinoma in Patients With Nonalcoholic Fatty Liver Disease: Expert Review. Gastroenterology.

[352] Eslam M., Newsome P.N., Sarin S.K., Anstee Q.M., Targher G., Romero-Gomez M., Zelber-Sagi S., Wai-Sun Wong V., Dufour J.F., Schattenberg J.M. (2020). A new definition for metabolic dysfunction-associated fatty liver disease: An international expert consensus statement. J. Hepatol..

[353] Méndez-Sánchez N., Díaz-Orozco L., Córdova-Gallardo J. (2021). Redefinition of fatty liver disease from NAFLD to MAFLD raised disease awareness: Mexican experience. J. Hepatol..

[354] Nan Y., An J., Bao J., Chen H., Chen Y., Ding H., Dou X., Duan Z., Fan J., Gao Y. (2021). The Chinese Society of Hepatology position statement on the redefinition of fatty liver disease. J. Hepatol..

[355] Eslam M., Sarin S.K., Wong V.W., Fan J.G., Kawaguchi T., Ahn S.H., Zheng M.H., Shiha G., Yilmaz Y., Gani R. (2020). The Asian Pacific Association for the Study of the Liver clinical practice guidelines for the diagnosis and management of metabolic associated fatty liver disease. Hepatol. Int..

[356] Shiha G., Korenjak M., Eskridge W., Casanovas T., Velez-Moller P., Hogstrom S., Richardson B., Munoz C., Sigurethardottir S., Coulibaly A. (2021). Redefining fatty liver disease: An international patient perspective. Lancet. Gastroenterol. Hepatol..

[357] Shiha G., Alswat K., Al Khatry M., Sharara A.I., Ormeci N., Waked I., Benazzouz M., Al-Ali F., Hamed A.E., Hamoudi W. (2021). Nomenclature and definition of metabolic-associated fatty liver disease: A consensus from the Middle East and north Africa. Lancet. Gastroenterol. Hepatol..

[358] Tilg H., Effenberger M. (2020). From NAFLD to MAFLD: When pathophysiology succeeds. Nat. Rev. Gastroenterol. Hepatol..

[359] Di Ciaula A., Carbone F., Shanmugham H., Molina-Molina E., Bonfrate L., Ministrini S., Montecucco F., Portincasa P. (2021). Adiponectin involved in portal flow hepatic extraction of 13C-metacethin in obesity and non-alcoholic fatty liver. Eur. J. Intern. Med..

[360] Karlsen T.H., Lammert F., Thompson R.J. (2015). Genetics of liver disease: From pathophysiology to clinical practice. J. Hepatol..

[361] Albillos A., Gottardi A., Rescigno M. (2019). The gut-liver axis in liver disease: Pathophysiological basis for therapy. J. Hepatol..

[362] Kim D., Yoo E.R., Li A.A., Cholankeril G., Tighe S.P., Kim W., Harrison S.A., Ahmed A. (2019). Elevated urinary bisphenol A levels are associated with non-alcoholic fatty liver disease among adults in the United States. Liver Int..

[363] Franco M.E., Fernandez-Luna M.T., Ramirez A.J., Lavado R. (2020). Metabolomic-based assessment reveals dysregulation of lipid profiles in human liver cells exposed to environmental obesogens. Toxicol. Appl. Pharmacol..

[364] Wahlang B., Appana S., Falkner K.C., McClain C.J., Brock G., Cave M.C. (2020). Insecticide and metal exposures are associated with a surrogate biomarker for non-alcoholic fatty liver disease in the National Health and Nutrition Examination Survey 2003-2004. Environ. Sci. Pollut. Res. Int..

[365] Milosevic N., Milanovic M., Sudji J., Bosic Zivanovic D., Stojanoski S., Vukovic B., Milic N., Medic Stojanoska M. (2020). Could phthalates exposure contribute to the development of metabolic syndrome and liver disease in humans?. Environ. Sci. Pollut. Res. Int..

[366] Wang X., Yang Y., Zhu P., Wu Y., Jin Y., Yu S., Wei H., Qian M., Cao W., Xu S. (2019). Prenatal exposure to diesel exhaust PM2.5 programmed non-alcoholic fatty liver disease differently in adult male offspring of mice fed normal chow and a high-fat diet. Environ. Pollut..

[367] Chen R., Xu Y., Xu C., Shu Y., Ma S., Lu C., Mo X. (2019). Associations between mercury exposure and the risk of nonalcoholic fatty liver disease (NAFLD) in US adolescents. Environ. Sci. Pollut. Res. Int..

[368] Ding S., Yuan C., Si B., Wang M., Da S., Bai L., Wu W. (2019). Combined effects of ambient particulate matter exposure and a high-fat diet on oxidative stress and steatohepatitis in mice. PLoS ONE.

[369] Xu M.X., Ge C.X., Qin Y.T., Gu T.T., Lou D.S., Li Q., Hu L.F., Feng J., Huang P., Tan J. (2019). Prolonged PM2.5 exposure elevates risk of oxidative stress-driven nonalcoholic fatty liver disease by triggering increase of dyslipidemia. Free Radic. Biol. Med..

[370] Brown K., DeCoffe D., Molcan E., Gibson D.L. (2012). Diet-induced dysbiosis of the intestinal microbiota and the effects on immunity and disease. Nutrients.

[371] Roger L.C., Costabile A., Holland D.T., Hoyles L., McCartney A.L. (2010). Examination of faecal Bifidobacterium populations in breast- and formula-fed infants during the first 18 months of life. Microbiology.

[372] Pozo-Rubio T., Mujico J.R., Marcos A., Puertollano E., Nadal I., Sanz Y., Nova E. (2011). Immunostimulatory effect of faecal Bifidobacterium species of breast-fed and formula-fed infants in a peripheral blood mononuclear cell/Caco-2 co-culture system. Br. J. Nutr..

[373] Benno Y., Sawada K., Mitsuoka T. (1984). The intestinal microflora of infants: Composition of fecal flora in breast-fed and bottle-fed infants. Microbiol. Immunol..

[374] Jones M.L., Martoni C.J., Prakash S. (2012). Cholesterol lowering and inhibition of sterol absorption by Lactobacillus reuteri NCIMB 30242: A randomized controlled trial. Eur. J. Clin. Nutr..

[375] Gibson G.R., Probert H.M., Loo J.V., Rastall R.A., Roberfroid M.B. (2004). Dietary modulation of the human colonic microbiota: Updating the concept of prebiotics. Nutr. Res. Rev..

[376] Macfarlane G.T., Steed H., Macfarlane S. (2008). Bacterial metabolism and health-related effects of galacto-oligosaccharides and other prebiotics. J. Appl. Microbiol..

[377] Turnbaugh P.J., Ridaura V.K., Faith J.J., Rey F.E., Knight R., Gordon J.I. (2009). The effect of diet on the human gut microbiome: A metagenomic analysis in humanized gnotobiotic mice. Sci. Transl. Med..

[378] Zimmer J., Lange B., Frick J.S., Sauer H., Zimmermann K., Schwiertz A., Rusch K., Klosterhalfen S., Enck P. (2012). A vegan or vegetarian diet substantially alters the human colonic faecal microbiota. Eur. J. Clin. Nutr..

[379] Kim M.S., Hwang S.S., Park E.J., Bae J.W. (2013). Strict vegetarian diet improves the risk factors associated with metabolic diseases by modulating gut microbiota and reducing intestinal inflammation. Environ. Microbiol. Rep..

[380] De Filippo C., Cavalieri D., Di Paola M., Ramazzotti M., Poullet J.B., Massart S., Collini S., Pieraccini G., Lionetti P. (2010). Impact of diet in shaping gut microbiota revealed by a comparative study in children from Europe and rural Africa. Proc. Natl. Acad. Sci. USA.

[381] Hamilton M.K., Boudry G., Lemay D.G., Raybould H.E. (2015). Changes in intestinal barrier function and gut microbiota in high-fat diet-fed rats are dynamic and region dependent. Am. J. Physiol. Gastrointest. Liver Physiol..

[382] Kim K.A., Gu W., Lee I.A., Joh E.H., Kim D.H. (2012). High fat diet-induced gut microbiota exacerbates inflammation and obesity in mice via the TLR4 signaling pathway. PLoS ONE.

[383] Chen D., Yang Z., Chen X., Huang Y., Yin B., Guo F., Zhao H., Huang J., Wu Y., Gu R. (2015). Effect of Lactobacillus rhamnosus hsryfm 1301 on the Gut Microbiota and Lipid Metabolism in Rats Fed a High-Fat Diet. J. Microbiol. Biotechnol..

[384] Zhang C., Li S., Yang L., Huang P., Li W., Wang S., Zhao G., Zhang M., Pang X., Yan Z. (2013). Structural modulation of gut microbiota in life-long calorie-restricted mice. Nat. Commun..

[385] Hooper L.V., Littman D.R., Macpherson A.J. (2012). Interactions between the microbiota and the immune system. Science.

[386] Thaiss C.A., Zmora N., Levy M., Elinav E. (2016). The microbiome and innate immunity. Nature.

[387] Britanova L., Diefenbach A. (2017). Interplay of innate lymphoid cells and the microbiota. Immunol. Rev..

[388] Machado M.V., Cortez-Pinto H. (2016). Diet, Microbiota, Obesity, and NAFLD: A Dangerous Quartet. Int. J. Mol. Sci..

[389] Biedermann L., Zeitz J., Mwinyi J., Sutter-Minder E., Rehman A., Ott S.J., Steurer-Stey C., Frei A., Frei P., Scharl M. (2013). Smoking cessation induces profound changes in the composition of the intestinal microbiota in humans. PLoS ONE.

[390] Mutlu E.A., Gillevet P.M., Rangwala H., Sikaroodi M., Naqvi A., Engen P.A., Kwasny M., Lau C.K., Keshavarzian A. (2012). Colonic microbiome is altered in alcoholism. Am. J. Physiol. Gastrointest. Liver Physiol..

[391] Clarke S.F., Murphy E.F., Nilaweera K., Ross P.R., Shanahan F., O’Toole P.W., Cotter P.D. (2012). The gut microbiota and its relationship to diet and obesity: New insights. Gut Microbes.

[392] Henao-Mejia J., Elinav E., Jin C., Hao L., Mehal W.Z., Strowig T., Thaiss C.A., Kau A.L., Eisenbarth S.C., Jurczak M.J. (2012). Inflammasome-mediated dysbiosis regulates progression of NAFLD and obesity. Nature.

[393] Turnbaugh P.J., Hamady M., Yatsunenko T., Cantarel B.L., Duncan A., Ley R.E., Sogin M.L., Jones W.J., Roe B.A., Affourtit J.P. (2009). A core gut microbiome in obese and lean twins. Nature.

[394] Vrieze A., Van Nood E., Holleman F., Salojarvi J., Kootte R.S., Bartelsman J.F., Dallinga-Thie G.M., Ackermans M.T., Serlie M.J., Oozeer R. (2012). Transfer of intestinal microbiota from lean donors increases insulin sensitivity in individuals with metabolic syndrome. Gastroenterology.

[395] Moreira G.V., Azevedo F.F., Ribeiro L.M., Santos A., Guadagnini D., Gama P., Liberti E.A., Saad M., Carvalho C. (2018). Liraglutide modulates gut microbiota and reduces NAFLD in obese mice. J. Nutr. Biochem..

[396] Brandt A., Hernandez-Arriaga A., Kehm R., Sanchez V., Jin C.J., Nier A., Baumann A., Camarinha-Silva A., Bergheim I. (2019). Metformin attenuates the onset of non-alcoholic fatty liver disease and affects intestinal microbiota and barrier in small intestine. Sci. Rep..

[397] Feng W., Wang H., Zhang P., Gao C., Tao J., Ge Z., Zhu D., Bi Y. (2017). Modulation of gut microbiota contributes to curcumin-mediated attenuation of hepatic steatosis in rats. Biochim. Biophys. Acta Gen. Subj..

[398] Gao B., Chi L., Mahbub R., Bian X., Tu P., Ru H., Lu K. (2017). Multi-Omics Reveals that Lead Exposure Disturbs Gut Microbiome Development, Key Metabolites, and Metabolic Pathways. Chem. Res. Toxicol..

[399] Gao B., Bian X., Mahbub R., Lu K. (2017). Sex-Specific Effects of Organophosphate Diazinon on the Gut Microbiome and Its Metabolic Functions. Environ. Health Perspect..

[400] Joly C., Gay-Queheillard J., Leke A., Chardon K., Delanaud S., Bach V., Khorsi-Cauet H. (2013). Impact of chronic exposure to low doses of chlorpyrifos on the intestinal microbiota in the Simulator of the Human Intestinal Microbial Ecosystem (SHIME) and in the rat. Environ. Sci. Pollut. Res. Int..

[401] Joly Condette C., Bach V., Mayeur C., Gay-Queheillard J., Khorsi-Cauet H. (2015). Chlorpyrifos Exposure During Perinatal Period Affects Intestinal Microbiota Associated With Delay of Maturation of Digestive Tract in Rats. J. Pediatric Gastroenterol. Nutr..

[402] Wahlang B., Jin J., Beier J.I., Hardesty J.E., Daly E.F., Schnegelberger R.D., Falkner K.C., Prough R.A., Kirpich I.A., Cave M.C. (2019). Mechanisms of Environmental Contributions to Fatty Liver Disease. Curr. Environ. Health Rep..

[403] Schnabl B., Brenner D.A. (2014). Interactions between the intestinal microbiome and liver diseases. Gastroenterology.

[404] Bischoff S.C., Barbara G., Buurman W., Ockhuizen T., Schulzke J.D., Serino M., Tilg H., Watson A., Wells J.M. (2014). Intestinal permeability--a new target for disease prevention and therapy. BMC Gastroenterol..

[405] Kirpich I.A., Marsano L.S., McClain C.J. (2015). Gut-liver axis, nutrition, and non-alcoholic fatty liver disease. Clin. Biochem..

[406] Schroeder B.O., Birchenough G.M.H., Stahlman M., Arike L., Johansson M.E.V., Hansson G.C., Backhed F. (2018). Bifidobacteria or Fiber Protects against Diet-Induced Microbiota-Mediated Colonic Mucus Deterioration. Cell Host Microbe.

[407] Luck H., Tsai S., Chung J., Clemente-Casares X., Ghazarian M., Revelo X.S., Lei H., Luk C.T., Shi S.Y., Surendra A. (2015). Regulation of obesity-related insulin resistance with gut anti-inflammatory agents. Cell Metab..

[408] Serino M., Luche E., Gres S., Baylac A., Berge M., Cenac C., Waget A., Klopp P., Iacovoni J., Klopp C. (2012). Metabolic adaptation to a high-fat diet is associated with a change in the gut microbiota. Gut.

[409] Spruss A., Kanuri G., Wagnerberger S., Haub S., Bischoff S.C., Bergheim I. (2009). Toll-like receptor 4 is involved in the development of fructose-induced hepatic steatosis in mice. Hepatology.

[410] Lambertz J., Weiskirchen S., Landert S., Weiskirchen R. (2017). Fructose: A Dietary Sugar in Crosstalk with Microbiota Contributing to the Development and Progression of Non-Alcoholic Liver Disease. Front. Immunol..

[411] Ray K. (2015). NAFLD. Leaky guts: Intestinal permeability and NASH. Nat. Rev. Gastroenterol. Hepatol..

[412] Miele L., Marrone G., Lauritano C., Cefalo C., Gasbarrini A., Day C., Grieco A. (2013). Gut-liver axis and microbiota in NAFLD: Insight pathophysiology for novel therapeutic target. Curr. Pharm. Des..

[413] Cani P.D., Possemiers S., Van de Wiele T., Guiot Y., Everard A., Rottier O., Geurts L., Naslain D., Neyrinck A., Lambert D.M. (2009). Changes in gut microbiota control inflammation in obese mice through a mechanism involving GLP-2-driven improvement of gut permeability. Gut.

[414] Ding S., Chi M.M., Scull B.P., Rigby R., Schwerbrock N.M., Magness S., Jobin C., Lund P.K. (2010). High-fat diet: Bacteria interactions promote intestinal inflammation which precedes and correlates with obesity and insulin resistance in mouse. PLoS ONE.

[415] Kavanagh K., Wylie A.T., Tucker K.L., Hamp T.J., Gharaibeh R.Z., Fodor A.A., Cullen J.M. (2013). Dietary fructose induces endotoxemia and hepatic injury in calorically controlled primates. Am. J. Clin. Nutr..

[416] Rahman K., Desai C., Iyer S.S., Thorn N.E., Kumar P., Liu Y., Smith T., Neish A.S., Li H., Tan S. (2016). Loss of Junctional Adhesion Molecule A Promotes Severe Steatohepatitis in Mice on a Diet High in Saturated Fat, Fructose, and Cholesterol. Gastroenterology.

[417] Bluemel S., Wang L., Martino C., Lee S., Wang Y., Williams B., Horvath A., Stadlbauer V., Zengler K., Schnabl B. (2018). The Role of Intestinal C-type Regenerating Islet Derived-3 Lectins for Nonalcoholic Steatohepatitis. Hepatol. Commun..

[418] Li Z., Yang S., Lin H., Huang J., Watkins P.A., Moser A.B., Desimone C., Song X.Y., Diehl A.M. (2003). Probiotics and antibodies to TNF inhibit inflammatory activity and improve nonalcoholic fatty liver disease. Hepatology.

[419] Jin R., Willment A., Patel S.S., Sun X., Song M., Mannery Y.O., Kosters A., McClain C.J., Vos M.B. (2014). Fructose induced endotoxemia in pediatric nonalcoholic Fatty liver disease. Int. J. Hepatol..

[420] Bifulco M. (2015). Mediterranean diet: The missing link between gut microbiota and inflammatory diseases. Eur. J. Clin. Nutr..

[421] Biolato M., Manca F., Marrone G., Cefalo C., Racco S., Miggiano G.A., Valenza V., Gasbarrini A., Miele L., Grieco A. (2019). Intestinal permeability after Mediterranean diet and low-fat diet in non-alcoholic fatty liver disease. World J. Gastroenterol..

[422] Enomoto N., Yamashina S., Kono H., Schemmer P., Rivera C.A., Enomoto A., Nishiura T., Nishimura T., Brenner D.A., Thurman R.G. (1999). Development of a new, simple rat model of early alcohol-induced liver injury based on sensitization of Kupffer cells. Hepatology.

[423] Pappo I., Bercovier H., Berry E.M., Haviv Y., Gallily R., Freund H.R. (1992). Polymyxin B reduces total parenteral nutrition-associated hepatic steatosis by its antibacterial activity and by blocking deleterious effects of lipopolysaccharide. JPEN J. Parenter Enter. Nutr..

[424] Pappo I., Becovier H., Berry E.M., Freund H.R. (1991). Polymyxin B reduces cecal flora, TNF production and hepatic steatosis during total parenteral nutrition in the rat. J. Surg. Res..

[425] Drenick E.J., Fisler J., Johnson D. (1982). Hepatic steatosis after intestinal bypass--prevention and reversal by metronidazole, irrespective of protein-calorie malnutrition. Gastroenterology.

[426] Alisi A., Bedogni G., Baviera G., Giorgio V., Porro E., Paris C., Giammaria P., Reali L., Anania F., Nobili V. (2014). Randomised clinical trial: The beneficial effects of VSL#3 in obese children with non-alcoholic steatohepatitis. Aliment. Pharmacol. Ther..

[427] Backhed F., Ding H., Wang T., Hooper L.V., Koh G.Y., Nagy A., Semenkovich C.F., Gordon J.I. (2004). The gut microbiota as an environmental factor that regulates fat storage. Proc. Natl. Acad. Sci. USA.

[428] Thuny F., Richet H., Casalta J.P., Angelakis E., Habib G., Raoult D. (2010). Vancomycin treatment of infective endocarditis is linked with recently acquired obesity. PLoS ONE.

[429] Saari A., Virta L.J., Sankilampi U., Dunkel L., Saxen H. (2015). Antibiotic exposure in infancy and risk of being overweight in the first 24 months of life. Pediatrics.

[430] Cox A.J., West N.P., Cripps A.W. (2015). Obesity, inflammation, and the gut microbiota. Lancet Diabetes Endocrinol.

[431] Cox L.M., Yamanishi S., Sohn J., Alekseyenko A.V., Leung J.M., Cho I., Kim S.G., Li H., Gao Z., Mahana D. (2014). Altering the intestinal microbiota during a critical developmental window has lasting metabolic consequences. Cell.

[432] Ley R.E., Backhed F., Turnbaugh P., Lozupone C.A., Knight R.D., Gordon J.I. (2005). Obesity alters gut microbial ecology. Proc. Natl. Acad. Sci. USA.

[433] Turnbaugh P.J., Ley R.E., Mahowald M.A., Magrini V., Mardis E.R., Gordon J.I. (2006). An obesity-associated gut microbiome with increased capacity for energy harvest. Nature.

[434] Cani P.D., Osto M., Geurts L., Everard A. (2012). Involvement of gut microbiota in the development of low-grade inflammation and type 2 diabetes associated with obesity. Gut Microbes.

[435] Cox L.M., Blaser M.J. (2013). Pathways in microbe-induced obesity. Cell Metab..

[436] Zhang H., DiBaise J.K., Zuccolo A., Kudrna D., Braidotti M., Yu Y., Parameswaran P., Crowell M.D., Wing R., Rittmann B.E. (2009). Human gut microbiota in obesity and after gastric bypass. Proc. Natl. Acad. Sci. USA.

[437] Liou A.P., Paziuk M., Luevano J.-M., Machineni S., Turnbaugh P.J., Kaplan L.M. (2013). Conserved shifts in the gut microbiota due to gastric bypass reduce host weight and adiposity. Sci. Transl. Med..

[438] Gao Z., Yin J., Zhang J., Ward R.E., Martin R.J., Lefevre M., Cefalu W.T., Ye J. (2009). Butyrate improves insulin sensitivity and increases energy expenditure in mice. Diabetes.

[439] Petersen C., Bell R., Klag K.A., Lee S.-H., Soto R., Ghazaryan A., Buhrke K., Ekiz H.A., Ost K.S., Boudina S. (2019). T cell–mediated regulation of the microbiota protects against obesity. Science.

[440] Luck H., Khan S., Kim J.H., Copeland J.K., Revelo X.S., Tsai S., Chakraborty M., Cheng K., Chan Y.T., Nøhr M.K. (2019). Gut-associated IgA+ immune cells regulate obesity-related insulin resistance. Nat. Commun..

[441] Tilg H., Zmora N., Adolph T.E., Elinav E. (2020). The intestinal microbiota fuelling metabolic inflammation. Nat. Rev. Immunol..

[442] Murphy E.F., Cotter P.D., Healy S., Marques T.M., O’Sullivan O., Fouhy F., Clarke S.F., O’Toole P.W., Quigley E.M., Stanton C. (2010). Composition and energy harvesting capacity of the gut microbiota: Relationship to diet, obesity and time in mouse models. Gut.

[443] Schwiertz A., Taras D., Schafer K., Beijer S., Bos N.A., Donus C., Hardt P.D. (2010). Microbiota and SCFA in lean and overweight healthy subjects. Obesity.

[444] Fernandes J., Su W., Rahat-Rozenbloom S., Wolever T.M., Comelli E.M. (2014). Adiposity, gut microbiota and faecal short chain fatty acids are linked in adult humans. Nutr. Diabetes.

[445] Lin H.V., Frassetto A., Kowalik E.J., Nawrocki A.R., Lu M.M., Kosinski J.R., Hubert J.A., Szeto D., Yao X., Forrest G. (2012). Butyrate and propionate protect against diet-induced obesity and regulate gut hormones via free fatty acid receptor 3-independent mechanisms. PLoS ONE.

[446] Yamashita H., Fujisawa K., Ito E., Idei S., Kawaguchi N., Kimoto M., Hiemori M., Tsuji H. (2007). Improvement of obesity and glucose tolerance by acetate in Type 2 diabetic Otsuka Long-Evans Tokushima Fatty (OLETF) rats. Biosci. Biotechnol. Biochem..

[447] Den Besten G., van Eunen K., Groen A.K., Venema K., Reijngoud D.-J., Bakker B.M. (2013). The role of short-chain fatty acids in the interplay between diet, gut microbiota, and host energy metabolism. J. Lipid Res..

[448] Kimura I., Inoue D., Hirano K., Tsujimoto G. (2014). The SCFA Receptor GPR43 and Energy Metabolism. Front. Endocrinol..

[449] Kasubuchi M., Hasegawa S., Hiramatsu T., Ichimura A., Kimura I. (2015). Dietary gut microbial metabolites, short-chain fatty acids, and host metabolic regulation. Nutrients.

[450] Bjursell M., Admyre T., Goransson M., Marley A.E., Smith D.M., Oscarsson J., Bohlooly Y.M. (2011). Improved glucose control and reduced body fat mass in free fatty acid receptor 2-deficient mice fed a high-fat diet. Am. J. Physiol. Endocrinol. Metab..

[451] Kimura I., Ozawa K., Inoue D., Imamura T., Kimura K., Maeda T., Terasawa K., Kashihara D., Hirano K., Tani T. (2013). The gut microbiota suppresses insulin-mediated fat accumulation via the short-chain fatty acid receptor GPR43. Nat. Commun..

[452] Chambers E.S., Morrison D.J., Frost G. (2015). Control of appetite and energy intake by SCFA: What are the potential underlying mechanisms?. Proc. Nutr. Soc..

[453] Kaji I., Karaki S., Kuwahara A. (2014). Short-chain fatty acid receptor and its contribution to glucagon-like peptide-1 release. Digestion.

[454] Conterno L., Fava F., Viola R., Tuohy K.M. (2011). Obesity and the gut microbiota: Does up-regulating colonic fermentation protect against obesity and metabolic disease?. Genes Nutr..

[455] Creely S.J., McTernan P.G., Kusminski C.M., Fisher f M., Da Silva N.F., Khanolkar M., Evans M., Harte A.L., Kumar S. (2007). Lipopolysaccharide activates an innate immune system response in human adipose tissue in obesity and type 2 diabetes. Am. J. Physiol. Endocrinol. Metab..

[456] Karagiannides I., Pothoulakis C. (2007). Obesity, innate immunity and gut inflammation. Curr. Opin. Gastroenterol..

[457] Kim S.J., Choi Y., Choi Y.H., Park T. (2012). Obesity activates toll-like receptor-mediated proinflammatory signaling cascades in the adipose tissue of mice. J. Nutr. Biochem..

[458] Ye D., Li F.Y., Lam K.S., Li H., Jia W., Wang Y., Man K., Lo C.M., Li X., Xu A. (2012). Toll-like receptor-4 mediates obesity-induced non-alcoholic steatohepatitis through activation of X-box binding protein-1 in mice. Gut.

[459] Rodes L., Khan A., Paul A., Coussa-Charley M., Marinescu D., Tomaro-Duchesneau C., Shao W., Kahouli I., Prakash S. (2013). Effect of probiotics Lactobacillus and Bifidobacterium on gut-derived lipopolysaccharides and inflammatory cytokines: An in vitro study using a human colonic microbiota model. J. Microbiol. Biotechnol..

[460] Martinez-Lopez M., Iborra S., Conde-Garrosa R., Mastrangelo A., Danne C., Mann E.R., Reid D.M., Gaboriau-Routhiau V., Chaparro M., Lorenzo M.P. (2019). Microbiota Sensing by Mincle-Syk Axis in Dendritic Cells Regulates Interleukin-17 and -22 Production and Promotes Intestinal Barrier Integrity. Immunity.

[461] Wang X., Ota N., Manzanillo P., Kates L., Zavala-Solorio J., Eidenschenk C., Zhang J., Lesch J., Lee W.P., Ross J. (2014). Interleukin-22 alleviates metabolic disorders and restores mucosal immunity in diabetes. Nature.

[462] Yusta B., Baggio L.L., Koehler J., Holland D., Cao X., Pinnell L.J., Johnson-Henry K.C., Yeung W., Surette M.G., Bang K.A. (2015). GLP-1R agonists modulate enteric immune responses through the intestinal intraepithelial lymphocyte GLP-1R. Diabetes.

[463] Tsai S., Winer S., Winer D.A. (2019). Gut T cells feast on GLP-1 to modulate cardiometabolic disease. Cell Metab..

[464] Cani P.D. (2018). Human gut microbiome: Hopes, threats and promises. Gut.

[465] Tolhurst G., Heffron H., Lam Y.S., Parker H.E., Habib A.M., Diakogiannaki E., Cameron J., Grosse J., Reimann F., Gribble F.M. (2012). Short-chain fatty acids stimulate glucagon-like peptide-1 secretion via the G-protein-coupled receptor FFAR2. Diabetes.

[466] Nøhr M.K., Pedersen M.H., Gille A., Egerod K.L., Engelstoft M.S., Husted A.S., Sichlau R.M., Grunddal K.V., Seier Poulsen S., Han S. (2013). GPR41/FFAR3 and GPR43/FFAR2 as cosensors for short-chain fatty acids in enteroendocrine cells vs FFAR3 in enteric neurons and FFAR2 in enteric leukocytes. Endocrinology.

[467] Tai N., Wong F.S., Wen L. (2015). The role of gut microbiota in the development of type 1, type 2 diabetes mellitus and obesity. Rev. Endocr. Metab. Disord..

[468] Cani P.D., Delzenne N.M. (2009). The role of the gut microbiota in energy metabolism and metabolic disease. Curr. Pharm. Des..

[469] Tanti J.F., Ceppo F., Jager J., Berthou F. (2012). Implication of inflammatory signaling pathways in obesity-induced insulin resistance. Front. Endocrinol..

[470] Dali-Youcef N., Mecili M., Ricci R., Andres E. (2013). Metabolic inflammation: Connecting obesity and insulin resistance. Ann. Med..

[471] Amyot J., Semache M., Ferdaoussi M., Fontes G., Poitout V. (2012). Lipopolysaccharides impair insulin gene expression in isolated islets of Langerhans via Toll-Like Receptor-4 and NF-kappaB signalling. PLoS ONE.

[472] Grabherr F., Grander C., Effenberger M., Adolph T.E., Tilg H. (2019). Gut Dysfunction and Non-alcoholic Fatty Liver Disease. Front. Endocrinol..

[473] Moretti C.H., Schiffer T.A., Li X., Weitzberg E., Carlstrom M., Lundberg J.O. (2021). Germ-free mice are not protected against diet-induced obesity and metabolic dysfunction. Acta Physiol..

[474] Loomba R., Seguritan V., Li W., Long T., Klitgord N., Bhatt A., Dulai P.S., Caussy C., Bettencourt R., Highlander S.K. (2017). Gut Microbiome-Based Metagenomic Signature for Non-invasive Detection of Advanced Fibrosis in Human Nonalcoholic Fatty Liver Disease. Cell Metab..

[475] Wiest R., Albillos A., Trauner M., Bajaj J.S., Jalan R. (2017). Targeting the gut-liver axis in liver disease. J. Hepatol..

[476] Pihlajamaki J., Kuulasmaa T., Kaminska D., Simonen M., Karja V., Gronlund S., Kakela P., Paakkonen M., Kainulainen S., Punnonen K. (2012). Serum interleukin 1 receptor antagonist as an independent marker of non-alcoholic steatohepatitis in humans. J. Hepatol..

[477] Zimmermann E., Anty R., Tordjman J., Verrijken A., Gual P., Tran A., Iannelli A., Gugenheim J., Bedossa P., Francque S. (2011). C-reactive protein levels in relation to various features of non-alcoholic fatty liver disease among obese patients. J. Hepatol..

[478] Chiang C.H., Huang C.C., Chan W.L., Chen J.W., Leu H.B. (2010). The severity of non-alcoholic fatty liver disease correlates with high sensitivity C-reactive protein value and is independently associated with increased cardiovascular risk in healthy population. Clin. Biochem..

[479] Haukeland J.W., Damas J.K., Konopski Z., Loberg E.M., Haaland T., Goverud I., Torjesen P.A., Birkeland K., Bjoro K., Aukrust P. (2006). Systemic inflammation in nonalcoholic fatty liver disease is characterized by elevated levels of CCL2. J. Hepatol..

[480] Kubes P., Mehal W.Z. (2012). Sterile inflammation in the liver. Gastroenterology.

[481] Netea M.G., Balkwill F., Chonchol M., Cominelli F., Donath M.Y., Giamarellos-Bourboulis E.J., Golenbock D., Gresnigt M.S., Heneka M.T., Hoffman H.M. (2017). A guiding map for inflammation. Nat. Immunol..

[482] Angulo P., Kleiner D.E., Dam-Larsen S., Adams L.A., Bjornsson E.S., Charatcharoenwitthaya P., Mills P.R., Keach J.C., Lafferty H.D., Stahler A. (2015). Liver Fibrosis, but No Other Histologic Features, Is Associated With Long-term Outcomes of Patients With Nonalcoholic Fatty Liver Disease. Gastroenterology.

[483] Angulo P., Machado M.V., Diehl A.M. (2015). Fibrosis in nonalcoholic Fatty liver disease: Mechanisms and clinical implications. Semin. Liver Dis..

[484] Saltiel A.R., Olefsky J.M. (2017). Inflammatory mechanisms linking obesity and metabolic disease. J. Clin. Investig..

[485] Moschen A.R., Molnar C., Enrich B., Geiger S., Ebenbichler C.F., Tilg H. (2011). Adipose and liver expression of interleukin (IL)-1 family members in morbid obesity and effects of weight loss. Mol. Med..

[486] Ballak D.B., van Diepen J.A., Moschen A.R., Jansen H.J., Hijmans A., Groenhof G.J., Leenders F., Bufler P., Boekschoten M.V., Muller M. (2014). IL-37 protects against obesity-induced inflammation and insulin resistance. Nat. Commun..

[487] Ley R.E., Turnbaugh P.J., Klein S., Gordon J.I. (2006). Microbial ecology: Human gut microbes associated with obesity. Nature.

[488] Molina-Molina E., Shanmugam H., Di Ciaula A., Grattagliano I., Di Palo D.M., Palmieri V.O., Portincasa P. (2021). ((13)C)-Methacetin breath test provides evidence of subclinical liver dysfunction linked to fat storage but not lifestyle. JHEP Rep..

[489] Serino M., Luche E., Chabo C., Amar J., Burcelin R. (2009). Intestinal microflora and metabolic diseases. Diabetes Metab..

[490] Boursier J., Mueller O., Barret M., Machado M., Fizanne L., Araujo-Perez F., Guy C.D., Seed P.C., Rawls J.F., David L.A. (2016). The severity of nonalcoholic fatty liver disease is associated with gut dysbiosis and shift in the metabolic function of the gut microbiota. Hepatology.

[491] Le Roy T., Llopis M., Lepage P., Bruneau A., Rabot S., Bevilacqua C., Martin P., Philippe C., Walker F., Bado A. (2013). Intestinal microbiota determines development of non-alcoholic fatty liver disease in mice. Gut.

[492] Janssen A.W.F., Houben T., Katiraei S., Dijk W., Boutens L., van der Bolt N., Wang Z., Brown J.M., Hazen S.L., Mandard S. (2017). Modulation of the gut microbiota impacts nonalcoholic fatty liver disease: A potential role for bile acids. J. Lipid Res..

[493] Garcia-Lezana T., Raurell I., Bravo M., Torres-Arauz M., Salcedo M.T., Santiago A., Schoenenberger A., Manichanh C., Genesca J., Martell M. (2018). Restoration of a healthy intestinal microbiota normalizes portal hypertension in a rat model of nonalcoholic steatohepatitis. Hepatology.

[494] Soderborg T.K., Clark S.E., Mulligan C.E., Janssen R.C., Babcock L., Ir D., Young B., Krebs N., Lemas D.J., Johnson L.K. (2018). The gut microbiota in infants of obese mothers increases inflammation and susceptibility to NAFLD. Nat. Commun..

[495] Wigg A.J., Roberts-Thomson I.C., Dymock R.B., McCarthy P.J., Grose R.H., Cummins A.G. (2001). The role of small intestinal bacterial overgrowth, intestinal permeability, endotoxaemia, and tumour necrosis factor alpha in the pathogenesis of non-alcoholic steatohepatitis. Gut.

[496] Luther J., Garber J.J., Khalili H., Dave M., Bale S.S., Jindal R., Motola D.L., Luther S., Bohr S., Jeoung S.W. (2015). Hepatic Injury in Nonalcoholic Steatohepatitis Contributes to Altered Intestinal Permeability. Cell Mol. Gastroenterol. Hepatol..

[497] Sung Y.K., Gwak G.Y., Choi M.S., Koh K.C., Paik S.W., Yoo B.C., Lee J.H. (2012). A case of nonalcoholic steatohepatitis and small intestinal bacterial overgrowth with peripheral edema caused by intestinal bypass surgery and relieved by repair. Gut Liver.

[498] Shanab A.A., Scully P., Crosbie O., Buckley M., O’Mahony L., Shanahan F., Gazareen S., Murphy E., Quigley E.M. (2011). Small intestinal bacterial overgrowth in nonalcoholic steatohepatitis: Association with toll-like receptor 4 expression and plasma levels of interleukin 8. Dig. Dis. Sci..

[499] Zhu L., Baker S.S., Gill C., Liu W., Alkhouri R., Baker R.D., Gill S.R. (2013). Characterization of gut microbiomes in nonalcoholic steatohepatitis (NASH) patients: A connection between endogenous alcohol and NASH. Hepatology.

[500] Mouzaki M., Comelli E.M., Arendt B.M., Bonengel J., Fung S.K., Fischer S.E., McGilvray I.D., Allard J.P. (2013). Intestinal microbiota in patients with nonalcoholic fatty liver disease. Hepatology.

[501] Da Silva H.E., Teterina A., Comelli E.M., Taibi A., Arendt B.M., Fischer S.E., Lou W., Allard J.P. (2018). Nonalcoholic fatty liver disease is associated with dysbiosis independent of body mass index and insulin resistance. Sci. Rep..

[502] Del Chierico F., Nobili V., Vernocchi P., Russo A., De Stefanis C., Gnani D., Furlanello C., Zandona A., Paci P., Capuani G. (2017). Gut microbiota profiling of pediatric nonalcoholic fatty liver disease and obese patients unveiled by an integrated meta-omics-based approach. Hepatology.

[503] Schierwagen R., Alvarez-Silva C., Madsen M.S.A., Kolbe C.C., Meyer C., Thomas D., Uschner F.E., Magdaleno F., Jansen C., Pohlmann A. (2019). Circulating microbiome in blood of different circulatory compartments. Gut.

[504] Lelouvier B., Servant F., Paisse S., Brunet A.C., Benyahya S., Serino M., Valle C., Ortiz M.R., Puig J., Courtney M. (2016). Changes in blood microbiota profiles associated with liver fibrosis in obese patients: A pilot analysis. Hepatology.

[505] Caussy C., Tripathi A., Humphrey G., Bassirian S., Singh S., Faulkner C., Bettencourt R., Rizo E., Richards L., Xu Z.Z. (2019). A gut microbiome signature for cirrhosis due to nonalcoholic fatty liver disease. Nat. Commun..

[506] Ahn S.B., Jun D.W., Kang B.K., Lim J.H., Lim S., Chung M.J. (2019). Randomized, Double-blind, Placebo-controlled Study of a Multispecies Probiotic Mixture in Nonalcoholic Fatty Liver Disease. Sci. Rep..

[507] Chen H., Nwe P.K., Yang Y., Rosen C.E., Bielecka A.A., Kuchroo M., Cline G.W., Kruse A.C., Ring A.M., Crawford J.M. (2019). A Forward Chemical Genetic Screen Reveals Gut Microbiota Metabolites That Modulate Host Physiology. Cell.

[508] Wacker D., Stevens R.C., Roth B.L. (2017). How Ligands Illuminate GPCR Molecular Pharmacology. Cell.

[509] Cohen L.J., Esterhazy D., Kim S.H., Lemetre C., Aguilar R.R., Gordon E.A., Pickard A.J., Cross J.R., Emiliano A.B., Han S.M. (2017). Commensal bacteria make GPCR ligands that mimic human signalling molecules. Nature.

[510] Lin R.S., Lee F.Y., Lee S.D., Tsai Y.T., Lin H.C., Lu R.H., Hsu W.C., Huang C.C., Wang S.S., Lo K.J. (1995). Endotoxemia in patients with chronic liver diseases: Relationship to severity of liver diseases, presence of esophageal varices, and hyperdynamic circulation. J. Hepatol..

[511] Garcia-Tsao G., Lee F.Y., Barden G.E., Cartun R., West A.B. (1995). Bacterial translocation to mesenteric lymph nodes is increased in cirrhotic rats with ascites. Gastroenterology.

[512] Cirera I., Bauer T.M., Navasa M., Vila J., Grande L., Taura P., Fuster J., Garcia-Valdecasas J.C., Lacy A., Suarez M.J. (2001). Bacterial translocation of enteric organisms in patients with cirrhosis. J. Hepatol..

[513] Bellot P., Garcia-Pagan J.C., Frances R., Abraldes J.G., Navasa M., Perez-Mateo M., Such J., Bosch J. (2010). Bacterial DNA translocation is associated with systemic circulatory abnormalities and intrahepatic endothelial dysfunction in patients with cirrhosis. Hepatology.

[514] Wenfeng Z., Yakun W., Di M., Jianping G., Chuanxin W., Chun H. (2014). Kupffer cells: Increasingly significant role in nonalcoholic fatty liver disease. Ann. Hepatol..

[515] Heymann F., Tacke F. (2016). Immunology in the liver--from homeostasis to disease. Nat. Rev. Gastroenterol. Hepatol..

[516] Duffield J.S., Forbes S.J., Constandinou C.M., Clay S., Partolina M., Vuthoori S., Wu S., Lang R., Iredale J.P. (2005). Selective depletion of macrophages reveals distinct, opposing roles during liver injury and repair. J. Clin. Investig..

[517] Seki E., De Minicis S., Osterreicher C.H., Kluwe J., Osawa Y., Brenner D.A., Schwabe R.F. (2007). TLR4 enhances TGF-beta signaling and hepatic fibrosis. Nat. Med..

[518] Ramadori G., Moriconi F., Malik I., Dudas J. (2008). Physiology and pathophysiology of liver inflammation, damage and repair. J. Physiol. Pharm..

[519] Kudo H., Takahara T., Yata Y., Kawai K., Zhang W., Sugiyama T. (2009). Lipopolysaccharide triggered TNF-alpha-induced hepatocyte apoptosis in a murine non-alcoholic steatohepatitis model. J. Hepatol..

[520] Brenner C., Galluzzi L., Kepp O., Kroemer G. (2013). Decoding cell death signals in liver inflammation. J. Hepatol..

[521] Tilg H., Moschen A.R., Szabo G. (2016). Interleukin-1 and inflammasomes in alcoholic liver disease/acute alcoholic hepatitis and nonalcoholic fatty liver disease/nonalcoholic steatohepatitis. Hepatology.

[522] Seki E., Schnabl B. (2012). Role of innate immunity and the microbiota in liver fibrosis: Crosstalk between the liver and gut. J. Physiol..

[523] Wree A., Broderick L., Canbay A., Hoffman H.M., Feldstein A.E. (2013). From NAFLD to NASH to cirrhosis-new insights into disease mechanisms. Nat. Rev. Gastroenterol. Hepatol..

[524] Tsuchida T., Friedman S.L. (2017). Mechanisms of hepatic stellate cell activation. Nat. Rev. Gastroenterol. Hepatol..

[525] Roderfeld M. (2018). Matrix metalloproteinase functions in hepatic injury and fibrosis. Matrix Biol. J. Int. Soc. Matrix Biol..

[526] Benyon R.C., Arthur M.J. (2001). Extracellular matrix degradation and the role of hepatic stellate cells. Semin. Liver Dis..

[527] Schuppan D., Ruehl M., Somasundaram R., Hahn E.G. (2001). Matrix as a modulator of hepatic fibrogenesis. Semin. Liver Dis..

[528] Knittel T., Mehde M., Kobold D., Saile B., Dinter C., Ramadori G. (1999). Expression patterns of matrix metalloproteinases and their inhibitors in parenchymal and non-parenchymal cells of rat liver: Regulation by TNF-alpha and TGF-beta1. J. Hepatol..

[529] Miele L., Forgione A., La Torre G., Vero V., Cefalo C., Racco S., Vellone V.G., Vecchio F.M., Gasbarrini G., Rapaccini G.L. (2009). Serum levels of hyaluronic acid and tissue metalloproteinase inhibitor-1 combined with age predict the presence of nonalcoholic steatohepatitis in a pilot cohort of subjects with nonalcoholic fatty liver disease. Transl. Res. J. Lab. Clin. Med..

[530] Cichoz-Lach H., Michalak A. (2014). Oxidative stress as a crucial factor in liver diseases. World J. Gastroenterol..

[531] Luangmonkong T., Suriguga S., Mutsaers H.A.M., Groothuis G.M.M., Olinga P., Boersema M. (2018). Targeting Oxidative Stress for the Treatment of Liver Fibrosis. Rev. Physiol. Biochem. Pharmacol..

[532] Li S., Tan H.Y., Wang N., Zhang Z.J., Lao L., Wong C.W., Feng Y. (2015). The Role of Oxidative Stress and Antioxidants in Liver Diseases. Int. J. Mol. Sci..

[533] Grattagliano I., Caraceni P., Calamita G., Ferri D., Gargano I., Palasciano G., Portincasa P. (2008). Severe liver steatosis correlates with nitrosative and oxidative stress in rats. Eur. J. Clin. Investig..

[534] Gil-Cardoso K., Gines I., Pinent M., Ardevol A., Terra X., Blay M. (2017). A cafeteria diet triggers intestinal inflammation and oxidative stress in obese rats. Br. J. Nutr..

[535] Keshavarzian A., Farhadi A., Forsyth C.B., Rangan J., Jakate S., Shaikh M., Banan A., Fields J.Z. (2009). Evidence that chronic alcohol exposure promotes intestinal oxidative stress, intestinal hyperpermeability and endotoxemia prior to development of alcoholic steatohepatitis in rats. J. Hepatol..

[536] Van Ampting M.T., Schonewille A.J., Vink C., Brummer R.J., van der Meer R., Bovee-Oudenhoven I.M. (2009). Intestinal barrier function in response to abundant or depleted mucosal glutathione in Salmonella-infected rats. BMC Physiol..

[537] Novak E.A., Mollen K.P. (2015). Mitochondrial dysfunction in inflammatory bowel disease. Front. Cell Dev. Biol..

[538] Utzeri E., Usai P. (2017). Role of non-steroidal anti-inflammatory drugs on intestinal permeability and nonalcoholic fatty liver disease. World J. Gastroenterol..

[539] Ramachandran A., Prabhu R., Thomas S., Reddy J.B., Pulimood A., Balasubramanian K.A. (2002). Intestinal mucosal alterations in experimental cirrhosis in the rat: Role of oxygen free radicals. Hepatology.

[540] Liang S., Kisseleva T., Brenner D.A. (2016). The Role of NADPH Oxidases (NOXs) in Liver Fibrosis and the Activation of Myofibroblasts. Front. Physiol..

[541] Nieto N. (2006). Oxidative-stress and IL-6 mediate the fibrogenic effects of [corrected] Kupffer cells on stellate cells. Hepatology.

[542] Krause P., Morris V., Greenbaum J.A., Park Y., Bjoerheden U., Mikulski Z., Muffley T., Shui J.W., Kim G., Cheroutre H. (2015). IL-10-producing intestinal macrophages prevent excessive antibacterial innate immunity by limiting IL-23 synthesis. Nat. Commun..

[543] Gomez-Hurtado I., Moratalla A., Moya-Perez A., Peiro G., Zapater P., Gonzalez-Navajas J.M., Gimenez P., Such J., Sanz Y., Frances R. (2014). Role of interleukin 10 in norfloxacin prevention of luminal free endotoxin translocation in mice with cirrhosis. J. Hepatol..

[544] Thompson K., Maltby J., Fallowfield J., McAulay M., Millward-Sadler H., Sheron N. (1998). Interleukin-10 expression and function in experimental murine liver inflammation and fibrosis. Hepatology.

[545] De Souza-Cruz S., Victoria M.B., Tarrago A.M., da Costa A.G., Pimentel J.P., Pires E.F., Araujo Lde P., Coelho-dos-Reis J.G., Gomes Mde S., Amaral L.R. (2016). Liver and blood cytokine microenvironment in HCV patients is associated to liver fibrosis score: A proinflammatory cytokine ensemble orchestrated by TNF and tuned by IL-10. BMC Microbiol..

[546] Melhem A., Muhanna N., Bishara A., Alvarez C.E., Ilan Y., Bishara T., Horani A., Nassar M., Friedman S.L., Safadi R. (2006). Anti-fibrotic activity of NK cells in experimental liver injury through killing of activated HSC. J. Hepatol..

[547] Krizhanovsky V., Yon M., Dickins R.A., Hearn S., Simon J., Miething C., Yee H., Zender L., Lowe S.W. (2008). Senescence of activated stellate cells limits liver fibrosis. Cell.

[548] Gabele E., Muhlbauer M., Dorn C., Weiss T.S., Froh M., Schnabl B., Wiest R., Scholmerich J., Obermeier F., Hellerbrand C. (2008). Role of TLR9 in hepatic stellate cells and experimental liver fibrosis. Biochem. Biophys. Res. Commun..

[549] Lebeaupin C., Proics E., de Bieville C.H., Rousseau D., Bonnafous S., Patouraux S., Adam G., Lavallard V.J., Rovere C., Le Thuc O. (2015). ER stress induces NLRP3 inflammasome activation and hepatocyte death. Cell Death Dis..

[550] Miura K., Kodama Y., Inokuchi S., Schnabl B., Aoyama T., Ohnishi H., Olefsky J.M., Brenner D.A., Seki E. (2010). Toll-like receptor 9 promotes steatohepatitis by induction of interleukin-1beta in mice. Gastroenterology.

[551] Saberi M., Woods N.B., de Luca C., Schenk S., Lu J.C., Bandyopadhyay G., Verma I.M., Olefsky J.M. (2009). Hematopoietic cell-specific deletion of toll-like receptor 4 ameliorates hepatic and adipose tissue insulin resistance in high-fat-fed mice. Cell Metab..

[552] Rivera C.A., Adegboyega P., van Rooijen N., Tagalicud A., Allman M., Wallace M. (2007). Toll-like receptor-4 signaling and Kupffer cells play pivotal roles in the pathogenesis of non-alcoholic steatohepatitis. J. Hepatol..

[553] Minemura M., Shimizu Y. (2015). Gut microbiota and liver diseases. World J. Gastroenterol..

[554] Chen J., Deng X., Liu Y., Tan Q., Huang G., Che Q., Guo J., Su Z. (2020). Kupffer Cells in Non-alcoholic Fatty Liver Disease: Friend or Foe?. Int. J. Biol. Sci..

[555] Pappo I., Bercovier H., Berry E., Gallilly R., Feigin E., Freund H.R. (1995). Antitumor necrosis factor antibodies reduce hepatic steatosis during total parenteral nutrition and bowel rest in the rat. JPEN J. Parenter. Enter. Nutr..

[556] Kirsch R., Clarkson V., Verdonk R.C., Marais A.D., Shephard E.G., Ryffel B., de la M.H.P. (2006). Rodent nutritional model of steatohepatitis: Effects of endotoxin (lipopolysaccharide) and tumor necrosis factor alpha deficiency. J. Gastroenterol. Hepatol..

[557] Jin X., Yu C.H., Lv G.C., Li Y.M. (2007). Increased intestinal permeability in pathogenesis and progress of nonalcoholic steatohepatitis in rats. World J. Gastroenterol..

[558] Imajo K., Fujita K., Yoneda M., Nozaki Y., Ogawa Y., Shinohara Y., Kato S., Mawatari H., Shibata W., Kitani H. (2012). Hyperresponsivity to low-dose endotoxin during progression to nonalcoholic steatohepatitis is regulated by leptin-mediated signaling. Cell Metab..

[559] Miele L., Valenza V., La Torre G., Montalto M., Cammarota G., Ricci R., Masciana R., Forgione A., Gabrieli M.L., Perotti G. (2009). Increased intestinal permeability and tight junction alterations in nonalcoholic fatty liver disease. Hepatology.

[560] Giorgio V., Miele L., Principessa L., Ferretti F., Villa M.P., Negro V., Grieco A., Alisi A., Nobili V. (2014). Intestinal permeability is increased in children with non-alcoholic fatty liver disease, and correlates with liver disease severity. Dig. Liver Dis..

[561] Gasbarrini A., Corazza G.R., Gasbarrini G., Montalto M., Di Stefano M., Basilisco G., Parodi A., Usai-Satta P., Vernia P., Anania C. (2009). Methodology and indications of H2-breath testing in gastrointestinal diseases: The Rome Consensus Conference. Aliment. Pharmacol. Ther..

[562] Gasbarrini A., Lauritano E.C., Gabrielli M., Scarpellini E., Lupascu A., Ojetti V., Gasbarrini G. (2007). Small intestinal bacterial overgrowth: Diagnosis and treatment. Dig. Di.s.

[563] De Wit N.J., Afman L.A., Mensink M., Muller M. (2012). Phenotyping the effect of diet on non-alcoholic fatty liver disease. J. Hepatol..

[564] O’Sullivan A., He X., McNiven E.M., Haggarty N.W., Lonnerdal B., Slupsky C.M. (2013). Early diet impacts infant rhesus gut microbiome, immunity, and metabolism. J. Proteome Res..

[565] Wang H.H., Lee D.K., Liu M., Portincasa P., Wang D.Q.H. (2020). Novel Insights into the Pathogenesis and Management of the Metabolic Syndrome. Pediatric Gastroenterol. Hepatol. Nutr..

[566] Amar J., Lange C., Payros G., Garret C., Chabo C., Lantieri O., Courtney M., Marre M., Charles M.A., Balkau B. (2013). Blood microbiota dysbiosis is associated with the onset of cardiovascular events in a large general population: The D.E.S.I.R. study. PLoS ONE.

[567] Amar J., Serino M., Lange C., Chabo C., Iacovoni J., Mondot S., Lepage P., Klopp C., Mariette J., Bouchez O. (2011). Involvement of tissue bacteria in the onset of diabetes in humans: Evidence for a concept. Diabetologia.

[568] Yun Y., Kim H.N., Lee E.J., Ryu S., Chang Y., Shin H., Kim H.L., Kim T.H., Yoo K., Kim H.Y. (2019). Fecal and blood microbiota profiles and presence of nonalcoholic fatty liver disease in obese versus lean subjects. PLoS ONE.

[569] Raman M., Ahmed I., Gillevet P.M., Probert C.S., Ratcliffe N.M., Smith S., Greenwood R., Sikaroodi M., Lam V., Crotty P. (2013). Fecal microbiome and volatile organic compound metabolome in obese humans with nonalcoholic fatty liver disease. Clin. Gastroenterol. Hepatol..

[570] Caussy C., Hsu C., Lo M.T., Liu A., Bettencourt R., Ajmera V.H., Bassirian S., Hooker J., Sy E., Richards L. (2018). Link between gut-microbiome derived metabolite and shared gene-effects with hepatic steatosis and fibrosis in NAFLD. Hepatology.

[571] Di Ciaula A., Wang D.Q., Molina-Molina E., Lunardi Baccetto R., Calamita G., Palmieri V.O., Portincasa P. (2017). Bile Acids and Cancer: Direct and Environmental-Dependent Effects. Ann. Hepatol..

[572] Grattagliano I., Diogo C.V., Mastrodonato M., de Bari O., Persichella M., Wang D.Q., Liquori A., Ferri D., Carratu M.R., Oliveira P.J. (2013). A silybin-phospholipids complex counteracts rat fatty liver degeneration and mitochondrial oxidative changes. World J. Gastroenterol..

[573] Mastrodonato M., Calamita G., Rossi R., Mentino D., Bonfrate L., Portincasa P., Ferri D., Liquori G.E. (2011). Altered distribution of caveolin-1 in early liver steatosis. Eur. J. Clin. Investig..

[574] Pacelli C., Coluccia A., Grattagliano I., Cocco T., Petrosillo G., Paradies G., De Nitto E., Massaro A., Persichella M., Borracci P. (2010). Dietary choline deprivation impairs rat brain mitochondrial function and behavioral phenotype. J. Nutr..

[575] Petrosillo G., Portincasa P., Grattagliano I., Casanova G., Matera M., Ruggiero F.M., Ferri D., Paradies G. (2007). Mitochondrial dysfunction in rat with nonalcoholic fatty liver Involvement of complex I, reactive oxygen species and cardiolipin. Biochim. Et Biophys. Acta.

[576] Holtmann T.M., Inzaugarat M.E., Knorr J., Geisler L., Schulz M., Bieghs V., Frissen M., Feldstein A.E., Tacke F., Trautwein C. (2021). Bile Acids Activate NLRP3 Inflammasome, Promoting Murine Liver Inflammation or Fibrosis in a Cell Type-Specific Manner. Cells.

[577] Ponziani F.R., Bhoori S., Castelli C., Putignani L., Rivoltini L., Del Chierico F., Sanguinetti M., Morelli D., Sterbini F.P., Petito V. (2019). Hepatocellular Carcinoma Is Associated With Gut Microbiota Profile and Inflammation in Nonalcoholic Fatty Liver Disease. Hepatology.

[578] Levitt M.D., Li R., Demaster E.G., Elson M., Furne J., Levitt D.G. (1997). Use of measurements of ethanol absorption from stomach and intestine to assess human ethanol metabolism. Am. J. Physiol.—Gastrointest. Liver Physiol..

[579] Chen P., Miyamoto Y., Mazagova M., Lee K.C., Eckmann L., Schnabl B. (2015). Microbiota Protects Mice Against Acute Alcohol-Induced Liver Injury. Alcohol. Clin. Exp. Res..

[580] Ansari R.A., Husain K., Rizvi S.A. (2016). Role of Transcription Factors in Steatohepatitis and Hypertension after Ethanol: The Epicenter of Metabolism. Biomolecules.

[581] Hamarneh S.R., Kim B.M., Kaliannan K., Morrison S.A., Tantillo T.J., Tao Q., Mohamed M.M.R., Ramirez J.M., Karas A., Liu W. (2017). Intestinal Alkaline Phosphatase Attenuates Alcohol-Induced Hepatosteatosis in Mice. Dig. Dis. Sci..

[582] Park B., Lee H.R., Lee Y.J. (2016). Alcoholic liver disease: Focus on prodromal gut health. J. Dig. Dis..

[583] Cresci G.A., Glueck B., McMullen M.R., Xin W., Allende D., Nagy L.E. (2017). Prophylactic tributyrin treatment mitigates chronic-binge ethanol-induced intestinal barrier and liver injury. J. Gastroenterol. Hepatol..

[584] Leclercq S., Matamoros S., Cani P.D., Neyrinck A.M., Jamar F., Starkel P., Windey K., Tremaroli V., Backhed F., Verbeke K. (2014). Intestinal permeability, gut-bacterial dysbiosis, and behavioral markers of alcohol-dependence severity. Proc. Natl. Acad. Sci. USA.

[585] Arroyo V., Moreau R., Kamath P.S., Jalan R., Ginès P., Nevens F., Fernández J., To U., García-Tsao G., Schnabl B. (2016). Acute-on-chronic liver failure in cirrhosis. Nat. Rev. Dis. Primers.

[586] Cresci G.A., Bush K., Nagy L.E. (2014). Tributyrin supplementation protects mice from acute ethanol-induced gut injury. Alcohol. Clin. Exp. Res..

[587] Elamin E., Jonkers D., Juuti-Uusitalo K., van Ijzendoorn S., Troost F., Duimel H., Broers J., Verheyen F., Dekker J., Masclee A. (2012). Effects of ethanol and acetaldehyde on tight junction integrity: In vitro study in a three dimensional intestinal epithelial cell culture model. PLoS ONE.

[588] Basuroy S., Sheth P., Mansbach C.M., Rao R.K. (2005). Acetaldehyde disrupts tight junctions and adherens junctions in human colonic mucosa: Protection by EGF and L-glutamine. Am. J. Physiol. Gastrointest. Liver Physiol..

[589] Samak G., Aggarwal S., Rao R.K. (2011). ERK is involved in EGF-mediated protection of tight junctions, but not adherens junctions, in acetaldehyde-treated Caco-2 cell monolayers. Am. J. Physiol.-Gastrointest. Liver Physiol..

[590] Hartmann P., Chen P., Wang H.J., Wang L., McCole D.F., Brandl K., Starkel P., Belzer C., Hellerbrand C., Tsukamoto H. (2013). Deficiency of intestinal mucin-2 ameliorates experimental alcoholic liver disease in mice. Hepatology.

[591] Chen P., Torralba M., Tan J., Embree M., Zengler K., Starkel P., van Pijkeren J.P., DePew J., Loomba R., Ho S.B. (2015). Supplementation of saturated long-chain fatty acids maintains intestinal eubiosis and reduces ethanol-induced liver injury in mice. Gastroenterology.

[592] Kim D.H., Jeong D., Kang I.B., Kim H., Song K.Y., Seo K.H. (2017). Dual function of Lactobacillus kefiri DH5 in preventing high-fat-diet-induced obesity: Direct reduction of cholesterol and upregulation of PPAR-alpha in adipose tissue. Mol. Nutr. Food Res..

[593] Xie G., Zhong W., Zheng X., Li Q., Qiu Y., Li H., Chen H., Zhou Z., Jia W. (2013). Chronic ethanol consumption alters mammalian gastrointestinal content metabolites. J. Proteome Res..

[594] Adachi Y., Moore L.E., Bradford B.U., Gao W., Thurman R.G. (1995). Antibiotics prevent liver injury in rats following long-term exposure to ethanol. Gastroenterology.

[595] Cope K., Risby T., Diehl A.M. (2000). Increased gastrointestinal ethanol production in obese mice: Implications for fatty liver disease pathogenesis. Gastroenterology.

[596] Baraona E., Julkunen R., Tannenbaum L., Lieber C.S. (1986). Role of intestinal bacterial overgrowth in ethanol production and metabolism in rats. Gastroenterology.

[597] Mezey E., Imbembo A.L., Potter J.J., Rent K.C., Lombardo R., Holt P.R. (1975). Endogenous ethanol production and hepatic disease following jejunoileal bypass for morbid obesity. Am. J. Clin. Nutr..

[598] Nair S., Cope K., Risby T.H., Diehl A.M. (2001). Obesity and female gender increase breath ethanol concentration: Potential implications for the pathogenesis of nonalcoholic steatohepatitis. Am. J. Gastroenterol..

[599] Mottaran E., Stewart S.F., Rolla R., Vay D., Cipriani V., Moretti M., Vidali M., Sartori M., Rigamonti C., Day C.P. (2002). Lipid peroxidation contributes to immune reactions associated with alcoholic liver disease. Free Radic. Biol. Med..

[600] Couch R.D., Dailey A., Zaidi F., Navarro K., Forsyth C.B., Mutlu E., Engen P.A., Keshavarzian A. (2015). Alcohol induced alterations to the human fecal VOC metabolome. PLoS ONE.

[601] Kaji H., Asanuma Y., Yahara O., Shibue H., Hisamura M., Saito N., Kawakami Y., Murao M. (1984). Intragastrointestinal alcohol fermentation syndrome: Report of two cases and review of the literature. J. Forensic. Sci. Soc..

[602] Salaspuro M. (1996). Bacteriocolonic pathway for ethanol oxidation: Characteristics and implications. Ann. Med..

[603] Dawes E.A., Foster S.M. (1956). The formation of ethanol in Escherichia coli. Biochim. Et Biophys. Acta.

[604] Engstler A.J., Aumiller T., Degen C., Durr M., Weiss E., Maier I.B., Schattenberg J.M., Jin C.J., Sellmann C., Bergheim I. (2016). Insulin resistance alters hepatic ethanol metabolism: Studies in mice and children with non-alcoholic fatty liver disease. Gut.

[605] Christopherson M.R., Dawson J.A., Stevenson D.M., Cunningham A.C., Bramhacharya S., Weimer P.J., Kendziorski C., Suen G. (2014). Unique aspects of fiber degradation by the ruminal ethanologen Ruminococcus albus 7 revealed by physiological and transcriptomic analysis. BMC Genom..

[606] Setshedi M., Wands J.R., Monte S.M. (2010). Acetaldehyde adducts in alcoholic liver disease. Oxidative Med. Cell. Longev..

[607] Ni Y.H., Huo L.J., Li T.T. (2017). Effect of interleukin-22 on proliferation and activation of hepatic stellate cells induced by acetaldehyde and related mechanism. Zhonghua Gan Zang Bing Za Zhi.

[608] Wu X., Wang Y., Wang S., Xu R., Lv X. (2017). Purinergic P2X7 receptor mediates acetaldehyde-induced hepatic stellate cells activation via PKC-dependent GSK3beta pathway. Int. Immunopharmacol..

[609] López-Lázaro M. (2016). A local mechanism by which alcohol consumption causes cancer. Oral Oncol..

[610] Baker S.S., Baker R.D., Liu W., Nowak N.J., Zhu L. (2010). Role of alcohol metabolism in non-alcoholic steatohepatitis. PLoS ONE.

[611] Ahuja M., Schwartz D.M., Tandon M., Son A., Zeng M., Swaim W., Eckhaus M., Hoffman V., Cui Y., Xiao B. (2017). Orai1-Mediated Antimicrobial Secretion from Pancreatic Acini Shapes the Gut Microbiome and Regulates Gut Innate Immunity. Cell Metab..

[612] De Aguiar Vallim T.Q., Tarling E.J., Edwards P.A. (2013). Pleiotropic roles of bile acids in metabolism. Cell Metab..

[613] Albaugh V.L., Banan B., Antoun J., Xiong Y., Guo Y., Ping J., Alikhan M., Clements B.A., Abumrad N.N., Flynn C.R. (2019). Role of Bile Acids and GLP-1 in Mediating the Metabolic Improvements of Bariatric Surgery. Gastroenterology.

[614] Thoni V., Pfister A., Melmer A., Enrich B., Salzmann K., Kaser S., Lamina C., Ebenbichler C.F., Hackl H., Tilg H. (2017). Dynamics of Bile Acid Profiles, GLP-1, and FGF19 After Laparoscopic Gastric Banding. J. Clin. Endocrinol. Metab..

[615] Mudaliar S., Henry R.R., Sanyal A.J., Morrow L., Marschall H.U., Kipnes M., Adorini L., Sciacca C.I., Clopton P., Castelloe E. (2013). Efficacy and safety of the farnesoid X receptor agonist obeticholic acid in patients with type 2 diabetes and nonalcoholic fatty liver disease. Gastroenterology.

[616] Ridlon J.M., Kang D.J., Hylemon P.B. (2006). Bile salt biotransformations by human intestinal bacteria. J. Lipid Res..

[617] Sayin S.I., Wahlstrom A., Felin J., Jantti S., Marschall H.U., Bamberg K., Angelin B., Hyotylainen T., Oresic M., Backhed F. (2013). Gut microbiota regulates bile acid metabolism by reducing the levels of tauro-beta-muricholic acid, a naturally occurring FXR antagonist. Cell Metab..

[618] Yokota A., Fukiya S., Islam K.B., Ooka T., Ogura Y., Hayashi T., Hagio M., Ishizuka S. (2012). Is bile acid a determinant of the gut microbiota on a high-fat diet?. Gut Microbes.

[619] Parseus A., Sommer N., Sommer F., Caesar R., Molinaro A., Stahlman M., Greiner T.U., Perkins R., Backhed F. (2017). Microbiota-induced obesity requires farnesoid X receptor. Gut.

[620] Li F., Jiang C., Krausz K.W., Li Y., Albert I., Hao H., Fabre K.M., Mitchell J.B., Patterson A.D., Gonzalez F.J. (2013). Microbiome remodelling leads to inhibition of intestinal farnesoid X receptor signalling and decreased obesity. Nat. Commun..

[621] Cao H., Xu M., Dong W., Deng B., Wang S., Zhang Y., Wang S., Luo S., Wang W., Qi Y. (2017). Secondary bile acid-induced dysbiosis promotes intestinal carcinogenesis. Int. J. Cancer.

[622] Jiang C., Xie C., Li F., Zhang L., Nichols R.G., Krausz K.W., Cai J., Qi Y., Fang Z.Z., Takahashi S. (2015). Intestinal farnesoid X receptor signaling promotes nonalcoholic fatty liver disease. J. Clin. Investig..

[623] Ferslew B.C., Xie G., Johnston C.K., Su M., Stewart P.W., Jia W., Brouwer K.L., Barritt A.S.t. (2015). Altered Bile Acid Metabolome in Patients with Nonalcoholic Steatohepatitis. Dig. Dis. Sci..

[624] Jiao N., Baker S.S., Chapa-Rodriguez A., Liu W., Nugent C.A., Tsompana M., Mastrandrea L., Buck M.J., Baker R.D., Genco R.J. (2018). Suppressed hepatic bile acid signalling despite elevated production of primary and secondary bile acids in NAFLD. Gut.

[625] Mouzaki M., Wang A.Y., Bandsma R., Comelli E.M., Arendt B.M., Zhang L., Fung S., Fischer S.E., McGilvray I.G., Allard J.P. (2016). Bile Acids and Dysbiosis in Non-Alcoholic Fatty Liver Disease. PLoS ONE.

[626] Duncan S.H., Louis P., Thomson J.M., Flint H.J. (2009). The role of pH in determining the species composition of the human colonic microbiota. Environ. Microbiol..

[627] Sawicki C.M., Livingston K.A., Obin M., Roberts S.B., Chung M., McKeown N.M. (2017). Dietary Fiber and the Human Gut Microbiota: Application of Evidence Mapping Methodology. Nutrients.

[628] Koh A., De Vadder F., Kovatcheva-Datchary P., Bäckhed F. (2016). From dietary fiber to host physiology: Short-chain fatty acids as key bacterial metabolites. Cell.

[629] Zhao Y., Wu J., Li J.V., Zhou N.Y., Tang H., Wang Y. (2013). Gut microbiota composition modifies fecal metabolic profiles in mice. J. Proteome Res..

[630] Brown A.J., Goldsworthy S.M., Barnes A.A., Eilert M.M., Tcheang L., Daniels D., Muir A.I., Wigglesworth M.J., Kinghorn I., Fraser N.J. (2003). The Orphan G protein-coupled receptors GPR41 and GPR43 are activated by propionate and other short chain carboxylic acids. J. Biol. Chem..

[631] Bellahcene M., O’Dowd J.F., Wargent E.T., Zaibi M.S., Hislop D.C., Ngala R.A., Smith D.M., Cawthorne M.A., Stocker C.J., Arch J.R. (2013). Male mice that lack the G-protein-coupled receptor GPR41 have low energy expenditure and increased body fat content. Br. J. Nutr..

[632] Rau M., Rehman A., Dittrich M., Groen A.K., Hermanns H.M., Seyfried F., Beyersdorf N., Dandekar T., Rosenstiel P., Geier A. (2018). Fecal SCFAs and SCFA-producing bacteria in gut microbiome of human NAFLD as a putative link to systemic T-cell activation and advanced disease. United Eur. Gastroenterol. J..

[633] Weidemann M.J., Hems R., Williams D.L., Spray G.H., Krebs H.A. (1970). Gluconeogenesis from propionate in kidney and liver of the vitamin B12-deficient rat. Biochem. J..

[634] Mattace Raso G., Simeoli R., Russo R., Iacono A., Santoro A., Paciello O., Ferrante M.C., Canani R.B., Calignano A., Meli R. (2013). Effects of sodium butyrate and its synthetic amide derivative on liver inflammation and glucose tolerance in an animal model of steatosis induced by high fat diet. PLoS ONE.

[635] Jin C.J., Sellmann C., Engstler A.J., Ziegenhardt D., Bergheim I. (2015). Supplementation of sodium butyrate protects mice from the development of non-alcoholic steatohepatitis (NASH). Br. J. Nutr..

[636] Ilan Y., Maron R., Tukpah A.M., Maioli T.U., Murugaiyan G., Yang K., Wu H.Y., Weiner H.L. (2010). Induction of regulatory T cells decreases adipose inflammation and alleviates insulin resistance in ob/ob mice. Proc. Natl. Acad. Sci. USA.

[637] Cipolletta D., Feuerer M., Li A., Kamei N., Lee J., Shoelson S.E., Benoist C., Mathis D. (2012). PPAR-gamma is a major driver of the accumulation and phenotype of adipose tissue Treg cells. Nature.

[638] Feuerer M., Herrero L., Cipolletta D., Naaz A., Wong J., Nayer A., Lee J., Goldfine A.B., Benoist C., Shoelson S. (2009). Lean, but not obese, fat is enriched for a unique population of regulatory T cells that affect metabolic parameters. Nat. Med..

[639] Tao R., de Zoeten E.F., Ozkaynak E., Chen C., Wang L., Porrett P.M., Li B., Turka L.A., Olson E.N., Greene M.I. (2007). Deacetylase inhibition promotes the generation and function of regulatory T cells. Nat. Med..

[640] Mehedint M.G., Zeisel S.H. (2013). Choline’s role in maintaining liver function: New evidence for epigenetic mechanisms. Curr. Opin. Clin. Nutr. Metab. Care.

[641] Grattagliano I., Caraceni P., Portincasa P., Domenicali M., Palmieri V.O., Trevisani F., Bernardi M., Palasciano G. (2003). Adaptation of subcellular glutathione detoxification system to stress conditions in choline-deficient diet induced rat fatty liver. Cell Biol. Toxicol..

[642] Xie G., Yan A., Lin P., Wang Y., Guo L. (2021). Trimethylamine N-oxide-a marker for atherosclerotic vascular disease. Rev. Cardiovasc. Med..

[643] Cretoiu D., Ionescu R.F., Enache R.M., Cretoiu S.M., Voinea S.C. (2021). Gut Microbiome, Functional Food, Atherosclerosis, and Vascular Calcifications-Is There a Missing Link?. Microorganisms.

[644] Jiang S., Shui Y., Cui Y., Tang C., Wang X., Qiu X., Hu W., Fei L., Li Y., Zhang S. (2021). Gut microbiota dependent trimethylamine N-oxide aggravates angiotensin II-induced hypertension. Redox. Biol..

[645] Shen X., Li L., Sun Z., Zang G., Zhang L., Shao C., Wang Z. (2021). Gut Microbiota and Atherosclerosis-Focusing on the Plaque Stability. Front. Cardiovasc. Med..

[646] Tang W.H., Wang Z., Levison B.S., Koeth R.A., Britt E.B., Fu X., Wu Y., Hazen S.L. (2013). Intestinal microbial metabolism of phosphatidylcholine and cardiovascular risk. New Engl. J. Med..

[647] Tilg H. (2016). A Gut Feeling about Thrombosis. New Engl. J. Med..

[648] Loscalzo J. (2013). Gut microbiota, the genome, and diet in atherogenesis. New Engl. J. Med..

[649] Tang W.H., Kitai T., Hazen S.L. (2017). Gut Microbiota in Cardiovascular Health and Disease. Circ. Res..

[650] Koeth R.A., Wang Z., Levison B.S., Buffa J.A., Org E., Sheehy B.T., Britt E.B., Fu X., Wu Y., Li L. (2013). Intestinal microbiota metabolism of L-carnitine, a nutrient in red meat, promotes atherosclerosis. Nat. Med..

[651] Wang Z., Klipfell E., Bennett B.J., Koeth R., Levison B.S., Dugar B., Feldstein A.E., Britt E.B., Fu X., Chung Y.M. (2011). Gut flora metabolism of phosphatidylcholine promotes cardiovascular disease. Nature.

[652] Zhu W., Gregory J.C., Org E., Buffa J.A., Gupta N., Wang Z., Li L., Fu X., Wu Y., Mehrabian M. (2016). Gut Microbial Metabolite TMAO Enhances Platelet Hyperreactivity and Thrombosis Risk. Cell.

[653] Chen Y.M., Liu Y., Zhou R.F., Chen X.L., Wang C., Tan X.Y., Wang L.J., Zheng R.D., Zhang H.W., Ling W.H. (2016). Associations of gut-flora-dependent metabolite trimethylamine-N-oxide, betaine and choline with non-alcoholic fatty liver disease in adults. Sci. Rep..

[654] Li P., Zhong C., Li S., Sun T., Huang H., Chen X., Zhu Y., Hu X., Peng X., Zhang X. (2018). Plasma concentration of trimethylamine-N-oxide and risk of gestational diabetes mellitus. Am. J. Clin. Nutr..

[655] Tang W.H., Wang Z., Li X.S., Fan Y., Li D.S., Wu Y., Hazen S.L. (2017). Increased Trimethylamine N-Oxide Portends High Mortality Risk Independent of Glycemic Control in Patients with Type 2 Diabetes Mellitus. Clin. Chem..

[656] Shan Z., Sun T., Huang H., Chen S., Chen L., Luo C., Yang W., Yang X., Yao P., Cheng J. (2017). Association between microbiota-dependent metabolite trimethylamine-N-oxide and type 2 diabetes. Am. J. Clin. Nutr..

[657] Dumas M.E., Barton R.H., Toye A., Cloarec O., Blancher C., Rothwell A., Fearnside J., Tatoud R., Blanc V., Lindon J.C. (2006). Metabolic profiling reveals a contribution of gut microbiota to fatty liver phenotype in insulin-resistant mice. Proc. Natl. Acad. Sci. USA.

[658] Spencer M.D., Hamp T.J., Reid R.W., Fischer L.M., Zeisel S.H., Fodor A.A. (2011). Association between composition of the human gastrointestinal microbiome and development of fatty liver with choline deficiency. Gastroenterology.

[659] Velasquez M.T., Ramezani A., Manal A., Raj D.S. (2016). Trimethylamine N-Oxide: The Good, the Bad and the Unknown. Toxins.

[660] Hoyles L., Fernandez-Real J.M., Federici M., Serino M., Abbott J., Charpentier J., Heymes C., Luque J.L., Anthony E., Barton R.H. (2018). Molecular phenomics and metagenomics of hepatic steatosis in non-diabetic obese women. Nat. Med..

[661] Koh A., Molinaro A., Stahlman M., Khan M.T., Schmidt C., Manneras-Holm L., Wu H., Carreras A., Jeong H., Olofsson L.E. (2018). Microbially Produced Imidazole Propionate Impairs Insulin Signaling through mTORC1. Cell.

[662] Diehl A.M., Li Z.P., Lin H.Z., Yang S.Q. (2005). Cytokines and the pathogenesis of non-alcoholic steatohepatitis. Gut.

[663] Vetrano S., Rescigno M., Cera M.R., Correale C., Rumio C., Doni A., Fantini M., Sturm A., Borroni E., Repici A. (2008). Unique role of junctional adhesion molecule-a in maintaining mucosal homeostasis in inflammatory bowel disease. Gastroenterology.

[664] Monteiro A.C., Sumagin R., Rankin C.R., Leoni G., Mina M.J., Reiter D.M., Stehle T., Dermody T.S., Schaefer S.A., Hall R.A. (2013). JAM-A associates with ZO-2, afadin, and PDZ-GEF1 to activate Rap2c and regulate epithelial barrier function. Mol. Biol. Cell.

[665] Menard S., Cerf-Bensussan N., Heyman M. (2010). Multiple facets of intestinal permeability and epithelial handling of dietary antigens. Mucosal. Immunol..

[666] Laukoetter M.G., Nava P., Lee W.Y., Severson E.A., Capaldo C.T., Babbin B.A., Williams I.R., Koval M., Peatman E., Campbell J.A. (2007). JAM-A regulates permeability and inflammation in the intestine in vivo. J. Exp. Med..

[667] Philips C.A., Pande A., Shasthry S.M., Jamwal K.D., Khillan V., Chandel S.S., Kumar G., Sharma M.K., Maiwall R., Jindal A. (2017). Healthy Donor Fecal Microbiota Transplantation in Steroid-Ineligible Severe Alcoholic Hepatitis: A Pilot Study. Clin. Gastroenterol. Hepatol..

[668] Nazim M., Stamp G., Hodgson H.J. (1989). Non-alcoholic steatohepatitis associated with small intestinal diverticulosis and bacterial overgrowth. Hepatogastroenterology.

[669] Lichtman S.N., Sartor R.B., Keku J., Schwab J.H. (1990). Hepatic inflammation in rats with experimental small intestinal bacterial overgrowth. Gastroenterology.

[670] Lichtman S.N., Keku J., Schwab J.H., Sartor R.B. (1991). Hepatic injury associated with small bowel bacterial overgrowth in rats is prevented by metronidazole and tetracycline. Gastroenterology.

[671] Kapil S., Duseja A., Sharma B.K., Singla B., Chakraborti A., Das A., Ray P., Dhiman R.K., Chawla Y. (2016). Small intestinal bacterial overgrowth and toll-like receptor signaling in patients with non-alcoholic fatty liver disease. J. Gastroenterol. Hepatol..

[672] Farrell G.C., Larter C.Z. (2006). Nonalcoholic fatty liver disease: From steatosis to cirrhosis. Hepatology.

[673] DeMeo M.T., Mutlu E.A., Keshavarzian A., Tobin M.C. (2002). Intestinal permeation and gastrointestinal disease. J. Clin. Gastroenterol..

[674] Arslan G., Atasever T., Cindoruk M., Yildirim I.S. (2001). (51)CrEDTA colonic permeability and therapy response in patients with ulcerative colitis. Nucl. Med. Commun..

[675] Ponziani F.R., Zocco M.A., Cerrito L., Gasbarrini A., Pompili M. (2018). Bacterial translocation in patients with liver cirrhosis: Physiology, clinical consequences, and practical implications. Expert Rev. Gastroenterol. Hepatol..

[676] Nier A., Engstler A.J., Maier I.B., Bergheim I. (2017). Markers of intestinal permeability are already altered in early stages of non-alcoholic fatty liver disease: Studies in children. PLoS ONE.

[677] Cariello R., Federico A., Sapone A., Tuccillo C., Scialdone V.R., Tiso A., Miranda A., Portincasa P., Carbonara V., Palasciano G. (2010). Intestinal permeability in patients with chronic liver diseases: Its relationship with the aetiology and the entity of liver damage. Dig. Liver Dis..

[678] Assimakopoulos S.F., Tsamandas A.C., Tsiaoussis G.I., Karatza E., Triantos C., Vagianos C.E., Spiliopoulou I., Kaltezioti V., Charonis A., Nikolopoulou V.N. (2012). Altered intestinal tight junctions’ expression in patients with liver cirrhosis: A pathogenetic mechanism of intestinal hyperpermeability. Eur. J. Clin. Investig..

[679] Fukui H., Brauner B., Bode J.C., Bode C. (1991). Plasma endotoxin concentrations in patients with alcoholic and non-alcoholic liver disease: Reevaluation with an improved chromogenic assay. J. Hepatol..

